# Profiling the Hsp70 Chaperone Network in Heat-Induced Proteotoxic Stress Models of Human Neurons

**DOI:** 10.3390/biology12030416

**Published:** 2023-03-09

**Authors:** Bothina Mohammed Alharbi, Tahani H. Albinhassan, Razan Ali Alzahrani, Abderrezak Bouchama, Sameer Mohammad, Awatif Abdulaziz Alomari, May Nasser Bin-Jumah, Entissar S. AlSuhaibani, Shuja Shafi Malik

**Affiliations:** 1 Experimental Medicine Department, King Abdullah International Medical Research Center, King Saud bin Abdulaziz University for Health Sciences, Ministry of National Guard Health Affairs, Riyadh 11426, Saudi Arabia; 2 Zoology Department, College of Science, King Saud University, Riyadh 12372, Saudi Arabia; 3 Biology Department, College of Science, Princess Nourah bint Abdulrahman University, Riyadh 11564, Saudi Arabia

**Keywords:** proteostasis, heat shock, chaperones, Hsp70 proteins, stress response, neurons

## Abstract

**Simple Summary:**

Rising temperatures and consequent heat waves are detrimental manifestations of climate change for the population’s health. Exposure to extreme environmental heat during episodes of intense and long-lasting heat waves is a direct cause of heat-related illnesses, particularly life-threatening heat stroke. A harmful manifestation of heat stroke is damage to the central nervous system, particularly the brain, which can result in permanent disability in approximately a third of patients. Heat can damage biomolecules, particularly proteins, because of their high vulnerability to changes in cellular conditions such as temperature. Temperature-induced protein damage leads to misfolding and clumping into aggregates that are the neurotoxic agents for most neurodegenerative disorders, including heat-induced damage, with certain defining features for later. The cellular response to these processes is to increase the production of a subset of helpful proteins called heat shock proteins 70 (Hsp70). Here, we report that hyperthermia induces severe brain proteotoxic stress in different neuronal models. Likewise, we showed a differential vulnerability among neuronal cells, with more damage in mature than young cells. As part of the stress response, we report the identification, differential importance, and co-expression of Hsp70 chaperones and their interacting partners in the human neuronal models of heat-induced damage. These findings support the idea that proteotoxic stress may play a role in heat stroke-induced brain damage. This study also helps expand our knowledge about stress response during neurodegenerative damage and is essential in developing therapeutic approaches.

**Abstract:**

Heat stroke is among the most hazardous hyperthermia-related illnesses and an emerging threat to humans from climate change. Acute brain injury and long-lasting brain damage are the hallmarks of this condition. Hyperthermic neurological manifestations are remarkable for their damage correlation with stress amplitude and long-term persistence. Hyperthermia-induced protein unfolding, and nonspecific aggregation accumulation have neurotoxic effects and contribute to the pathogenesis of brain damage in heat stroke. Therefore, we generated heat-induced, dose-responsive extreme and mild proteotoxic stress models in medulloblastoma [Daoy] and neuroblastoma [SH-SY5Y] and differentiated SH-SY5Y neuronal cells. We show that heat-induced protein aggregation is associated with reduced cell proliferation and viability. Higher protein aggregation in differentiated neurons than in neuroblastoma precursors suggests a differential neuronal vulnerability to heat. We characterized the neuronal heat shock response through RT-PCR array analysis of eighty-four genes involved in protein folding and protein quality control (PQC). We identify seventeen significantly expressed genes, five of which are Hsp70 chaperones, and four of their known complementing function proteins. Protein expression analysis determined the individual differential contribution of the five Hsp70 chaperones to the proteotoxic stress response and the significance of only two members under mild conditions. The co-expression analysis reveals significantly high co-expression between the Hsp70 chaperones and their interacting partners. The findings of this study lend support to the hypothesis that hyperthermia-induced proteotoxicity may underlie the brain injury of heat stroke. Additionally, this study presents a comprehensive map of the Hsp70 network in these models with potential clinical and translational implications.

## 1. Introduction

The global temperature rise is one of climate change’s most detrimental outcomes [1]. Elevated environmental temperature is a natural stressor that impacts a multitude of physiological functions and induces pathophysiological responses [2,3]. It can lead to heat stroke, a life-threatening condition defined by extreme hyperthermia with core body temperature usually greater than 40.5 °C and central nervous system dysfunction such as delirium, convulsions, and coma [3,4,5]. Proteins are among the most thermosensitive cellular components due to their evolutionary optimization to function in narrow growth temperature ranges [6]. Even a modest temperature increase leads to protein unfolding and nonspecific aggregation, and many of the harmful effects of heat stress are attributed to this protein aggregation. This protein aggregation is a known inducer of neurotoxic effects and a causative factor for multiple neurodegenerative disorders [7]. Therefore, a similar proteotoxicity may be at play in heat stroke.

The proteostasis (protein homeostasis) network coordinates proteome balance maintenance by regulating the protein life cycle, which includes protein synthesis, folding, conformational maintenance, and degradation [8,9,10]. This serves a dual purpose, guaranteeing a properly functional and operational proteome under normal conditions while simultaneously ensuring a bulwark against deleterious cell conditions [9,10,11]. Among the critical components of the proteostasis machinery are 70 kDa heat shock proteins (Hsp70s), a ubiquitous chaperone group involved in protein folding/refolding and irreversible aggregate clearance processes [9,12]. Humans express thirteen homologs of Hsp70 in a spatio-temporal manner, with their activities regulated according to cellular requirements, such as during proteotoxic stress. Like other chaperones, Hsp70s exhibit little specificity but show a high level of adaptation in their functional properties, partially attributed to their interactions with other complementing functions [9,13,14]. Due to these characteristics and the involvement of Hsp70 in different phases of the protein life cycle, its stress inducibility becomes crucial in maintaining cell health [12].

Here, we used three different heat-induced proteotoxic stress models of human neurons: medulloblastoma [Daoy], neuroblastoma [SH-SY5Y], and differentiated SH-SY5Y neuronal cells. Medulloblastoma cells are undifferentiated progenitor cells that form tumors predominantly in the cerebellum. Neuroblastoma cells represent immature nerve cells that are synthetically differentiated into terminal neurons. Using these three cell types ensures reasonable representation to study neuronal proteotoxic stress responses. We examined aggregation and cell proliferation as markers of proteotoxicity. Following this, we analyzed the protective heat shock response using the quantitative gene expression, protein expression, and co-expression analysis of members of the Hsp70 family and their interacting partners. Aggregate accumulation is known to cause severe and chronic neurological disorders, but the rate of aggregate accumulation is much higher in the case of hyperthermia [15]. The persistent neurological defect is a well-described manifestation of heat-induced hyperthermia [15,16], and the extent of hyperthermia-induced neuronal damage correlates with its magnitude and duration [5,15]. These pertinent factors make it imperative to rely on heat-induced proteotoxic stress over other stressors that elicit a similar cell response [6]. Therefore, we performed this study under two conditions classified as *extreme* and *mild* heat stress. We analyzed the stress response in a time series over 24 h. Using an 84-gene RT-PCR array, we evaluated the overexpression of the Hsp70 family and other associated genes involved in protein folding and the response to heat shock. After that, we perform an analysis at the protein level of five very important Hsp70 family proteins and four of their known interacting partners. Extreme and mild heat stress make it clear that different Hsp70 proteins play different roles and that only two of the five Hsp70s are important in mild heat stress. The co-expression of highly overexpressed Hsp70 proteins and their interacting partners is significantly correlated. This represents a highly comprehensive map of the Hsp70 network in heat-induced proteotoxic stress models of human neurons. We believe that this result sheds significant new light on the role of these proteins and goes a long way toward contributing to the development of targeted therapeutic strategies [17,18,19].

## 2. Materials and Methods

### 2.1. Cell Culture and Differentiation

Human neuroblastoma SH-SY5Y [CRL-2266] and human medulloblastoma Daoy [HTB-186] were obtained from the American Type Culture Collection (ATCC), Manassas, VA, USA. Cells were maintained in a complete growth medium (DMEM-F12) containing 10% fetal bovine serum (FBS; Life Technologies, Grand Island, NY, USA) and 4 mM L-glutamine, 100 μg/mL streptomycin, and 100 U/mL penicillin at 37 °C in 100 mm dishes in a humidified 5% CO_2_ atmosphere. Neuronal differentiation was induced by treating SH-SY5Y neuroblastoma with 10 μM all-trans-retinoic acid (R265; Sigma Aldrich, St. Louis, MO, USA) for a minimum of 72 h under serum-free conditions [20,21]. Post-differentiation, clear differentiating morphological differences are observed (Figure S1).

### 2.2. Induction of Heat Stress

Heat stress was induced in ~60% of confluent cells by transferring them from 37 °C to the temperature to be tested. At the zero time-point, all dishes (with 60–65% confluent cells) were transferred to the heat stress temperature other than the control that continued to grow at 37 °C. The exposure temperatures termed “exposure temperature” [ET] tested were 39, 42, 44, and 46 °C, and the cells were heat exposed as defined by exposure duration [ED] for 1, 2, and 3 h. After the stress exposure, the cells were transferred back to 37 °C. Cells were harvested at a designated time (0 h, 1 h, 6 h, 12 h, and 24 h) post-stress exposure and processed according to the requirements of the next step. Briefly, heat-stressed and control cells were washed twice with PBS and trypsinized. Cells were centrifuged at 4000× *g* for 5 min, the supernatant was discarded, and pellets were frozen for subsequent analyses. The goal of testing multiple heat stresses is to identify conditions that represent different magnitudes of heat stresses based on a combination of exposure time [ET] and exposure duration [ED].

### 2.3. Protein Aggregation Assay

Protein aggregation was measured using the 96-well Protein Aggregation Assay Kit (Cat No.: ab234048) supplied by Abcam (Discovery Drive, Cambridge Biomedical Campus, Cambridge, UK). The assay requires a total of 50–100 ug of protein per well, and the samples were read in triplicate. Cellular protein samples from three experiments were assayed. Protein extraction for aggregation involves the use of freezing and thawing cycles to avoid interference from the detergents in standard cell lysis buffers. Control samples grown at 37 °C were used as the reference point, and the results are presented as the percentage increase in fluorescence.

### 2.4. Cell Proliferation Assays

The impact of heat-stressed cells on viability and proliferation was evaluated using the xCELLigence Real-Time Cell Analyser (RTCA-DP version; Roche Diagnostics, Mannheim, Germany). This device continuously monitors cellular adherence, recording label-free changes in *electrical impedance*, and is stated as arbitrary units called Cell Index (CI), which are correlated to cell number, morphology, size, and the strength of cell attachment to the plate surface [22]. An initial cell density titration was performed to identify the optimum cell number. Titrations at 5, 10, 20, 40, 60, 80, and 100,000 cells/well were conducted with 10,000 cells in the case of Daoy and 100,000 cells in the case of SH-SY5Y, and the differentiated SH-SY5Y was found to be the ideal cell density for seeding. E-16 plate wells were equilibrated with the culture medium, and background measurements were taken. The trypsinized cells were counted using trypan blue, resuspended in the culture medium, plated at the appropriate density/well in fresh medium to a final volume of 200 µL and incubated for 30 min at 37 °C and 5% CO_2_ in the RTCA cradle. Impedance signals were recorded every 10 min for 72 h in control cells grown at 37 °C and heat-exposed cells.

### 2.5. RNA Isolation and cDNA Synthesis

RNA was isolated from the stored cell pellets utilizing the mini RNeasy Mini Kit (Qiagen, Germantown, MD, USA). RNA integrity and yield were analyzed using the Nano Drop (Thermo Fischer, Wilmington, DE, USA). 2 µg of the total RNA was transcribed into cDNA for all gene arrays and RT-PCR experiments using the FastLane Cell cDNA Kit (Qiagen, Germantown, MD, USA).

### 2.6. Gene Arrays and RT-PCR

For heat-stress model characterization, the initial monitoring of the individual Hsp70 gene expression was performed utilizing the primer sequences, which were obtained from the Harvard Primer Bank repository [23]. The primers used were as follows: HSPA1B, 5′-GCGAGGCGGACAAGAAGAA-3′ (forward) and 5′-GATGGGGTTACACACCTGCT-3′ (reverse); GAPDH, 5′-GGAGCGAGATCCCTCCAAAAT-3′ (forward), and 5′-GGCTGTTGTCATACTTCTCATGG-3′ (reverse). We performed a comprehensive analysis of the heat shock protein genes using the RT^2^ Profiler TM Human Heat Shock Proteins and Chaperones PCR Array (Cat. No. 330231PAHS-076ZA Qiagen, Germantown, MD, USA) (see Table S1 for a gene list). This array helps profile eighty-four heat shock protein genes’ expression simultaneously and offers the ability to evaluate results using five endogenous controls simultaneously. Moreover, β-actin and GAPDH were used as controls during data analysis.

These quantitative gene expression measurements were carried out with RT-polymerase chain reaction using Platinum PCR SuperMix (Thermo Fisher Scientific, Baltics, Vilnius, Lithuania) in triplicate with SYBR Green PCR Mix (Qiagen, Germantown, MD, USA).

### 2.7. Protein Extraction and Concentration Determination

Cellular proteins were extracted by RIPA Lysis and Extraction Buffer (Thermo Scientific™, Rockford, IL, USA, Cat. No. 89900). Cells were completely resuspended in RIPA buffer, vortexed, and incubated on ice for 30 min before centrifugation at 10,000× *g* for 20–30 min at 4 °C to separate cell debris. Quantification was performed using Pierce™ BCA Protein Assay Kit (Thermo Scientific™, Rockford, IL, USA). Protein concentrations were determined using a 96-well format and evaluated with reference to a standard such as bovine serum albumin (BSA).

### 2.8. Immunoblotting

Equal protein quantities were run on 10–15% Sodium Dodecyl Sulfate Polyacrylamide Gel and transferred onto a nitrocellulose membrane using a Mini transblot system (Bio-Rad, Hercules, CA, USA). The quantity of input protein and antibody dilution were critical to achieving high-resolution western blots. 5 μg total protein worked optimally for HSPA1A and HSPA6; 10 μg for HSPA1B, HSPH1, and HSPA1L; 15 μg for BAG3; 20 μg for HSPA4L and DNAJB1; 100 μg for DNAJA4. The antibodies were used at dilutions anti-HSPA1A (Invitrogen™, Rockford, IL, USA: PA5-28003) at 1:7000–14,000, anti-HSPA1B (Invitrogen™, Rockford, IL, USA: PA5-28369) at 1:4000, anti-HSPA1L (Sigma Aldrich, St. Louis, MO, USA: HPA043285) at 1:3000, anti-HSPA4L (Sigma Aldrich, St. Louis, MO, USA: AV53693) at 1:1000–3000, anti-HSPA6 (Sigma Aldrich, St. Louis, MO, USA: HPA028546) at 1:3000, anti-HSPH1 (Invitrogen™, Rockford, IL, USA: PA5-99283) at 1:1000, anti-BAG3 (Sigma Aldrich, St. Louis, MO, USA: HPA020586) at 1:3000, anti-DNAJA4 (Sigma Aldrich, St. Louis, MO, USA: HPA063247) at 1:500, and anti-DNAJB1 (Sigma Aldrich, St. Louis, MO, USA: HPA041790) at 1:3000. Immunoblotting was performed by probing the membranes with primary antibodies overnight at 4 °C, followed by probing with specific horseradish peroxidase (HRP)-conjugated secondary antibodies. The blots were visualized using SuperSignal™ West Femto Chemiluminescent Substrate (Thermo Fisher Scientific, Waltham, MA, USA) in a ChemiDoc visualization system (Bio-Rad, Hercules, CA, USA). The image analyzing software ImageJ [24] performed densitometry of the bands, which were normalized by protein levels of GAPDH (Santa Cruz Biotechnology, Inc., Dallas, USA: sc-47724). All the western blot images are available as File S1.

### 2.9. Expression Analysis

Gene and protein expression data are presented as mean ± standard deviation (SD). Statistical differences between the groups analyzed were evaluated by applying an unpaired t-test and a one-way analysis of variance (ANOVA) using GraphPad Prism 7.0 software (GraphPad Software Inc. La Jolla, CA, USA). *p*-values < 0.05 are considered statistically significant. Unless reported otherwise, all the results are from three independent experiments.

### 2.10. Expression Kinetics Analysis

Gene and protein expression kinetics analysis was performed by inputting the averaged expression Log_2_ fold-change in heatmap plots or smoothened scatterplots. Ratios were calculated utilizing the actual fold-change expression data. Ratios were calculated utilizing the actual fold-change expression data.

### 2.11. Co-Expression Analysis

We used the GeneMANIA database to perform co-expression network analysis to understand the interactions between differentially expressed genes (DEGs) identified in our array analysis [24]. *Homo sapiens* was selected as the target organism, and the gene overexpression data from the RT^2^ Profiler was used as input. The co-expression analysis was performed for each cell type separately. The interaction analysis was limited to co-expression by selectively opting for this option during the analysis.

## 3. Results

### 3.1. Heat-Induced Proteotoxic Stress

**Figure 1:**
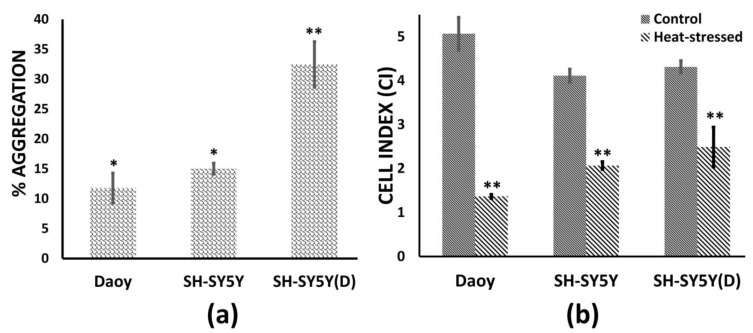
Protein aggregation and cell proliferation in heat stress models (ET: 44 °C, ED: 2 h) of medulloblastoma [Daoy], neuroblastoma [SH-SY5Y], and differentiated SH-SY5Y [SH-SY5Y(D)]. (**a**) Protein aggregation is measured immediately after heat stress, that is, recovery time 0 h. The percentage change in aggregation of heat-stressed cells in comparison to the proteome of control cells (37 °C) corresponds to the percentage change in measured fluorescence. (**b**) The cell index (CI) values depicted here are 24 h after heat stress and show a measurable reduction in the three cell types. Statistically significant stress is measured both in protein aggregation and cell proliferation (* *p* < 0.05 ** *p* < 0.01).

This study aimed at analyzing chaperone behavior over a relatively broad spectrum of stress conditions. Exposure temperatures [ET] of 39, 42, 44, and 46 °C and exposure durations [ED] of 1, 2, and 3 h formed part of the heat-stress induction protocol. Stress exposure involving 46 °C and 44 °C for 3 h resulted in cell death as measured by trypan blue dye uptake. The cells were tolerant to 44 °C for up to 2 h of exposure. Thus, 44 °C represents a maximal exposure time [ET] and two hours a maximal exposure duration [ED]. After stress, cells recovered at 37 °C, and samples were harvested periodically, i.e., 1 h, 6 h, 12 h, and 24 h, in addition to the heat-stress completion time point of 0 h (Figure S2). This generated a 24-h stress response time course. We initially tested the models for stress induction through a specific time-point analysis. As an expected first consequence of the heat, we estimated protein aggregation in the maximally stressed cells (ET: 44 °C, ED: 2 h) immediately after heat stress, i.e., 0 h, and at 24 h post-heat stress (Figure 1a). Protein aggregation was detected immediately at 0 h, with the highest levels around 35% aggregation in differentiated SH-SY5Y cells, with Daoy (medulloblastoma) and SH-SY5Y (neuroblastoma) cells displaying almost similar levels of aggregation (10–15%). The protein aggregates in all three cell types resolve 24 h after heat exposure. In addition, utilizing the xCELLigence real-time cell analysis (RTCA) system, we evaluated the impact of heat stress on cell viability and proliferation. We monitored the cell behavior over 72 h in control cells grown at 37 °C and in the maximally stressed cells, i.e., cells experiencing stress conditions defined by ET: 44 °C, ED: 2 h (Figure 1b). In all three cell types, 2-hour exposure to 44 °C generates a measurable reduction in cell proliferation, which is observable even 24 h after heat stress. This reduced cellular proliferation alludes to persistent cellular stress, hence plausibly an active stress response.

### 3.2. Dose Responsive Heat-Stress Models

**Figure 2:**
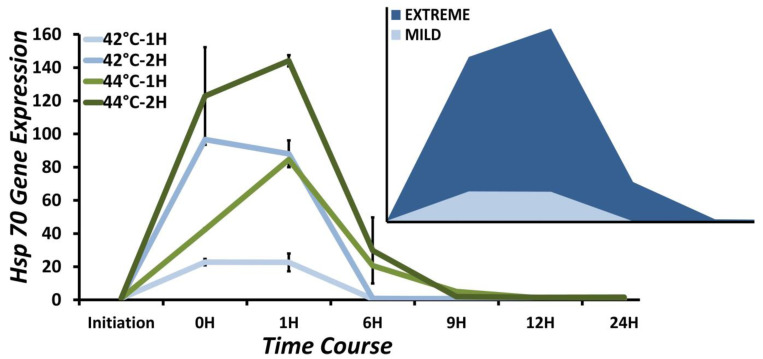
Dose-responsive nature of heat stress in SH-SY5Y cells. Time-course analysis of Hsp70 gene expression induction at 42 and 44 °C for 1 and 2 h. Initiation represents the time point when cells are transferred to heat-stress conditions. 0 h represents the start point of the recovery at 37 °C. As the magnitude of heat stress increases, the heat shock response proportionately goes up. The temperature has a slightly different impact on expression baseline attainment at 9 h at 44 °C versus (green) at 6 h at 42 °C (blue). Inset: Graphic representations of the extreme and mild heat stress models. (see Figure S3 for a detailed time course including the response at ET: 44 °C and ED: 1 and 2 h).

We generated comprehensive dose-responsive heat-stress models utilizing a combination of variable heat-stress exposure and a time-series approach for stress-response studies. Therefore, we evaluated the capacity of stress models reported in Section 3.1 to elicit a tangible and effective stress response through measurement of Hsp70 gene expression immediately after heat-stress completion and at 1, 6, 9, 12, and 24 h post1and 2 h heat-stress exposure at 39, 42, and 44 °C. We initially tested this approach with SH-SY5Y cells because they are precursors for generating differentiated neurons. The gene expression values for all tested conditions are tabulated in Table 1. Figure 2 represents the analysis of the time course of Hsp70 gene expression during the recovery phase after heat stress at 42 and 44 °C for 1 and 2 h. There are three visible blocks: dark green, representing the expression at ET: 44 °C, ED: 2 H; two in the middle in dark blue and light green, representing ET: 42 °C, ED: 2 H, and ET: 44 °C, ED: 1 H, respectively; and third in light blue, representing ET: 42 °C, ED: 1 H. The dose-responsive nature of the Hsp70 gene expression is obvious as the exposure temperature [ET] and duration of exposure [ED] increase. In the case of ET at 44 °C, Hsp70 gene expression levels reach the baseline 9 h after, while for ET at 42 °C, this is achieved only 6 h after stress exposure. Despite this, the expression of Hsp70 differs very marginally at ET: 44 °C, ED: 1 h and ET: 42 °C, ED: 2 h, while there is an approximately six-fold difference in expression at ET: 44 °C, ED: 2 h, and ET: 42 °C, ED: 1 h. Utilizing similar approaches in Daoy and differentiated SH-SY5Y cells, these two conditions were therefore classified as *extreme* and *mild* and formed the basis for further studies (Figure 2, Inset).

### 3.3. Chaperone Gene Expression during Heat-Shock Response

**Figure 3:**
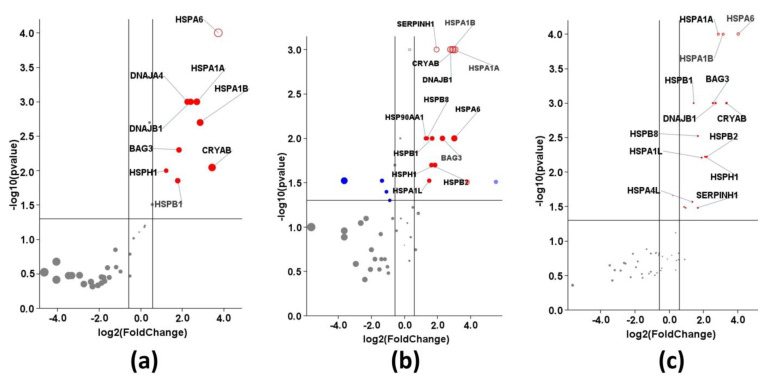
Volcano plots for (**a**) Daoy cells (**b**) SH-SY5Y cells (**c**) Differentiated SH-SY5Y cells: Log_2_ fold-changes in gene expression between 0 h (completion of heat stress exposure) and control are plotted against *t*-test *p*-values. A higher position indicates higher statistical significance, and a position farther from the central axis means larger gene expression changes. Some of the very significant genes are labeled.

We initially performed a time-dependent analysis of chaperone gene expression in an extreme heat stress model (ET: 44 °C, ED: 2 h) using the Human Heat Shock Proteins and Chaperones RT^2^ Profiler PCR Array (Qiagen). We report the gene expression immediately after heat stress, i.e., 0 h and 1 h, and 6 h in the recovery phase. Genes overexpressing at the log2 cut-off fold change of 1.5, and a statistically significant *p*-value < 0.05 were further analyzed. On this basis, twelve genes among heat-shock proteins and chaperones in medulloblastoma Daoy (Figure 3a), fourteen genes in neuroblastoma SH-SY5Y (Figure 3b), and sixteen genes in differentiated SH-SY5Y are overexpressed (Figure 3c). The eleven genes HSPA1A, HSPA1B, HSPA6, HSPA4L, HSPB1, HSPB8, CRYAB, HSPH1, DNAJB1, DNAJA4, and BAG3 are common to the three cell types. HSPA5 expression is specific to Daoy cells; HSPA1L, HSP90AA1, and SERPINH1 are specific to both SH-SY5Y and SH-SY5Y(D) cells; and HSPB6 and DNAJA1 are specific only to SH-SY5Y(D). Therefore, seventeen of the eighty-four genes from the Human Heat Shock Proteins and Chaperones RT^2^ Profiler PCR Array overexpress as part of the heat-induced proteotoxic stress response in these models of neuronal heat stress. Based on protein family or group distribution, these seventeen genes belong to eight groups (Table 2). The topmost hit is heat shock 70 kDa protein with six members, followed by four from small heat shock proteins, three from the DnaJ homolog family, and one each from heat shock protein 90 kDa alpha (cytosolic) class A, molecular chaperone regulator three of the BAG family, heat shock protein 105 kDa, and Serpin peptidase inhibitor, clade H (heat shock protein 47), member 1, (collagen binding protein 1).

### 3.4. Temporal Analysis of Gene Expression

**Figure 4:**
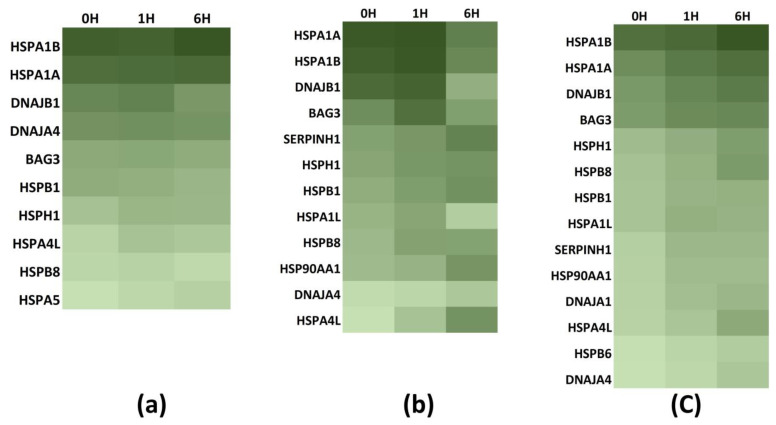
Heat maps showing the temporal expression of statistically significant (*p* < 0.05) expressed genes at the completion of heat stress (0 h) and during recovery at 37 °C at 1 h (1 h) and 6 h. The maximal time point for gene expression at 0–1 h in (**a**) Daoy, (**b**) SH-SY5Y, and 6 h in (**c**) differentiated SH-SY5Y. In all three cell types, significantly high expression of *HSPA1A*, *HSPA1B*, *DNAJB1*, and *BAG3* at 0 h is visible.

The extreme heat stress model (ET: 44 °C, ED: 2 h) has a temporal gene expression profile that includes conditions immediately at the start of exposure to heat stress (0 h) and 1 and 6 h after heat stress, which is during recovery at 37 °C. The ‘*maximal expression time point*’, the time at which the highest expression is attained, indicates the stress-response role. 0 h gene expression identifies genes induced near-simultaneously with stress initiation, while those expressed in later stages are more specific to post-stress recovery. A significant overexpression immediately at the stress onset, i.e., 0 h, indicates a potentially crucial impact on the stress response. In the case of medulloblastoma Daoy cells (Figure 4a) and neuroblastoma SH-SY5Y cells (Figure 4b), the maximum expression point in most overexpressed genes is between 0 and 1 h. In the case of differentiated SH-SY5Y cells, maximal expression is observed 6 h after stress (Figure 4c). But what is pertinent to note is that in these differentiated SH-SY5Y cells, 0 h and 1 h expression in all analyzed genes are also very high, and there are no significant differences in expression at 6 h post-stress.

The four top overexpressed genes common to all cell types include *HSPA1A* (6–8 log-fold increase), *HSPA1B* (7–8 log-fold increase), *DNAJB1* (5–7 log fold increase), and BAG3 (3–6 log-fold increase), the first two of which are the Hsp70 homologs. Furthermore, heat shock 70-kDa protein 6 (HSPA6) overexpresses, as is concluded from the low average threshold cycle (C_t_) values compared to the C_t_ value in the control samples. It is 16.5 and 16.3 in medulloblastoma Daoy cells, 25.6 and 26.1 in neuroblastoma SH-SY5Y cells, and 13.4 and 13.1 in differentiated SH-SY5Y cells, respectively, at 0 and 1 h. The exact fold change is impossible due to the controls’ unreliable Ct value (>30). As highlighted in Table 2, Hsp70 family proteins form the bulk of the proteins involved in stress response. Therefore, we included in our analysis the other two Hsp70 chaperones, HSPA4L and HSPA1L. HSPA4L overexpresses in all three cell types, whereas HSPA1L overexpresses only in SH-SY5Y and differentiated SH-SY5Y cells. HSPA4L overexpresses 2–4-fold, while HSPA1L varies between 3–4-fold. Therefore, five members of the Hsp70 family, HSPA1A, HSPA1B, HSPA6, HSPA1L, and HSPA4L, play a substantial role in the heat-induced proteotoxic stress response in these neuronal cell models.

The other two primary genes, DNAJB1 (5–8-fold) and BAG3 (4–7-fold) are from protein groups that regulate the function of Hsp70, particularly the ATPase reaction cycle and substrate recruitment [25]. DNAJA4 (1.5–5 log fold increase) is the other member protein expressed in the three cell types. HSPH1 (heat shock protein 105 kDa) with a 2.5–5 fold increase is also prominently overexpressed and acts as a co-chaperone for Hsp70s [12]. Thus, the expression of five Hsp70 members accompanies the overexpression of four co-chaperones/cofactors, viz DNAJB1, DNAJA4, BAG3, and HSPH1.

### 3.5. Protein Expression Profiling of Hsp70 Members

**Figure 5:**
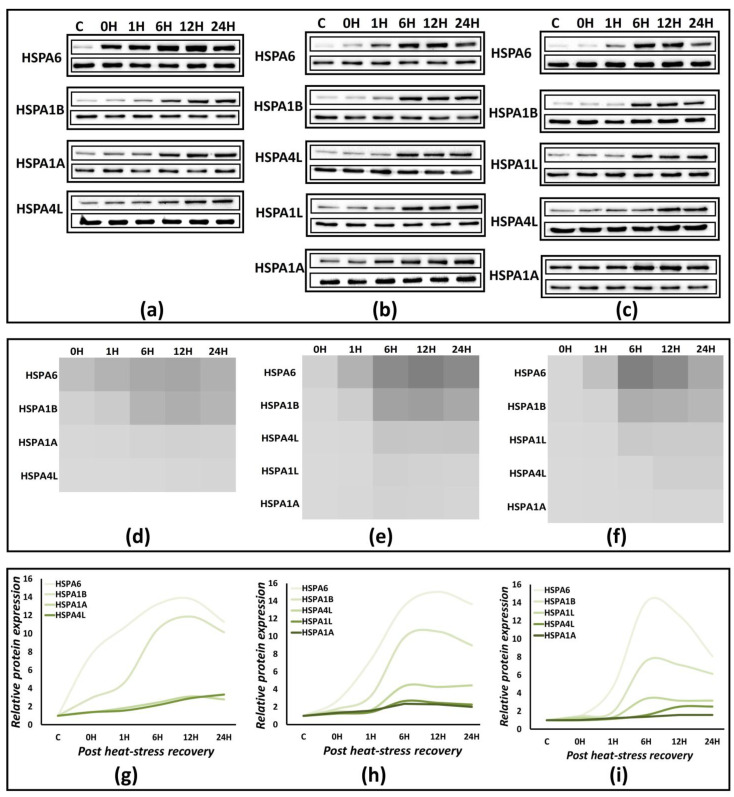
Protein profiling of the significantly expressing Hsp70s in Daoy (**a**), SH-SY5Y (**b**), and differentiated SH-SY5Y (**c**) cells. The lanes are in order Control, 0 H: immediately after heat stress, followed by periodic points during recovery at 37 °C at 1 h, 6 h, 12 h, and 24 h. *Heatmaps* (**d**–**f**) represent the relative expression of each protein with respect to the control (37 °C) expression normalized against GAPDH. They also highlight differential protein expression with respect to the other members. *Smoothened scatterplots* (**g**–**i**) trace the relative protein expression up to 24 h post heat stress and highlight the ‘maximal protein expression’ time point. (Figure S4: Bar graphs for quantification and statistical significance. Figure S5: Western blot quantification time plots and fold-change plots corresponding to scatter plots (**g**–**i**)).

We analyzed the protein expression for the extreme heat stress model (ET: 44 °C, ED: 2 h) of significantly expressing Hsp70s, HSPA1A, HSPA1B, HSPA6, HSPA1L, and HSPA4L utilizing a time-series approach analyzing the expression immediately after heat-stress exposure, i.e., at 0, 1, 6, 9, 12, and 24 h during recovery at 37 °C (Figure 5a–c). These points ensure coverage of important aspects of the stress response pathway: 0 h: stress completion; 1 h: gene-expression critical point; 6 h: protein-expression critical point; and 24 h: significant protein-expression reduction. Some visibly common features of Hsp70’s protein expression exist in these cells. HSPA6 and HSPA1B are two very highly expressed proteins and feature prominently in protein expression heatmaps (Figure 5d–f). The fold change in *HSPA6* expression varies between 13–14 at its maximum, and there is minimal variation between the three cell types (Figure S2a–c). HSPA1B overexpression varies between 7–11-fold (Figure S2d–f), with the least of three in differentiated SH-SY5Y cells. HSPA1L specifically overexpresses only in neuroblastoma SH-SY5Y and differentiates SH-SY5Y cells at 2.5–3.5-fold, with a relatively higher value in the case of later (Figure S2g,h). HSPA4L expression varies between 2–4-fold, with 2.5-fold expression in differentiated SH-SY5Y cells (Figure S2i–k). The overexpression of housekeeping Hsp70, HSPA1A, is the least among the five overexpressed Hsp70s, with a 1.6-fold change in the differentiated SH-SY5Y cells (Figure S2l–n).

The phase between 6–12 h post-recovery at 37 °C is significant from the protein-expression point of view for all the proteins in the three cell types. Thus, 6–12 h is the ‘*maximal protein-expression point*’, where statistically significant maximal protein expression exists (Figure 5g–i and Figure S2). The protein expression starts receding after this point, but not significantly, and even at 24 h post-heat stress, it is prominently visible in the case of HSPA6 and HSPA1B (Figure 5g–i), where the protein expression fold-change ranges between 8–11 and 6–10 for HSPA6 and HSPA1B, respectively. Thus, the heat-induced proteotoxic stress response in these neuronal cell models is substantially and significantly active still 24 h post heat stress, with a substantial contribution from HSPA6 and HSPA1B.

### 3.6. Extreme and Mild Heat Stress-Response Comparison

#### 3.6.1. Gene Expression Analysis

**Figure 6:**
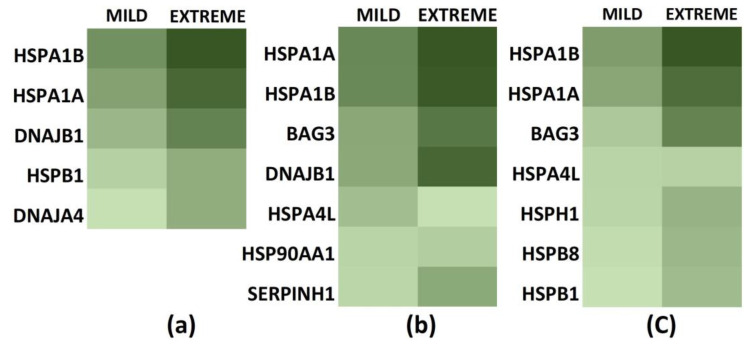
Heat maps showing relative gene expression profiles of stress responses during mild and extreme heat-stress exposure. (**a**) Daoy, (**b**) SH-SY5Y, and (**c**) differentiated SH-SY5Y cells. Comparisons were made for genes significantly expressed during mild heat stress.

Utilizing the same Human Heat Shock Proteins and Chaperones RT^2^ Profiler PCR Array, we compared the gene expression in extreme and mild heat stress around maximal gene expression, i.e., 1 h post heat stress (Section 3.4). 05 genes in Daoy cells (Figure 6a), 07 genes in SH-SY5Y cells (Figure 6b), and 07 genes in differentiated SH-SY5Y cells (Figure 6c) are overexpressed significantly during mild heat-stress exposure as compared to 12, 14, and 16 genes, respectively, during extreme conditions. Comparing their expression fold-change with extreme-stress expression shows explicit dose-dependent behavior in most of the genes (Figure 6). HSPA1A varies between 4–5-fold for Daoy and SH-SY5Y cells and around 9-fold for differentiated SH-SY5Y cells. HSPA1B changes 4–5-fold for medulloblastoma Daoy and neuroblastoma SH-SY5Y and around 13-fold for differentiated SH-SY5Y cells. For HSPA6, fold-change comparisons are impossible (Section 3.4). Still, the comparison of threshold cycle (C_t_) values shows a similar trend with mild stress values between those of control and extreme heat stress. Differentiated SH-SY5Y show a more significant variation (C: 30.5, EX: 14, MI: 25) as compared to undifferentiated SH-SY5Y (C: 34, EX: 26, MI: 29). Thus, three Hsp70 members, HSPA6, HSPA1B, and HSPA1A, vary extensively between extreme and mild conditions, with slight variation in HSPA4L expression and no HSPA1L expression under mild stress conditions.

Among the Hsp70 function complementing proteins, BAG3 overexpresses 4-fold less in the case of neuroblastoma SH-SY5Y and 13-fold more in differentiated SH-SY5Y as compared to extreme stress. HSPH1 is another group member significantly expressed in differentiated SH-SY5Y cells, although around 3.5 times less than under extreme heat stress.

#### 3.6.2. Protein Expression Analysis

**Figure 7:**
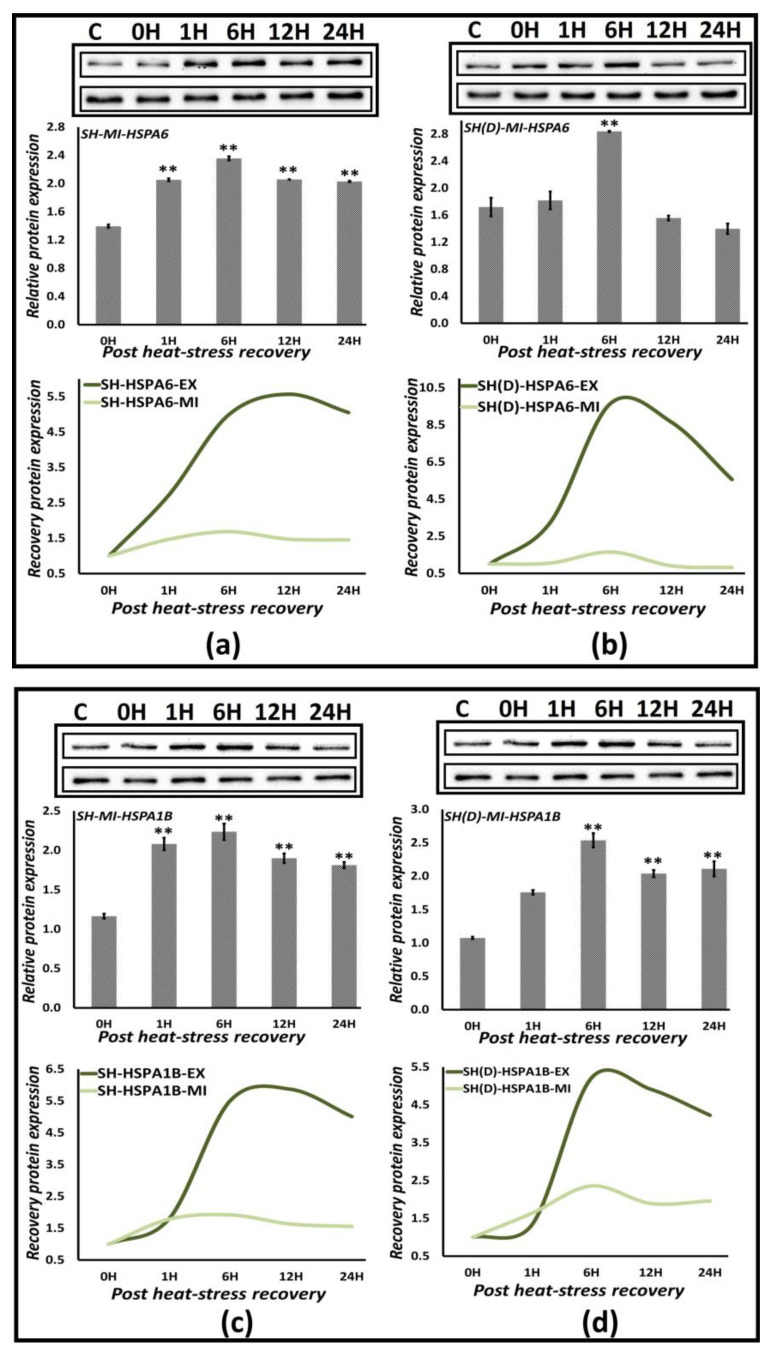
Protein expression during extreme and mild heat stress. (**a**,**b**) HSPA6; (**c**,**d**) HSPA1B in SH-SY5Y [SH] and differentiated SH-SY5Y [SH(D)] cells. *The bar graphs* represent the quantification of the expression levels compared to the GAPDH control. The unpaired t-test calculated *p* values, ** *p* < 0.05. *Smoothened scatterplot* to trace protein expression fold change with respect to expression at 0 h. Normalizing to 0 h highlights the net protein expression during the recovery phase. The lanes are in order Control, 0 h: immediately after heat stress followed by periodic points during recovery at 37 °C at 1, 6, 12, and 24 h.

We analyzed the protein expression of HSPA1A, HSPA1B, HSPA6, HSPA4L, and HSPA1L utilizing the same time-series approach as for extreme heat-stress analyzing the expression immediately after heat-stress exposure, i.e., at 0, 1, 6, 9, 12, and 24 h during recovery at 37 °C (Figure 7 and Figure S6). Keeping in mind the more representative nature of differentiated SH-SY5Y cells and the significant harmony in the gene- and protein-expression analysis so far, we, therefore, analyzed the mild heat-stress protein expression in neuroblastoma SH-SY5Y and differentiated SH-SY5Y cells only, irrespective of the observation in gene expression analysis. Only two proteins, HSPA6 (Figure 3a,b) and HSPA1B (Figure 3c,d), are significantly overexpressed in both cell types, while HSPA4L is overexpressed only in SH-SY5Y cells (Figure S3f). Although HSAP1A (Figure S3a,b) and HSPA1L (Figure S3c,d) protein overexpression are also visible under mild conditions, it is not at significant levels. HSPA6 and HSPA1B fold expression vary between 2–3 in mild conditions, while they are 13–14-fold and 7–11-fold, respectively, in extreme stress conditions. We further evaluated these differences by calculating the ‘recovery expression fold change’ to the 0 H fold change. This recovery-expression fold change varies between 1.5–2.5 during mild stress, not a significant change from the 2–3-fold observed as compared to control (Figure 7, Scatter Plots), pointing towards a regulated but significant role for HSPA6 and HSPA1B in mild stress conditions.

### 3.7. Expression Profiling of Hsp70 Co-Expressing Proteins

**Figure 8:**
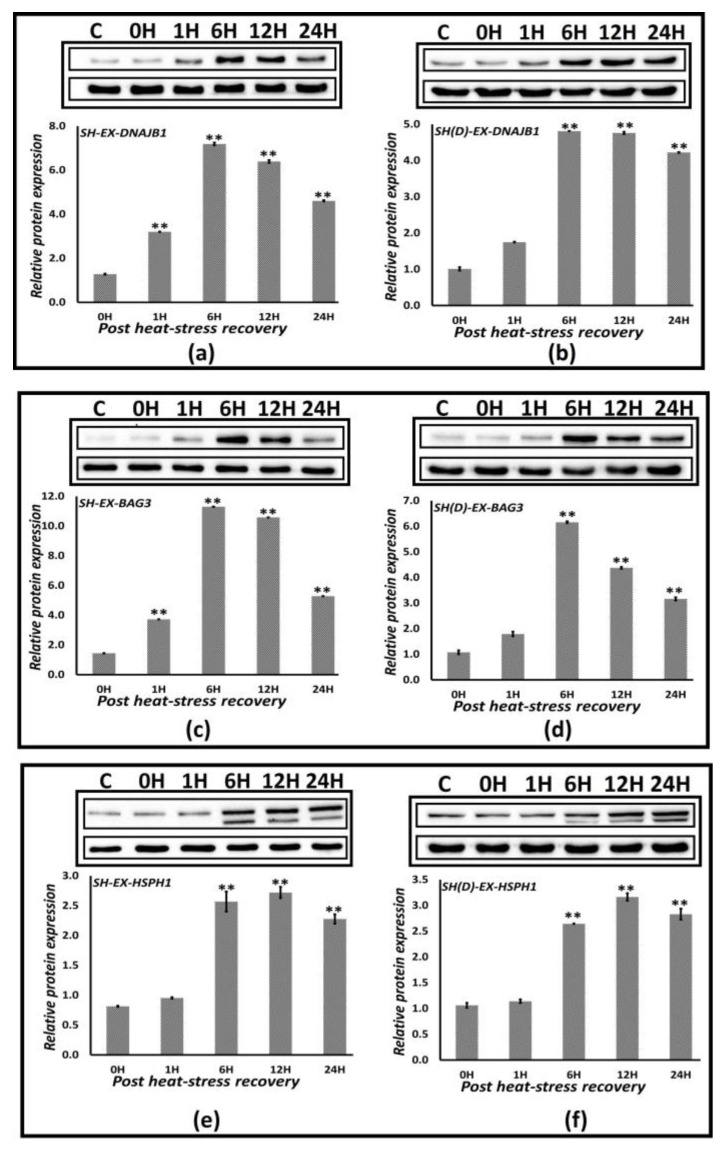
Protein profiling of the significantly Hsp70 co-expressing proteins. (**a**,**b**) DNAJB1, (**c**,**d**) BAG3, and (**e**,**f**) HSPH1 in SH-SY5Y [SH] and differentiated SH-SY5Y [SH(D)] cells. *The bar graphs* represent the quantification of the expression levels compared to the GAPDH control. The unpaired t-test calculated *p* values, ** *p* < 0.05.

Utilizing the time-series approach applied for Hsp70 protein expression profiling and evaluating the expression immediately after heat-stress exposure, i.e., at 0, 1, 6, 9, 12, and 24 h during recovery at 37 °C, we analyzed the protein expression of three highly transcribed non-Hsp70 proteins: DNAJB1, BAG3, and HSPH1 (Section 3.4). We performed this analysis only in SH-SY5Y and differentiated SH-SY5Y cells because there is high resonance at the gene- and protein-expression level with Daoy cells, and also because differentiated SH-SY5Y cells are more characteristic of the human neurons. As is the case for Hsp70 proteins, a time-bound response is noticeable in their protein expression, accompanied by a significant reduction at 24 h post-heat stress (Figure 8). DNAJB1 overexpresses 7- and 5-fold for neuroblastoma SH-SY5Y [SH] and differentiated SH-SY5Y [SH(D)], respectively; BAG3 at 11- and 6-fold; and HSPH1 at 2.7- and 3.2-fold. For HSPH1, both isoforms, the dual constitutive and stress-inducible α-isoform and the strictly stress-inducible β-isoform, are seen (Figure 8e,f) [26].

### 3.8. Co-Expression Network Analysis

**Figure 9:**
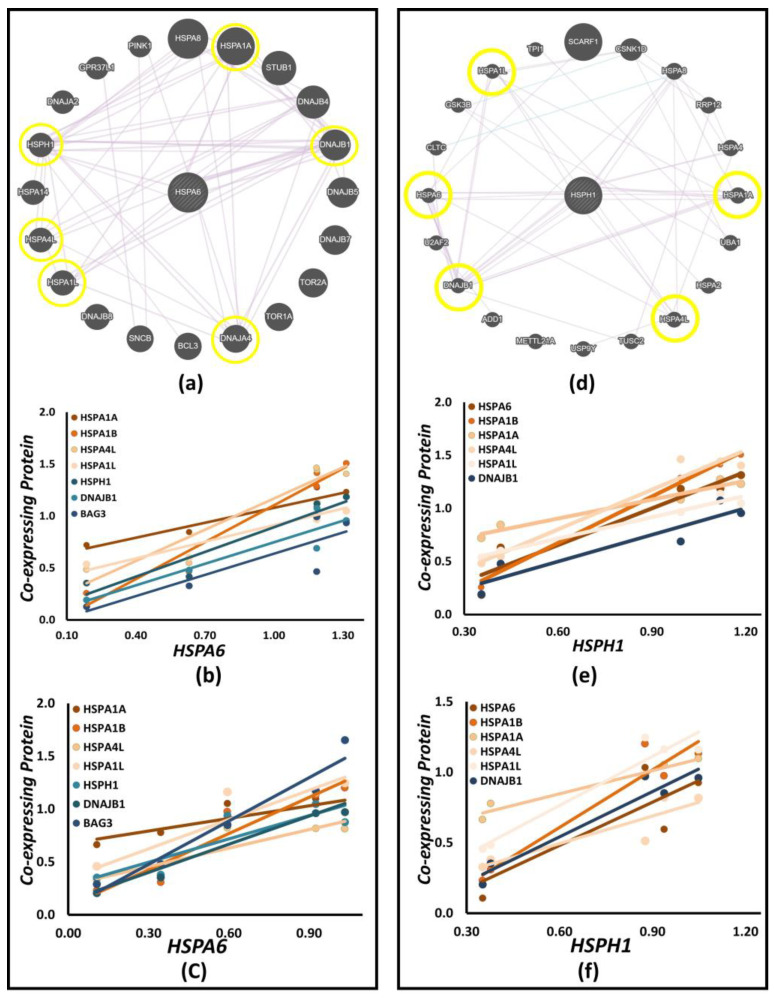
Network Analysis. GeneMANIA diagrams show direct and indirect co-expressed interactions of HSPA6 (**a**) and HSPH1 (**d**). The straight lines connecting the genes indicate all potential co-expressions, and the circled genes exist in the present study. Co-expression plots for HSPA6 (**b**,**c**) and HSPH1 (**e**,**f**) in SH-SY5Y and differentiated SH-SY5Y cells, respectively. Highly significant protein level co-expression is observed with r ≥ 0.95 in almost all the analyzed co-expressions for HSPA6 and HSPH1. (Table S2: Pearson correlation coefficients (*r*) and *p*-values for the co-expression analysis of HSPA6 and HSPH1. Figure S7: GeneMANIA diagram showing co-expressed interactions of HSPA1A, HSPA1B, HSPA4L, and HSPA1L).

Cooperative interactions with function-modulating proteins and other chaperone systems enhance the functional diversity of Hsp70. Therefore, we analyzed the interactions among the differentially expressed genes based on their co-expression patterns. GeneMANIA predicted the co-expression-based interactions for each overexpressed gene, and we manually curated the direct or pairwise interactions for individual genes (Table 3, Column 2). Following this, we used the RT2 Profiler data (Section 3.3) to analyze co-expression patterns in the three cell types (Table 3, Columns 3, 4, and 5). HSPA6 and HSPH1 co-express with most of the genes, with HSPA6 co-expressing in a pairwise manner with HSPA1A, HSPA4L, HSPA1L, HSPH1, DNAJB1, and DNAJA4 (Figure 9a) and HSPH1 with HSPA1A, HSPA6, HSPA4L, HSPA1L, and DNAJB1 (Figure 9d). Co-expression of HSPA1B with HSPA6 and HSPH1 is only seen when HSPA1B is used as an input (Figure S4). We calculated the pairwise correlation coefficients for HSPA6 and HSPH1 co-expression in neuroblastoma SH-SY5Y and differentiated SH-SY5Y cells (Table S2). A highly significant co-expression of proteins is observed with a Pearson correlation coefficient (*r*) ≥ 0.95 in these interactions, which is very well reflected in the linear nature of the plots for both HSPA6 (Figure 9b,c) and HSPH1 (Figure 9e,f). Thus, the Hsp70 member proteins in these human neuronal cell models display strong co-expression behavior among themselves and with various known function-modulating factors.

## 4. Discussion

In addition to other detrimental cellular and molecular outcomes, hyperthermia-induced damage leads to protein misfolding and subsequent aggregation. Proteins are sensitive to temperature because they can only work in a narrow range of temperatures. Therefore, in addition to their sensitivity to thermal fluctuations, stressors such as reactive oxygen and nitrogen species and heavy metals affect protein stability [6,27,28]. Misfolded proteins and their aggregated products are neurotoxic by nature and a trigger for many neurodegenerative disorders [7]. Heat stroke is a life-threatening manifestation among heat-related illnesses, with the long-term implication of persistent neurological damage. What differentiates heat-induced neurological damage from other neurological diseases is the higher rate of aggregate accumulation and the dose-responsive nature of hyperthermia-induced neurological damage [5,15,16]. We generated cellular heat-induced dose-responsive proteotoxic stress models of human neurons using combinations of *magnitude*, defined by exposure temperature [ET], and *duration*, defined by exposure duration [ED]. These cells are medulloblastoma [Daoy], neuroblastoma [SH-SY5Y], and differentiated SH-SY5Y neuronal cells, potentially representing different neuron populations. 30% proteome aggregation in differentiated cells compared to medulloblastoma and neuroblastoma cells suggests a differential neuronal vulnerability to heat (Section 3.1). Cell-proliferation analysis and time-series Hsp70 gene expression quantification confirm an active stress response at 24 h post-stress. Although stress response is transient, it ensures efficient cell recovery; consequently, there is tight control over the magnitude and duration of the stress response, which are proportional to the severity of the stress itself [29]. Therefore, the phases of the stress-response pathway correlate with the cell-fate outcomes. Considering this and the dose-responsive nature of thermal neuronal damage, we believe these stress models provide an excellent platform for studying heat-induced proteotoxic stress.

Cellular stress adaptation regulation occurs through gene expression modulation. In addition to their housekeeping functions, Hsp70 chaperones are the key players in the stress response, with their functional spectrum encompassing everything from folding to degradation [9]. We record the stress-induced overexpression of five members of the Hsp70 chaperone family: HSPA1A, HSPA1B, HSPA6, HSPA1L, and HSPA4L. Time series monitoring of gene and protein expression points to ‘maximal gene expression points’ around 1 h (Section 3.4) and 6 h (Section 3.5), respectively. Chaperones are among the first heat shock response proteins synthesized and are related to their demand during protein refolding and proteostasis modulation processes [6]. Therefore, this ‘maximal gene expression point’ around 1 h resonates with the idea that the regulation of stress response gene expression is primarily a transcriptional phenomenon [30,31]. Although the ‘maximal protein expression point’ is 5 h after gene expression, protein overexpression is observed immediately after heat stress, i.e., 0 h (Figure 5). Maximum protein aggregation at 0 h, despite the presence of chaperone overexpression at this point, means a tilt of homeostasis toward the denaturing process and a further requirement for the repair process. HSF1 regulates the chaperone transcription activation and exists as an inactive monomer complex with Hsp70 [32] and Hsp90 [33]. In the chaperone titration model, these chaperones are titrated away by non-native proteins, allowing HSF1 to trimerize to induce transcription of the heat shock protein gene [34,35]. The homeostasis reestablishes itself once the proteotoxic stress ceases and sufficient free chaperone capacity is reinstituted, which sets HSF1 free to bind to its partners, viz., Hsp70 and Hsp90, to rebind [36]. Despite this, there is a lag between transcription induction and translation initiation because RNA processing requires some time along with the translation, hence the poor initial mRNA–protein correlations [37]. However, mechanisms such as mRNA quality control bypass the demand for stress response proteins. This ensures minimal lag between transcription and translation [38]. Hsp70 proteins specifically have a self-mRNA stabilization ability observed during heat shock [39]. Overall, the expression profile of Hsp70s here corresponds with other systems such as Drosophila [40], *C. elegans* [41], and humans [42].

**Figure 10:**
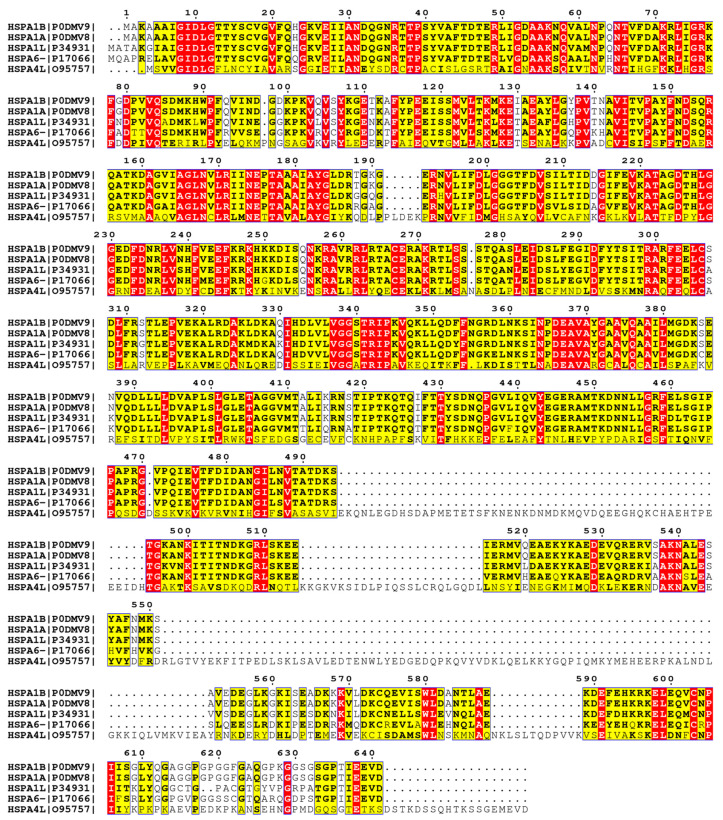
Multiple Sequence Alignment (MSA) of Hsp70 overexpressed members HSPA1B, HSPA1A, HSPA1B, HSPA1L, HSPA6 and HSPA4L. The red regions highlight the high-identity regions. The sequences are the UniProt sequences, with Uniprot IDs listed alongside the label (see Figure S5. MSA for highly homologous HSPA1A, HSPA1B, HSPA4L, and HSPA1L).

The five Hsp70 chaperones, HSPA1A, HSPA1B, HSPA6, HSPA1L, and HSPA4L, overexpressed in human neuronal heat-induced proteotoxic stress models belong to a large family of Hsp70 homologs, with expression levels regulated according to cellular requirements in a compartment-specific manner [9,43]. An important differentiating characteristic of Hsp70s is the difference in the amino acid sequence, which has functional implications, especially for interactions with other proteins, consequently expanding the functional landscape. The amino acid sequences of the five Hsp70s found here are also different (Figure 10). HSPA4L has the most different amino acid sequences, while HSPA1A, HSPA1B, HSA1L, and HSPA6 are very similar to each other (Figure S8). HSPA1A and HSPA1B are among the most conserved, are essential to the chaperone machinery of housekeeping, and also form an equally crucial element of the stress response pathways [43,44]. Despite this high HSPA1A and HSPA1B homology, variable stress response expression is visible (Figure 5), with overall HSPA1A protein overexpression of 2–3-fold compared to 10-fold in the case of HSPA1B. This variation is attributed to the genetic and structural diversity in the HSPA1A and HSPA1B gene loci, leading to differentiating traits, including differential transcriptional complex recruitment [44]. Interestingly, the other overexpressed Hsp70 here, HSPA1L, is part of the same 14kb region of chromosome 6p21.2 on which HSPA1A and HSPA1B occur, although in reverse orientation [44]. HSPA1L protein expression is identified only in SH-SY5Y and differentiated SH-SY5Y cells at 2.5–3.5-fold, with relatively higher levels in the case of the latter (Figure 5; Figure S2g,h). Although HSPA1L’s importance in cancer proteostasis is coming to the fore [45], HSPA1L is otherwise primarily a testes-specific chaperone [46]. Therefore, the HSPA1L overexpression, especially higher expression in neuron-differentiated SH-SY5Y cells, points toward a neuron-specific role in overall stress proteostasis or specific heat-induced proteotoxic stress. The other highly overexpressed chaperone, HSPA6, despite not sharing the genomic loci with HSPA1A, HSPA1B, and HSPA1L, shares a very high protein identity (80%). The differentiating roles of HSPA6 relative to other Hsp70s are well analyzed [21,47], but our results highlight the predominance of HSPA6 in heat-induced proteotoxic stress responses in neuronal cell models. This information has implications for further exploration of neuron-specific proteotoxic stress pathways and chaperone-based therapeutic approaches.

Although Hsp70 chaperones perform many functions in the protein life cycle, the fundamental universal activity is ATP-dependent substrate protein-binding. Consequently, the functional diversity of Hsp70 chaperones results from their interaction with other function-modulating proteins. We identified three such overexpressed proteins, DNAJB1, BAG3, and HSPH1, in our analysis (Section 3.7). DNAJB1 is a member of a group of proteins belonging to a family called J-proteins, while BAG3 and HSPH1 work as nucleotide exchange factors (NEFs). Members of both protein families regulate the Hsp70s ATPase reaction cycle by working in substrate recruitment [25,48]. The defining feature of J-proteins is a highly conserved ~70 amino acid signature region called the J domain, with around 40 members in humans [13]. DNAJ proteins have neuroprotective functions [49,50],and DNAJB1 specifically reduces neurotoxicity [51]. More studies are required to decipher the role of DNAJB1 in response to heat-induced proteotoxic stress. BAG3 is a member of the Bag-type nucleotide exchange factors (NEFs) characterized by an ~85 amino acid Hsp70 ATPase interacting domain and is the only known stress-induced member [52,53,54]. HSPH1, also known as Hsp110, is a member of Hsp70-like NEFs, named for their similarity with Hsp70 chaperones. Since the ATPase domain of Hsp110 proteins is expected to possess some chaperone activity [55]. HSPH1 is a stress-inducible HSP110 postulated to store partially denatured proteins under stress with subsequent facilitation of Hsp70 activity through their NEF activity [13]. The chaperone interactions do not essentially entail physical interactions, but a mutual affinity drives the chaperone network for similar clients, such as heat-induced misfolded proteins or aggregates. A strong co-expression correlation (Section 3.8) between the chaperones, co-chaperones, and nucleotide exchange factors identified in this study signifies their critical importance in the heat-induced proteotoxic stress in these neuronal cell models.

Considering the functional significance of these Hsp70 chaperones in the proteotoxic stress response, their targeting to reverse and combat protein aggregation-mediated neurodegeneration is potentially an excellent therapeutic approach. Hsp70 up-regulation and modulation are widely tested in treating many neurodegenerative diseases [18,19,56,57]. Chaperones, in combination, are more potent in eliciting preventive responses than individual entities [58]. Among the Hsp70s identified here, HSPA4L is the most diverse, with 25% sequence homology with the rest of the overexpressing members (Figure 10). Considering the diversity in the functional landscape, even in very close homologs [21], targeted therapeutic strategies will require considering these facts. The heat-induced proteotoxic stress potentially causes cellular damage through multiple mechanisms, such as the direct inactivation of essential proteins, the overpowering of protein degradation pathways, or the direct toxic effects of misfolded and aggregated protein accumulation. Thus, full-blown damage means extreme homeostasis caused by the overpowering impact of negative factors. Therefore, targeted therapies require knowledge of the type and magnitude of damage and the corresponding stress response factors because the two are intricately linked [29]. These variable responsive differences also exist in our analysis of mild heat stress (Section 3.6.2), where only HSPA6 and HSPA1B are significantly overexpressed and at reduced levels. Thus, Hsp70 chaperone-based therapeutic approaches require a thorough understanding of their individual and collective roles in stress response. This study, therefore, goes a long way in helping expand knowledge about various aspects of their heat-induced stress response and lays a platform for exploitation in further translational studies.

## 5. Conclusions

Hyperthermia-induced central nervous system (CNS) dysfunction is among the severe heat-related illnesses. Heat stress leads to protein misfolding and nonspecific aggregation, a known cause of neurodegenerative disorders. Depending upon stress severity, heat-induced proteotoxic stress causes cellular damage through numerous mechanisms, such as direct inactivation of critical proteins, overpowering protein degradation pathways, or direct toxic effects of protein aggregates. The proteostasis, particularly chaperones like Hsp70s, play roles in housekeeping and stress response. Heat-induced neuronal damage includes higher aggregate accumulation, dose-responsive injury, and persistent damage. Therefore, heat-induced neurological damage studies need a separate treatment from other neurological disorders.

Consequently, we performed this study under two conditions categorized as “extreme” and “mild” heat stress with stress response analysis in a time-series manner over a 24-hour duration. We identified the differential and relative expression of five Hsp70 chaperones and four of their interacting partners. The co-expression of the highly overexpressed Hsp70 proteins and their interacting partners is significantly correlated. This represents a highly comprehensive map of the Hsp70 chaperone network in human neurons’ heat-induced proteotoxic stress models. We believe this outcome sheds significant and new light on the role of these proteins and goes a long way in contributing to the development of targeted therapeutic strategies.

## Figures and Tables

**Table 1:** Heat-stress model of SH-SY5Y. The Hsp70 gene expression fold-change values were calculated from six circumstances involving a combination of three exposure temperatures (ET) and two exposure durations (ED).

Exposure Temperature [ET]	Exposure Duration [ED]	Hsp70 Induction
39 °C	1 h	5.5 ± 0.7
	2 h	3.6 ± 1.1
42 °C	1 h	25.1 ± 7.7
	2 h	96.6 ± 1.3
44 °C	1 h	81.7 ± 7.4
	2 h	144.34 ± 4.38

**Table 2:** Classification of overexpressed genes according to Heat Shock Protein or Chaperone Family.

Chaperone Family	Overexpressed Genes	Proportion
Small Heat Shock Protein	HSPB1, HSPB8, CRYAB, HSPB6	4/8
**Heat shock 70 kDa protein**	**HSPA1A, HSPA1B, HSPA1L, HSPA4L, HSPA5, HSPA6**	**6/11**
Heat shock protein 90 kDa alpha (cytosolic), class A	HSP90AA1	1/1
Heat shock protein 105 kDa	HSPH1	1/1
DnaJ homolog subfamily A	DNAJA1, DNAJA4	2/4
DnaJ homolog subfamily B	DNAJB1	1/11
BAG family molecular chaperone regulator 3	BAG3	1/1
Serpin peptidase inhibitor, clade H (heat shock protein 47), member 1, (collagen binding protein 1)	SERPINH1	1/1

**Table 3:** Network analysis. GeneMANIA predicted co-expressed interactors of DEGs from the Human Heat Shock Proteins and Chaperones PCR Array and interactors specific to each cell type.

Gene	Predicted Co-Expressing Interactors	Daoy	SH-SY5Y	SH-SY5Y(D)
HSPA1A	HSPB1, HSPA6, HSPA8, HSPH1, BAG3	HSPA6, HSPH1, BAG3, HSPB1	HSPA6, HSPH1, BAG3, HSPB1	HSPA6, HSPH1, BAG3, HSPB1
HSPA1B	HSPA1A, HSPH1, HSPA6, HSPA5, HYOU1	HSPA1A, HSPA6, HSPH1	HSPA1A, HSPA6, HSPH1	HSPA1A, HSPA6, HSPH1
HSPA6	HSPA1A, DNAJB4, DNAJB1, DNDAJA4, DNAJB8, HSPA1L, HSPA4L, HSPH1	HSPA1A, HSPA4L, HSPH1, DNAJB1, DNAJA4	HSPA1A, HSPA1L, HSPA4L, HSPH1, DNAJB1, DNDAJA4	HSPA1A, HSPA1L, HSPA4L, HSPH1, DNAJB1, DNDAJA4
HSPA4L	HSPA8, RTN4, HSPH1, HSPA4, HSPA6, LUC7L2	HSPA6, HSPH1	HSPA6, HSPH1	HSPA6, HSPH1
HSPA1L	TOR2A, TPR1A, PRKN, SNCB, STUB1, HSPA6, HSPA4L, HSPH1	HSPA6, HSPA4L, HSPH1	HSPA6, HSPA4L, HSPH1	HSPA6, HSPA4L, HSPH1
HSPA4	HSPH1, BAG3, HSPA4L	HSPA4L, HSPH1, BAG3	HSPA4L, HSPH1, BAG3	HSPA4L, HSPH1, BAG3
BAG3	HSPB8, BAG5, HSPA8, HSPA4, HSPA1A, SYNPO2, ETFA, HSP90AB1, GORASP2, CAPZA1, DNAJB4	HSPA1A	HSPA1A, DNAJB4, HSPB8	HSPA1A, DNAJB4, HSPB8
DNAJA4	HSPA6, HSPE, HSPA1A, DNAJB4, DNAJB1, HSPA8, DNAJB11	HSPA1A, HSPA6, DNAJB1	HSPA1A, HSPA6, DNAJB1	HSPA1A, HSPA6, DNAJB1
DNAJB1	HSPA6, HSPA8, HSPH1, TARDBP, NUDC, DNAJB4, DNAJA1, HSPA1A	HSPA1A, HSPA6, HSPH1	HSPA1A, HSPA6, HSPH1, DNAJA1	HSPA1A, HSPA6, HSPH1, DNAJA1
HSPH1	HSPA8, HSPA4, HSPA1A, HSPA4L, DNAJB1, HSPA6, GSK3B, HSPA1L	HSPA1A, HSPA6, HSPA4L, DNAJB1	HSPA1A, HSPA6, HSPA4L, HSPA1L, DNAJB1	HSPA1A, HSPA6, HSPA4L, HSPA1L, DNAJB1
DNAJA1	TPP1, DNMA1, DNAJB1, HSPA9, HSPH1, HSPA6	HSPA6, HSPH1, DNAJB1	HSPA6, HSPH1, DNAJB1	HSPA6, HSPH1, DNAJB1

## Data Availability

No new data generated.

## Supplementary Materials

The following supporting information can be downloaded at: https://www.mdpi.com/article/10.3390/biology12030416/s1, Figure S1: Retinoic acid differentiation of SH-SY5Y cells into terminally differentiated human neurons, Figure S2: Cell culture and heat-stress experiments in Top Panel: Daoy Cells, Middle Panel: SH-SY5Y Cell, Lower Panel: Differentiated SH-SY5Y Cells, Figure S3: Dose-responsive nature of heat stress in SH-SY5Y cells, Figure S4: Western blot analysis of Hsp70 members, Figure S5: Western blot quantification time plots of Hsp70, Figure S6: Western blot images and quantifications of Hsp70 members in mild heat-stress conditions in SH-SY5Y and differentiated SH-SY5Y cells, Figure S7: Network analysis, Figure S8: Multiple Sequence Alignment among Hsp70 members HSPA1A, HSPA1B, HSPA4L, and HSPA1L, Table S1: Gene List for Human Heat Shock Proteins & Chaperones RT² Profiler PCR Array (Qiagen) allows for simultaneous expression analysis of 84 genes from the HSP90 Family (81 to 99 kDa), HSP70 Family (65 to 80 kDa), HSP60 Family (55 to 64 kDa), HSP40 Family (35 to 54 kDa), small HSPs (<34 kDa), and Chaperone cofactors, Table S2: Correlation coefficients (r) and p-values for the co-expression analysis of HSPA6 and HSPH1 in SH-SY5Y cells and differentiated SH-SY5Y cells, File S1: Original western blot images.
