# Supplementary material for: Profiling the Hsp70 Chaperone Network in Heat-Induced Proteotoxic Stress Models of Human Neurons
Source: Biology (Basel). 2023 Mar 9;12(3):416. doi: 10.3390/biology12030416 (PMC10045125; doi:10.3390/biology12030416)

HSPA6-12% gel

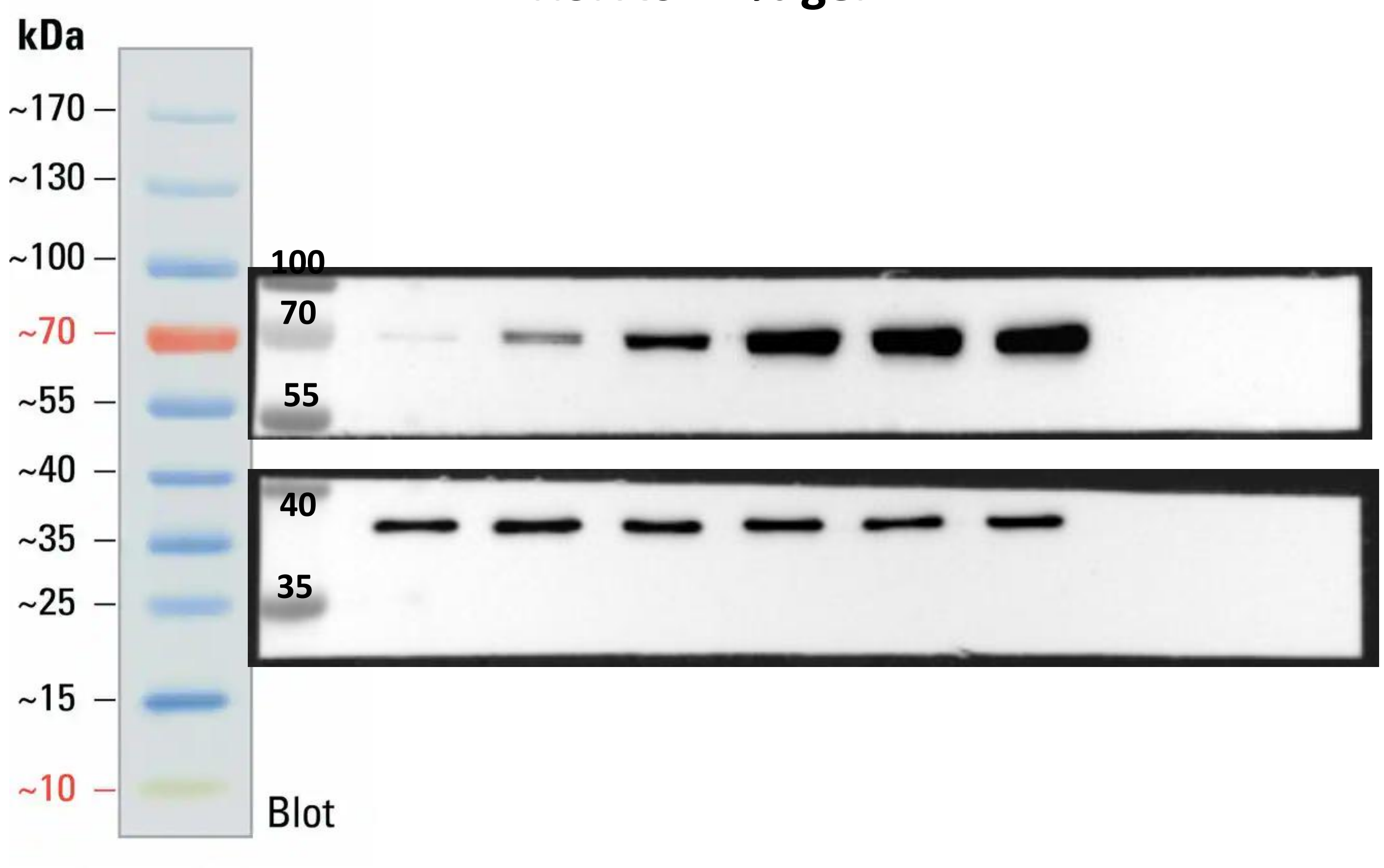

MW Marker: PageRuler™ Prestained Protein Ladder, 10 to 180 kDa ThermoFisher 26616

HSPA1B-12% gel

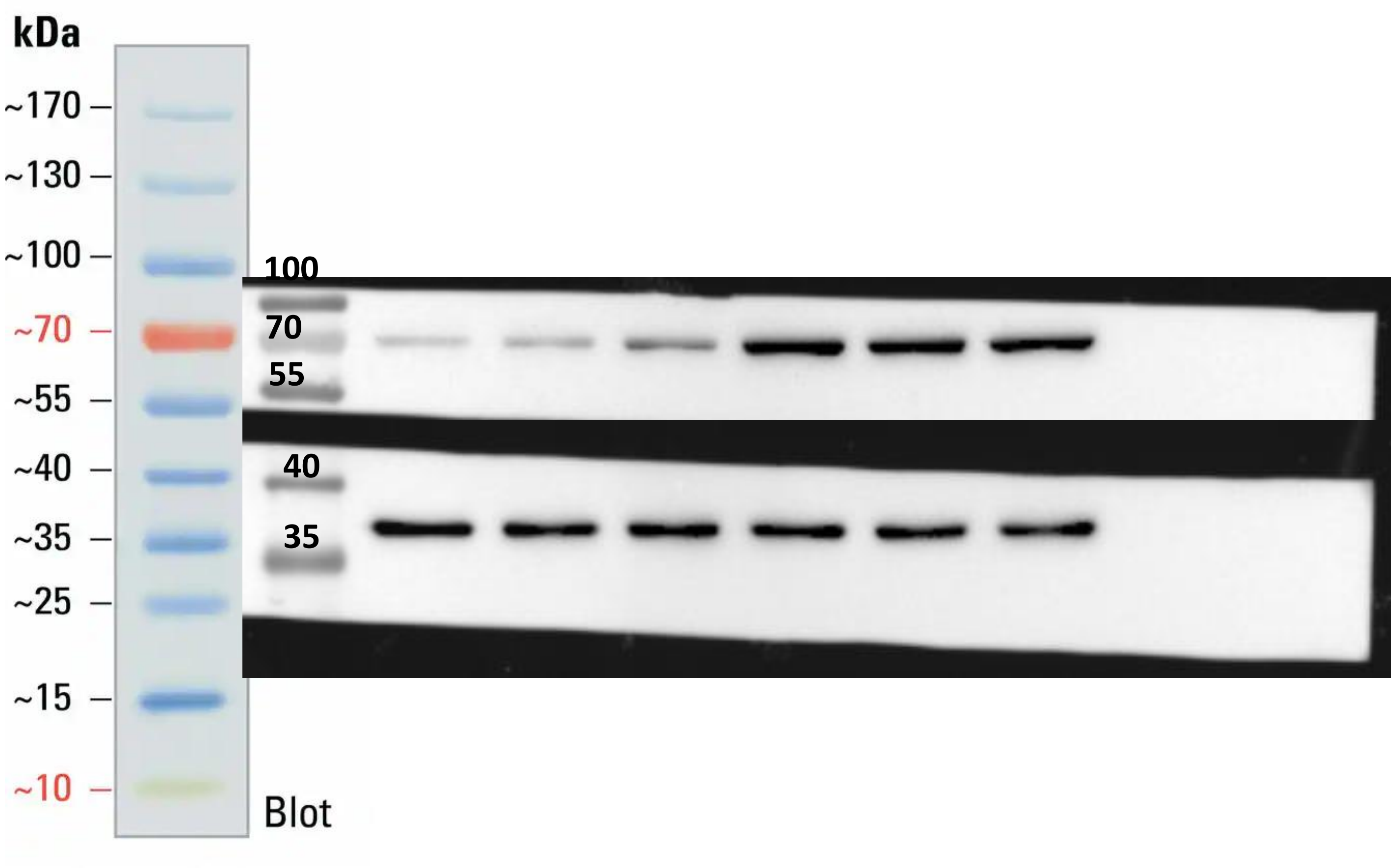

HSPA1A-12% gel

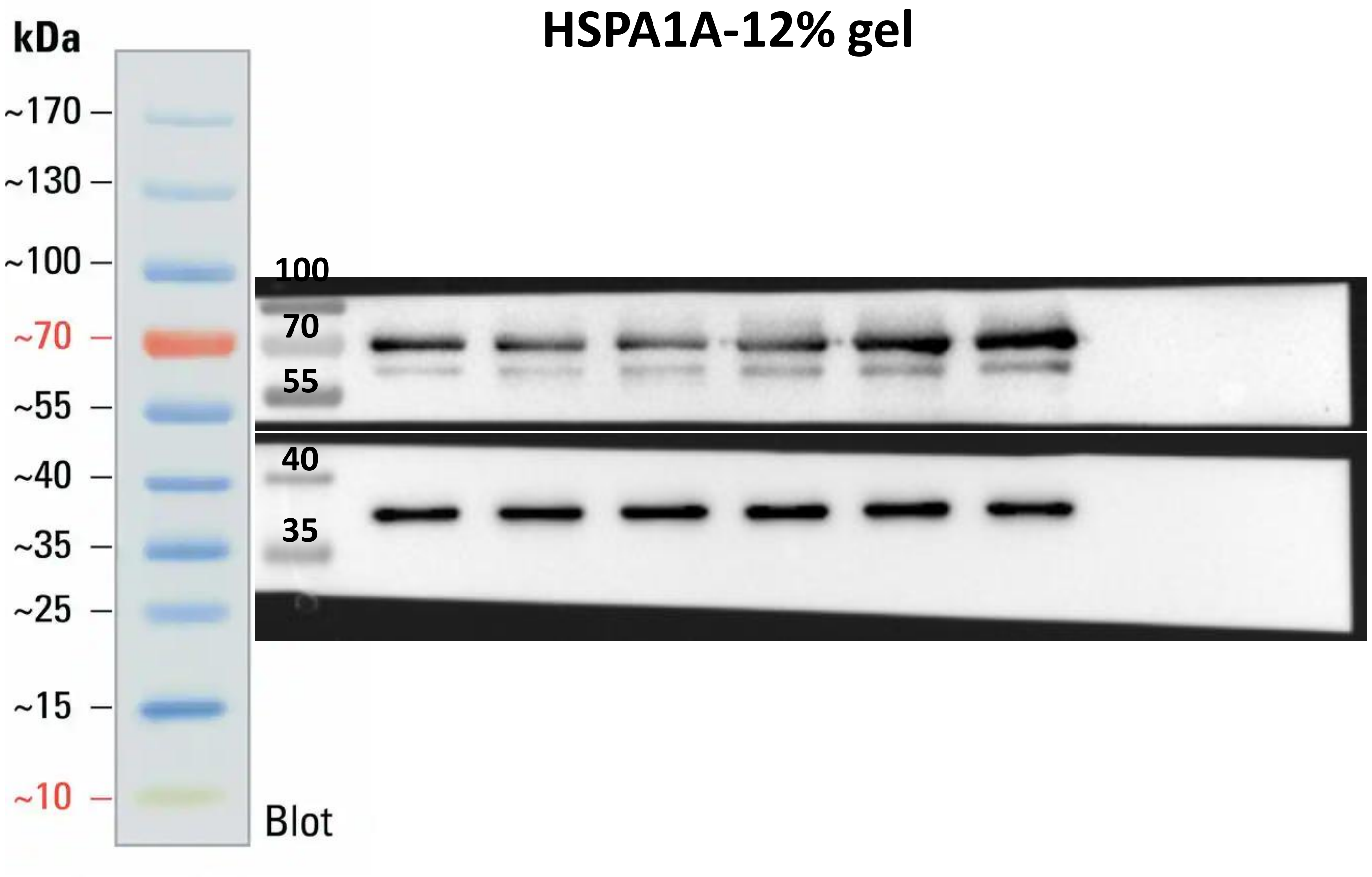

HSPA1L-15% gel

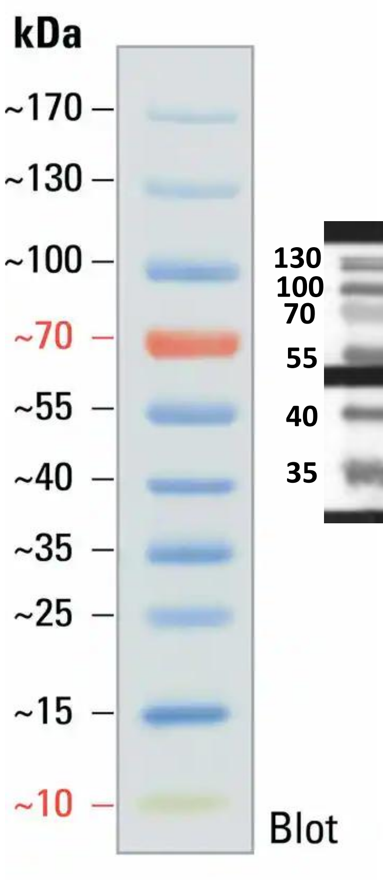

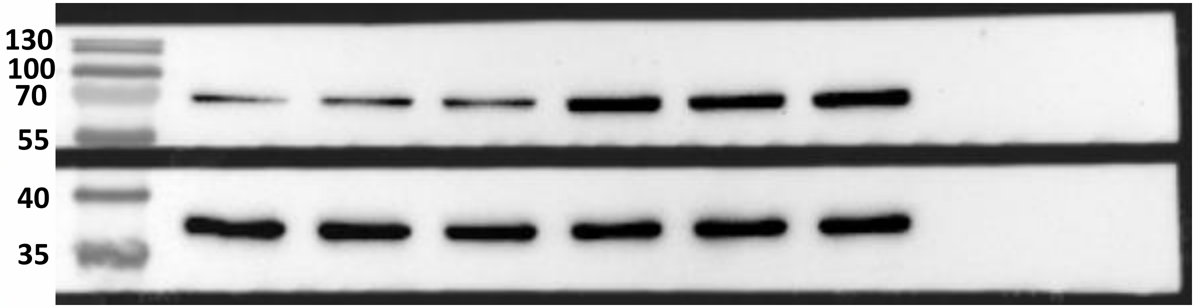

HSPA4L-10% gel

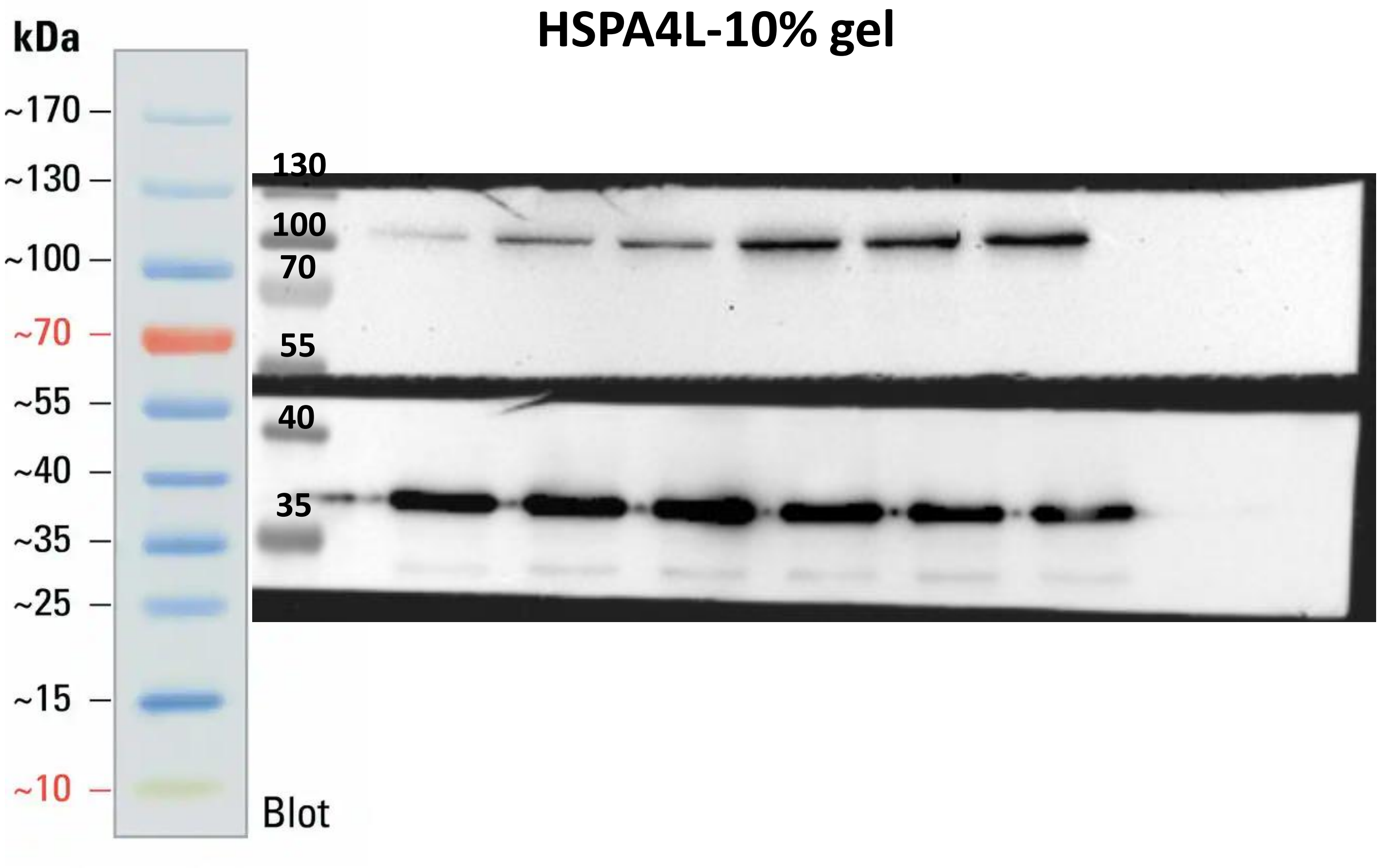

HSPA4L-12% gel

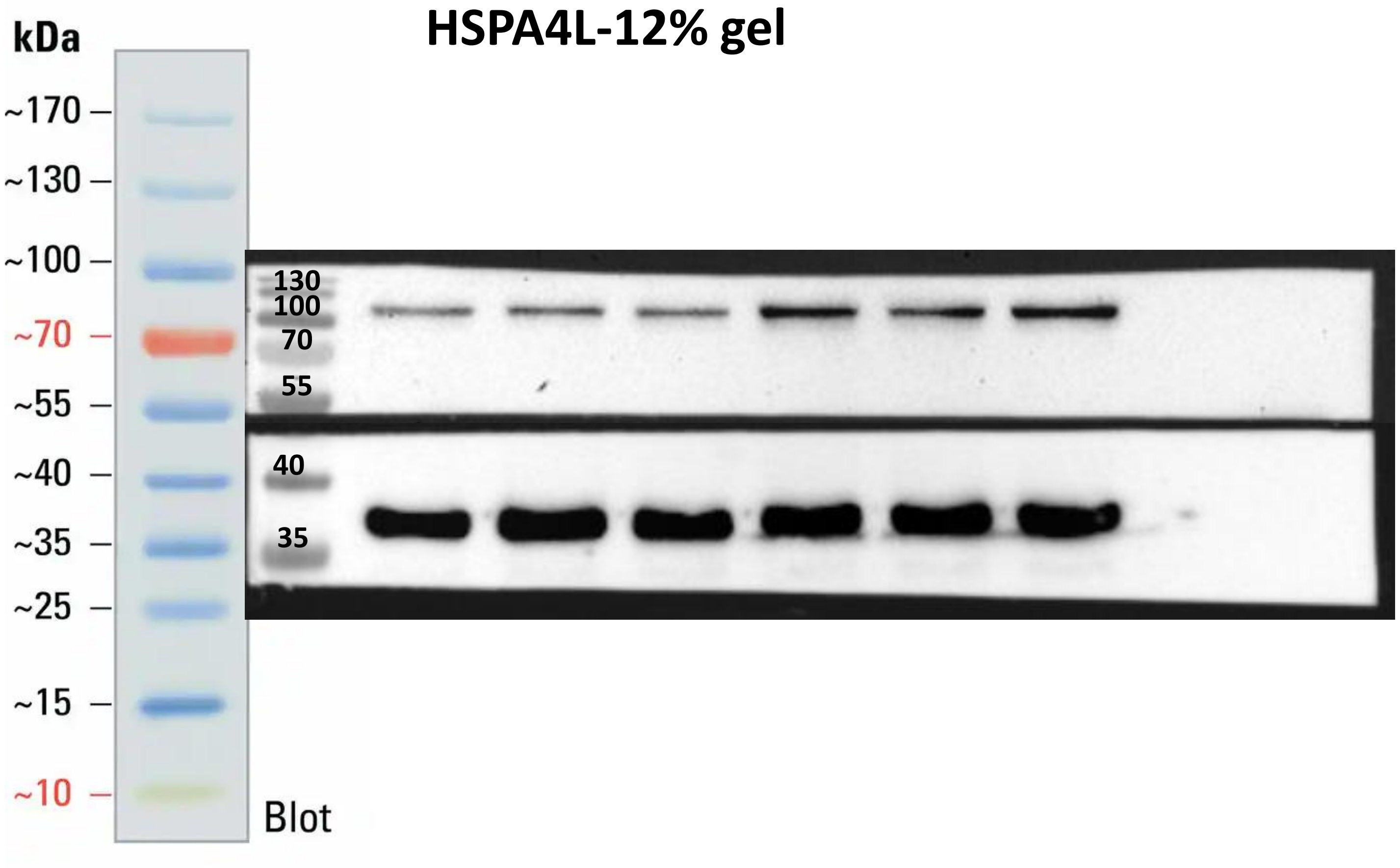

DANJB1-12% gel

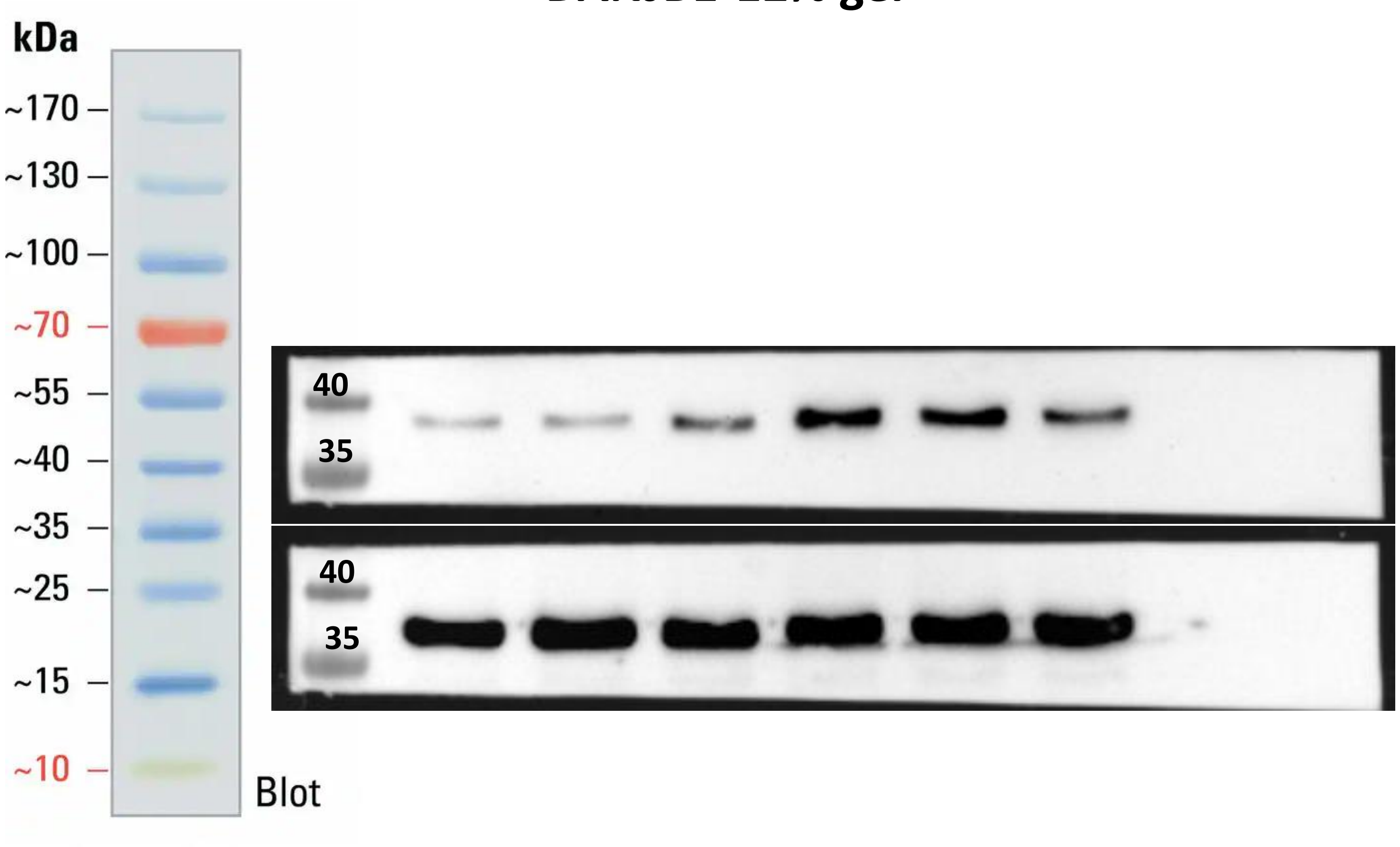

The blot was first probed by anti-DNAJB1 followed by anti-GAPDH

BAG3-10% gel

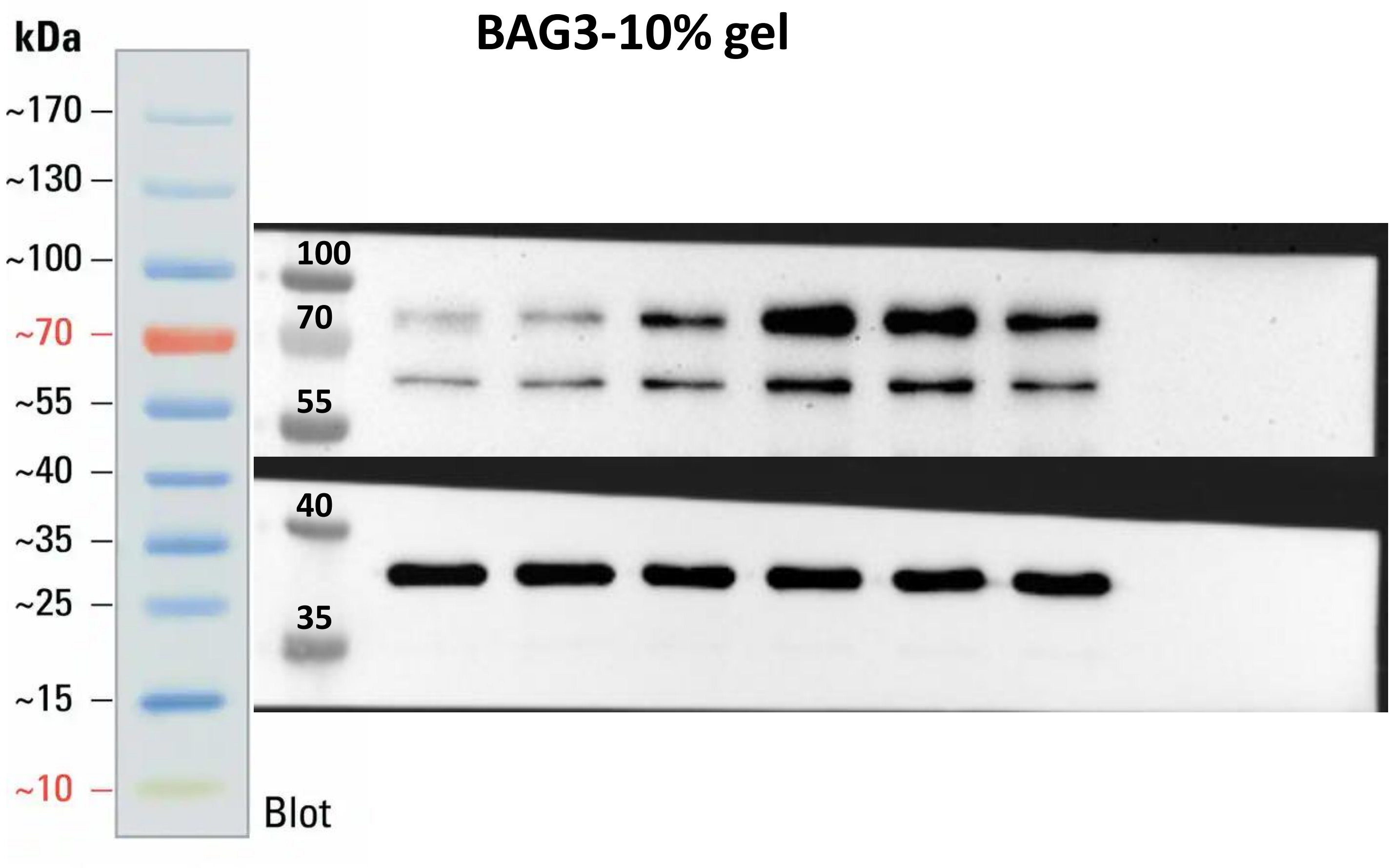

HSPH1-10% gel

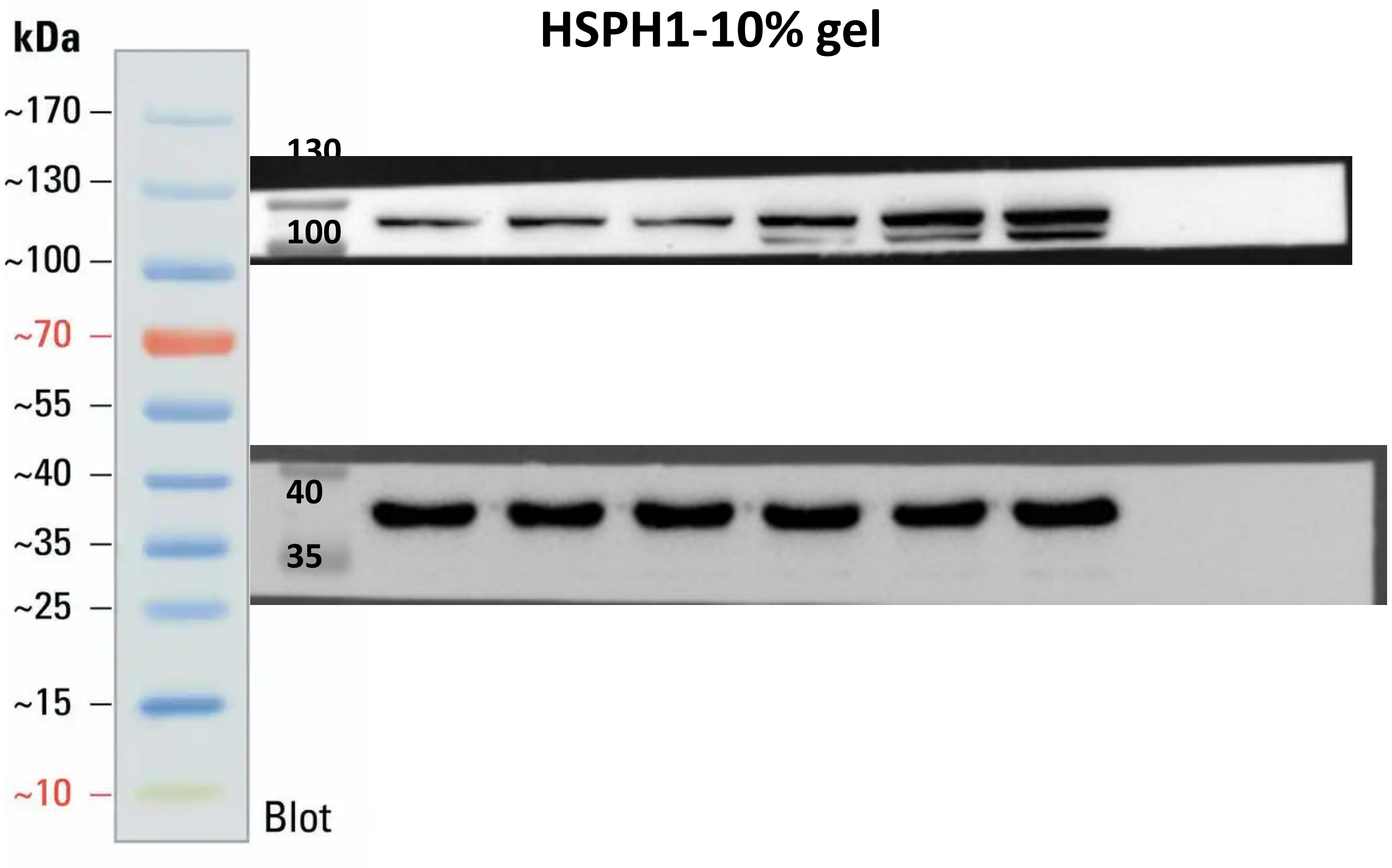

HSPH1-8% gel

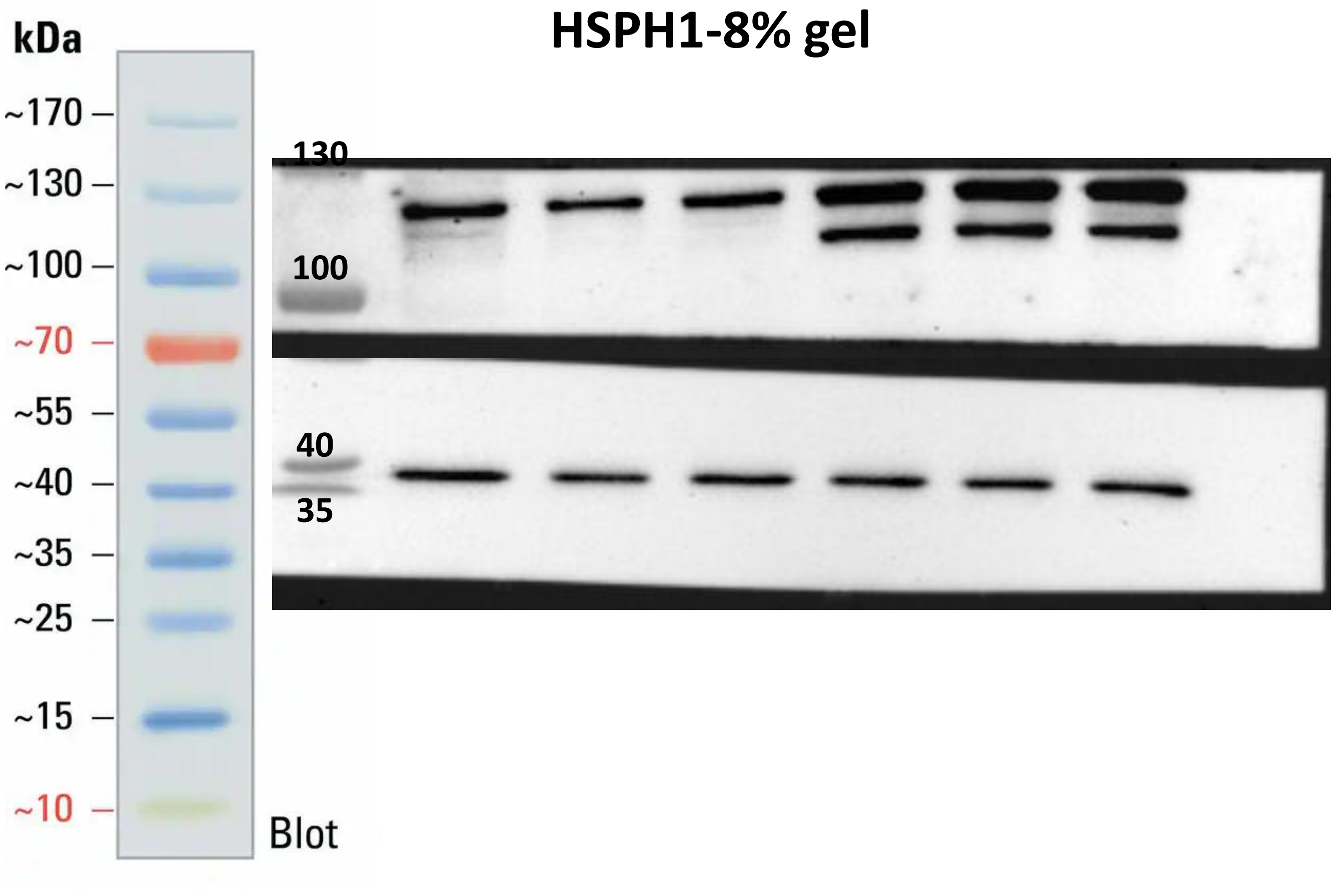

Supplement: Supplementary file 1 [file biology-12-00416-s001.zip › biology-2248156-supplementary-final/File S1-WB IMAGES-For Submission/2. MW Confirmation/MW detection.pdf]
