# Supplementary material for: Profiling the Hsp70 Chaperone Network in Heat-Induced Proteotoxic Stress Models of Human Neurons
Source: Biology (Basel). 2023 Mar 9;12(3):416. doi: 10.3390/biology12030416 (PMC10045125; doi:10.3390/biology12030416)
Supplement: Supplementary file 1 [file biology-12-00416-s001.zip › biology-2248156-supplementary-final/SUPPLEMENTARY/supplementary figures.pdf]

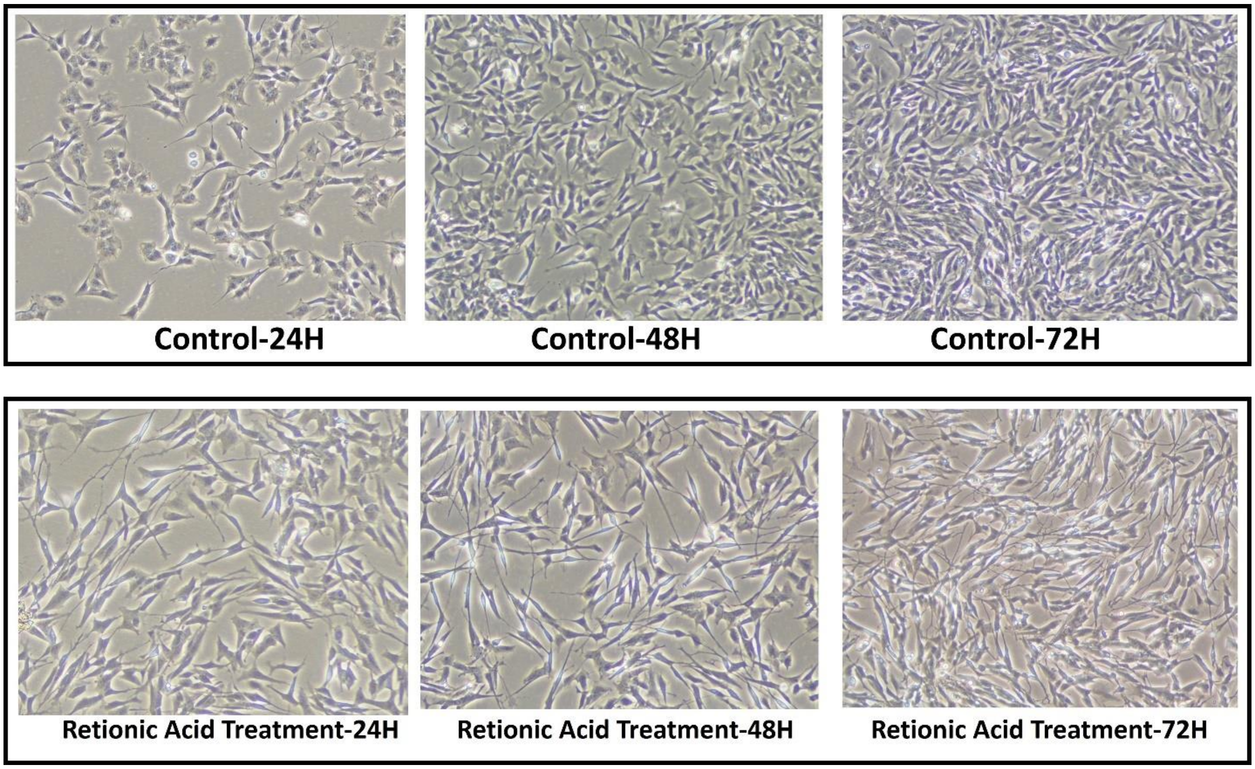

Figure S1. Retinoic acid differentiation of SH-SY5Y cells into terminally differentiated human neurons. *Top Panel*: Control cells i.e., untreated cells observed at 24-, 48-, and 72- hours. *Lower Panel*: Retinoic acid treated cells observed at 24-, 48-, and 72- hours. The differences are obvious between differentiated and undifferentiated cells like clustering of undifferentiated cells, and neurite extension in differentiated cells.

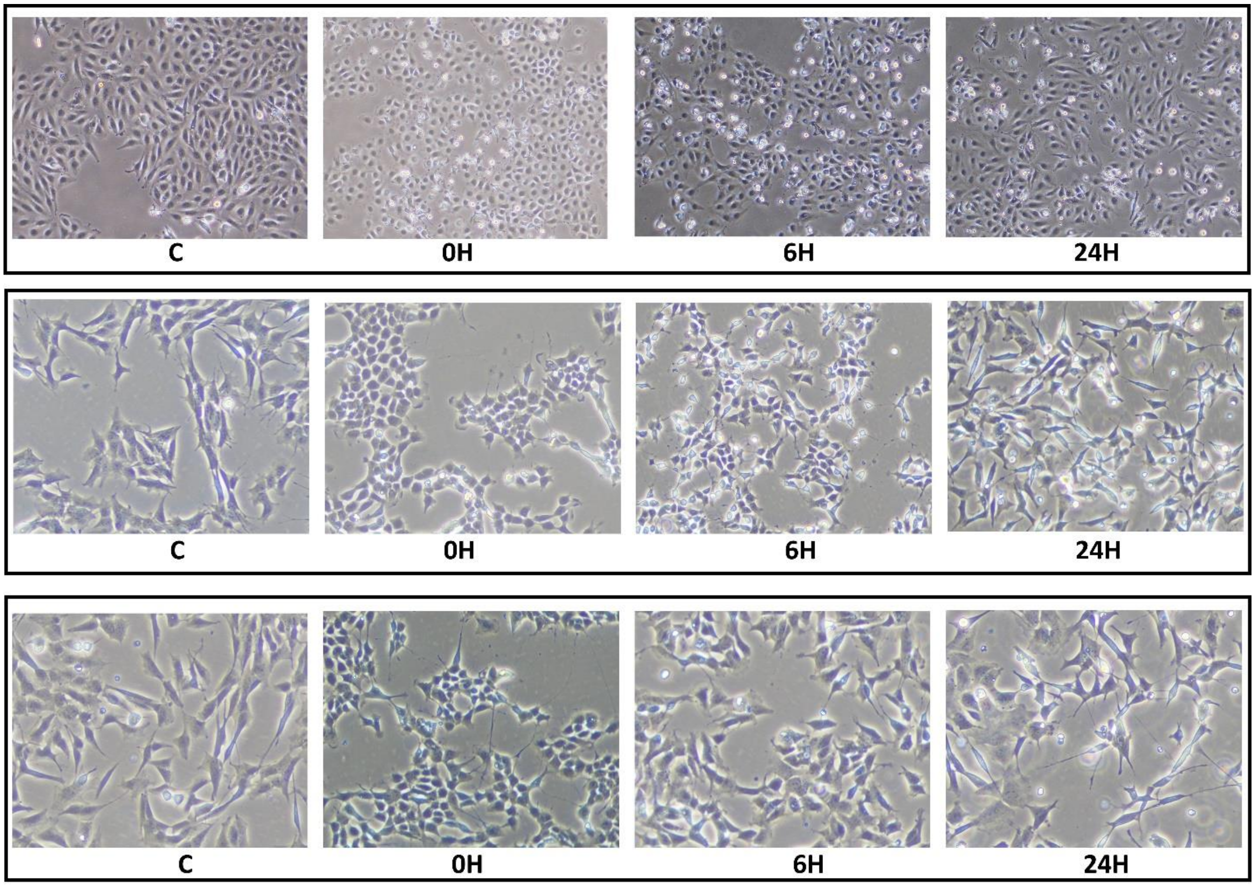

Figure S2. Cell culture and heat-stress experiments in *Top Panel*: Daoy Cells, *Middle Panel*: SH-SY5Y Cell, *Lower Panel*: Differentiated SH-SY5Y Cells. On the Left is Control cells, Middle is 0H i.e., immediately after heat-stress, and Extreme Right is cells recovered 24-hours post heat-stress exposure.

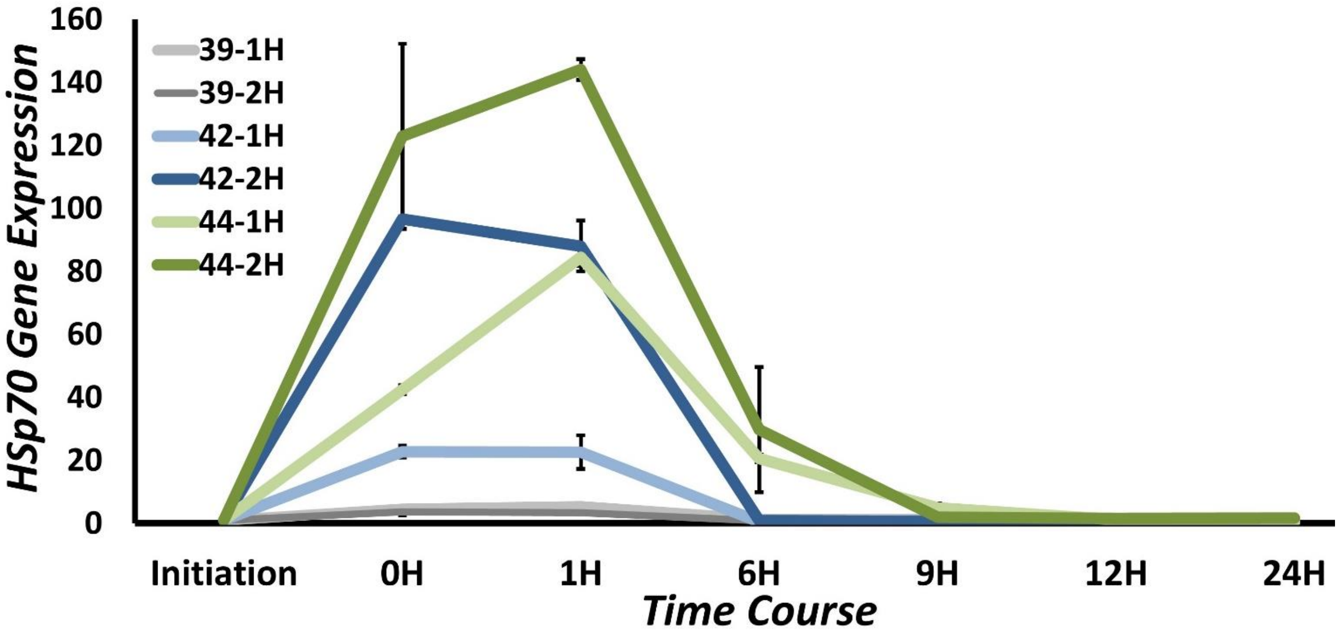

Figure S3. Dose-responsive nature of heat stress in SH-SY5Y cells. Time-course analysis of Hsp70 gene expression induction at 39 °C, 42 °C, and 44 °C for 1 and 2 hours. As the magnitude of heat stress increases, the heat shock response rises proportionately.

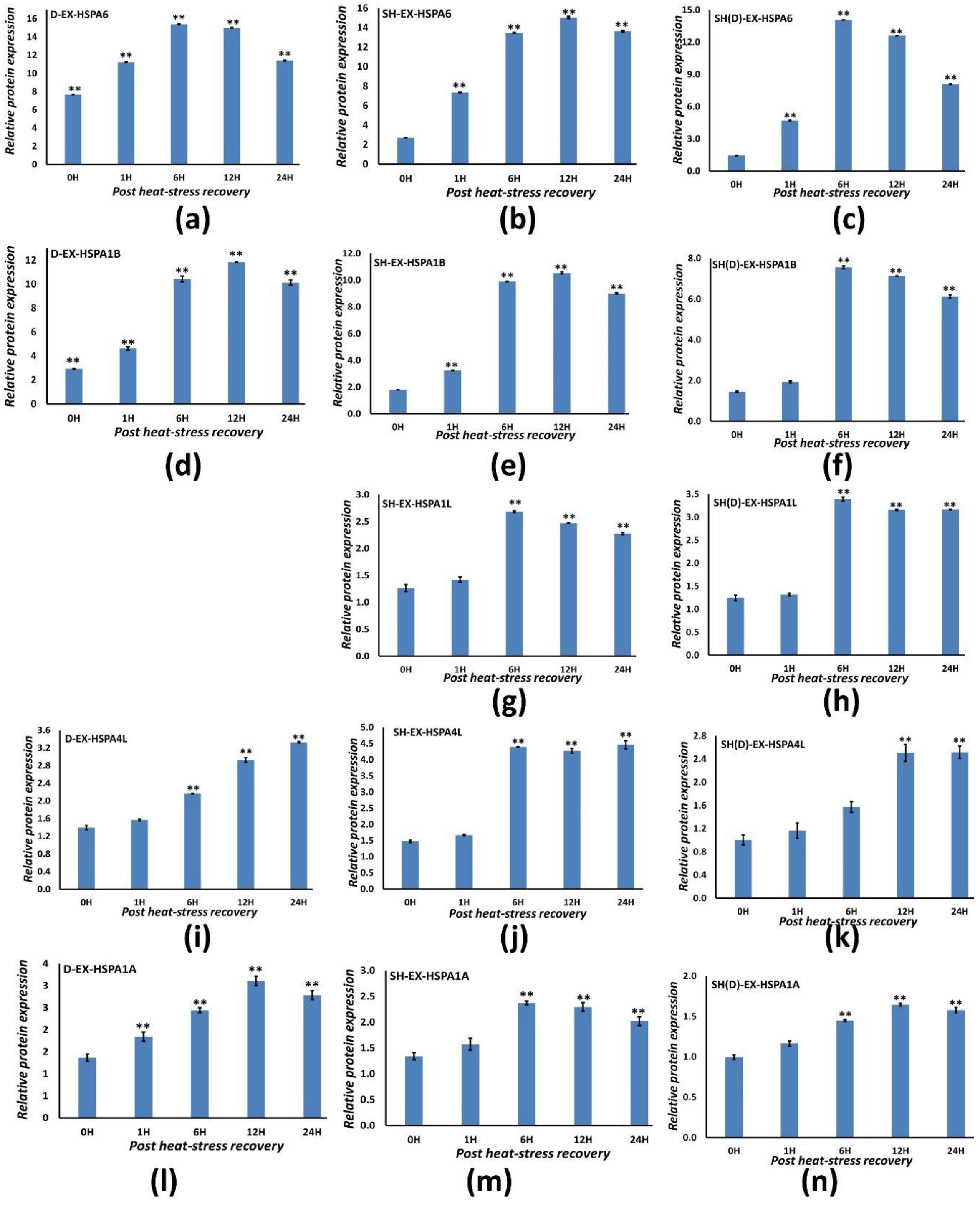

Figure S4. Western blot analysis of Hsp70 members. Quantifications (Western blot images in Figures 5 a-c) show the expression levels of HSPA6 (a-c), HSPA1B(d-f), HSPA1L(g,h), HSPA4L (i-k), and HSPA1A (l-n) proteins. Bar graphs were used for quantification of the expression levels as compared to GAPDH control. Unpaired t-test in was used to calculate p values, **$p < 0.05$. Fold change values corresponding to this data are plotted in heat-maps (Figures 5 d-f) and Smoothened scatterplots (Figures 5 g-i).

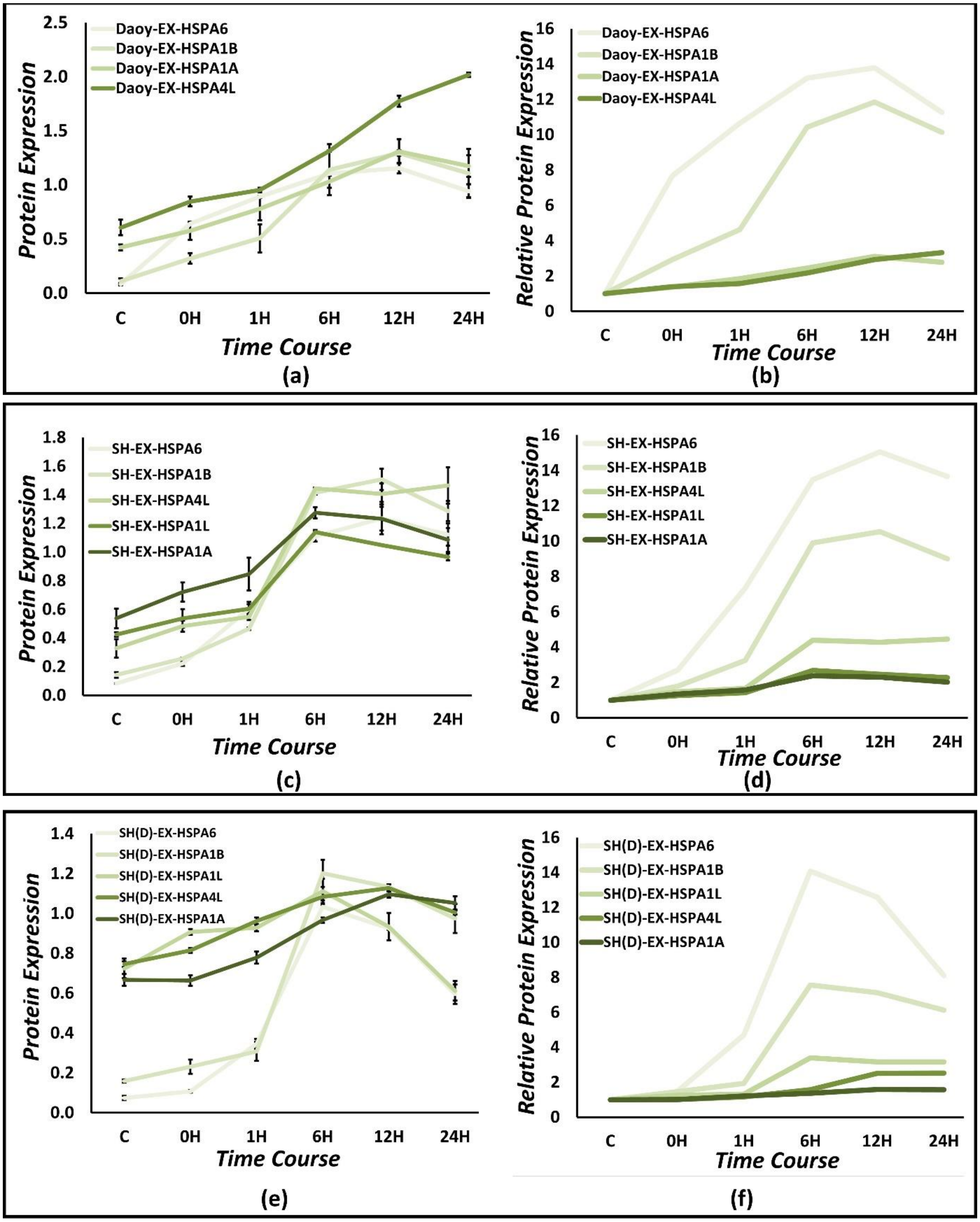

Figure S5. Western blot quantification time plots of Hsp70. a, c, and e are plot for protein fold-expression change as compared to GAPDH control. b,d, and e are plot for protein fold-expression change as compared to protein expression in control samples i.e., cells growing at 37°C.

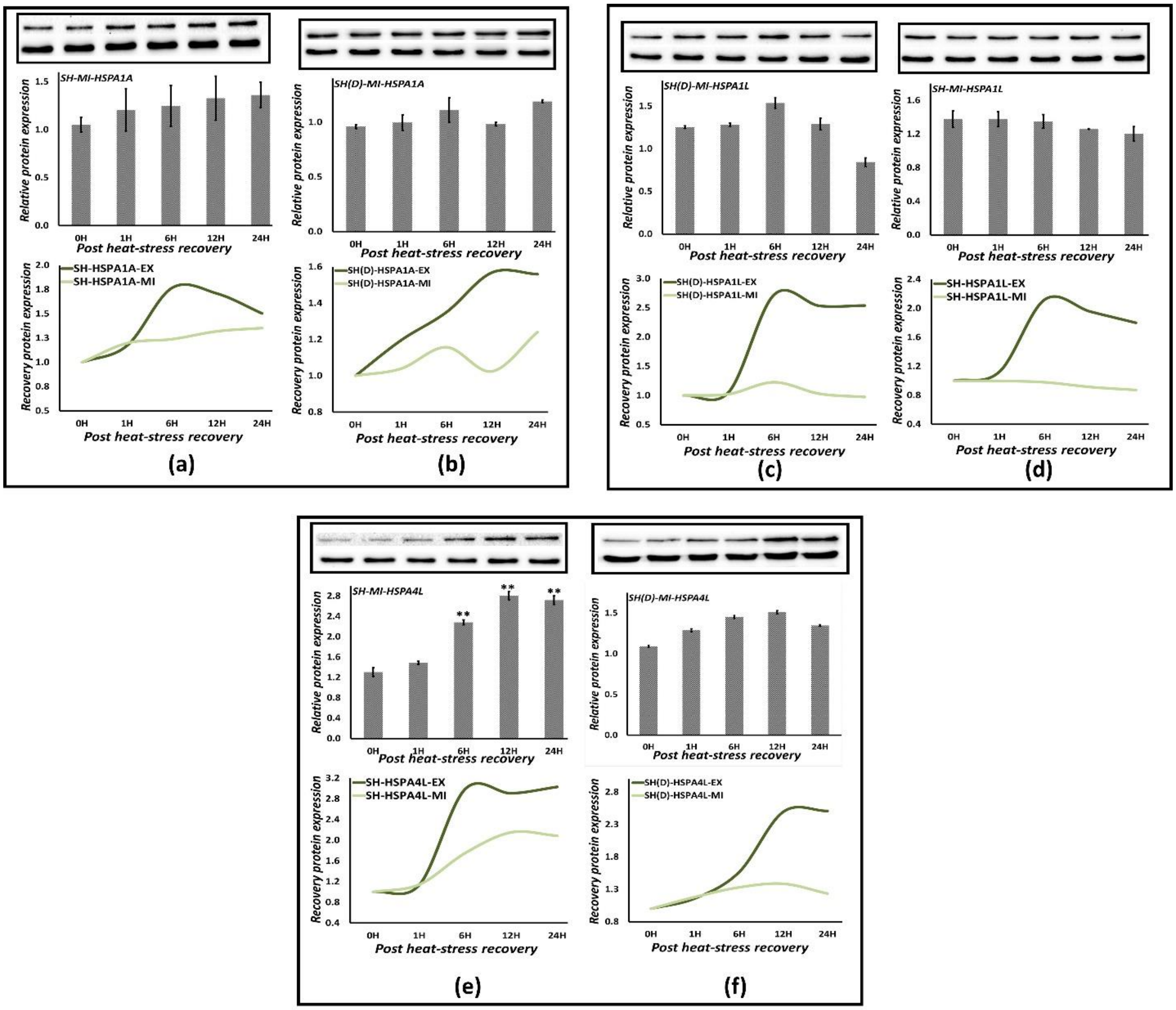

Figure S6. Western blot images and quantifications of Hsp70 members in mild heat-stress conditions in SH-SY5Y and differentiated SH-SY5Y cells. HSPA1A: a, b, HSPA1L: c, d, and HSPA4L: e, f. Bar graphs were used for quantification of the expression levels as compared to GAPDH control. Smoothened scatterplots represent the comparison with protein-expression in extreme conditions. HSPA6 and HSPA1B are plotted in Figure 7.

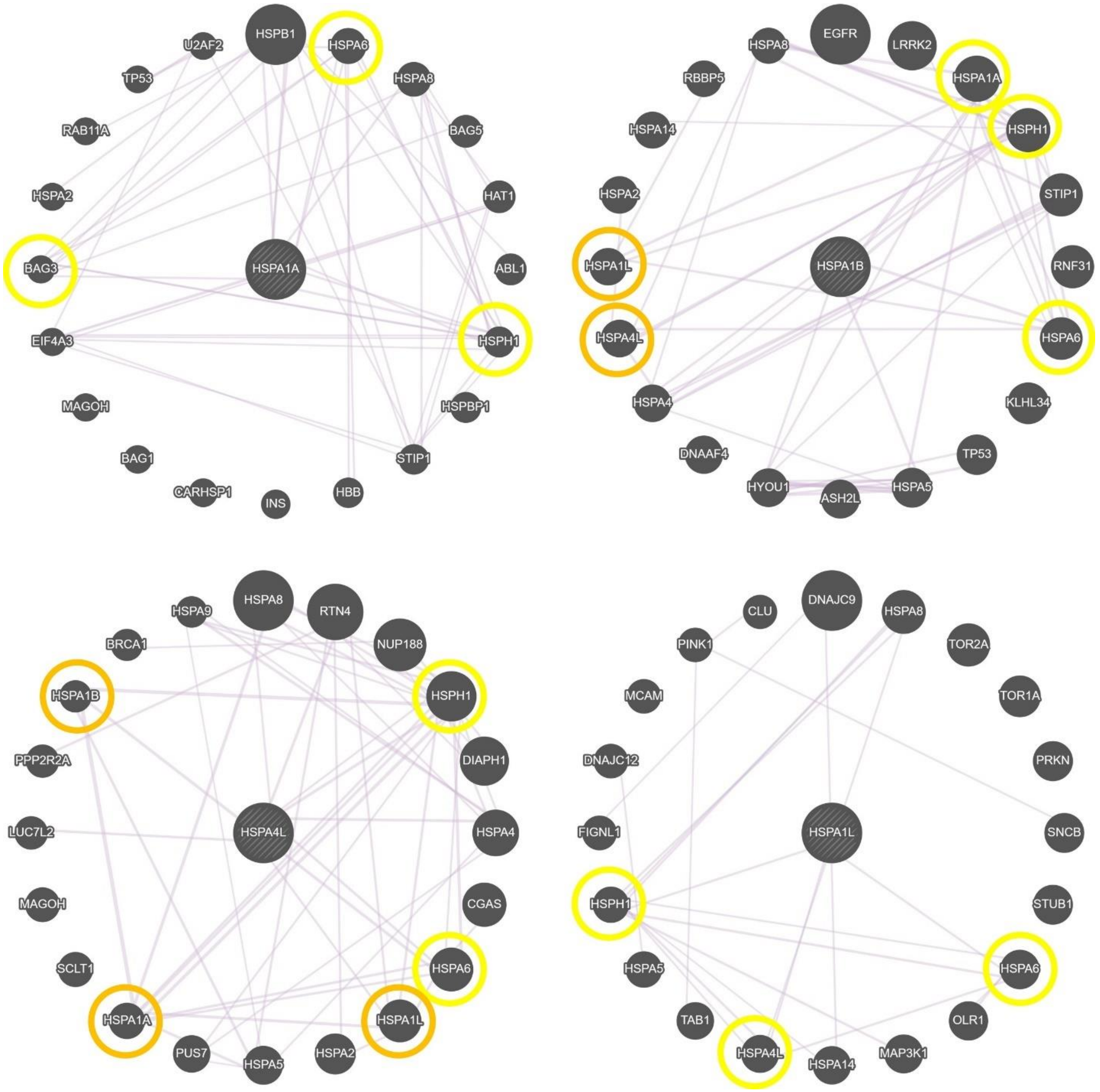

Figure S7. Network Analysis. GeneMania diagram showing co-expressed interactions of Hsp70 members HSPA1A, HSPA1B, HSPA4L, and HSPA1L The circled genes exist in the analysis from Heat Shock Proteins & Chaperones PCR Array.

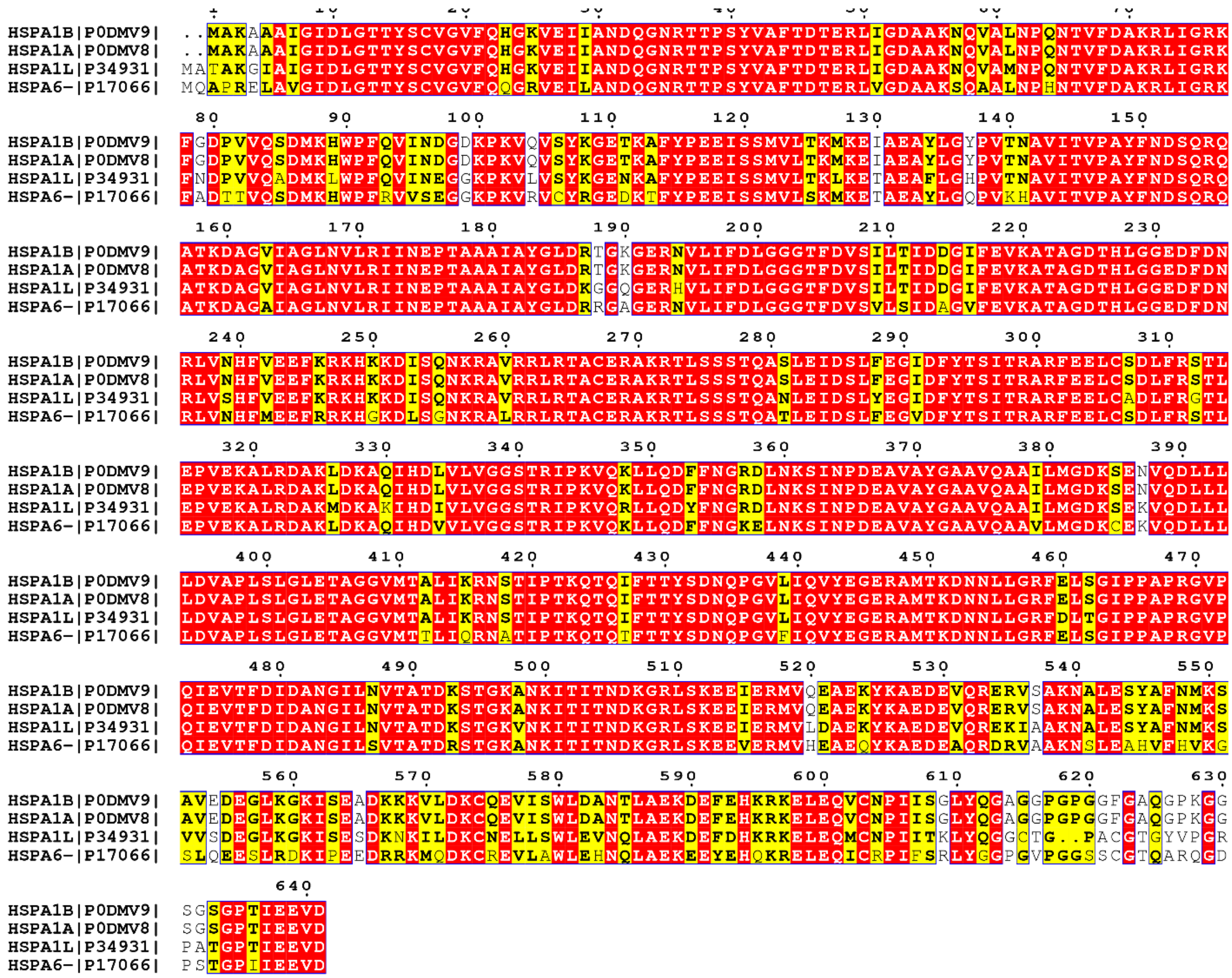

Figure S8. Multiple Sequence Alignment among Hsp70 members HSPA1A, HSPA1B, HSPA4L, and HSPA1L. The red regions highlight the identical regions among these proteins.
