# Supplementary material for: Profiling the Hsp70 Chaperone Network in Heat-Induced Proteotoxic Stress Models of Human Neurons
Source: Biology (Basel). 2023 Mar 9;12(3):416. doi: 10.3390/biology12030416 (PMC10045125; doi:10.3390/biology12030416)
Supplement: Supplementary file 1 [file biology-12-00416-s001.zip › biology-2248156-supplementary-final/SUPPLEMENTARY/supplementary tables.pdf]

Table S1: Gene List for Human Heat Shock Proteins & Chaperones RT² Profiler PCR Array (Qiagen) allows for simultaneous expression analysis of 84 genes from the HSP90 Family (81 to 99 kDa), HSP70 Family (65 to 80 kDa), HSP60 Family (55 to 64 kDa), HSP40 Family (35 to 54 kDa), small HSPs (<34 kDa), and Chaperone cofactors.

Position	UniGene	GenBank	Symbol	Description
A01	Hs.118241	NM_020247	ADCK3	AarF domain containing kinase 3
A02	Hs.492740	NM_007348	ATF6	Activating transcription factor 6
A03	Hs.377484	NM_004323	BAG1	BCL2-associated athanogene
A04	Hs.729098	NM_004282	BAG2	BCL2-associated athanogene 2
A05	Hs.523309	NM_004281	BAG3	BCL2-associated athanogene 3
A06	Hs.194726	NM_004874	BAG4	BCL2-associated athanogene 4
A07	Hs.5443	NM_004873	BAG5	BCL2-associated athanogene 5
A08	Hs.502917	NM_005125	CCS	Copper chaperone for superoxide dismutase
A09	Hs.189772	NM_006431	CCT2	Chaperonin containing TCP1, subunit 2 (beta)
A10	Hs.491494	NM_005998	CCT3	Chaperonin containing TCP1, subunit 3 (gamma)
A11	Hs.421509	NM_006430	CCT4	Chaperonin containing TCP1, subunit 4 (delta)
A12	Hs.1600	NM_012073	CCT5	Chaperonin containing TCP1, subunit 5 (epsilon)
B01	Hs.82916	NM_001762	CCT6A	Chaperonin containing TCP1, subunit 6A (zeta 1)
B02	Hs.73072	NM_006584	CCT6B	Chaperonin containing TCP1, subunit 6B (zeta 2)
B03	Hs.368149	NM_006429	CCT7	Chaperonin containing TCP1, subunit 7 (eta)
B04	Hs.184085	NM_000394	CRYAA	Crystallin, alpha A
B05	Hs.408767	NM_001885	CRYAB	Crystallin, alpha B
B06	Hs.445203	NM_001539	DNAJA1	DnaJ (Hsp40) homolog, subfamily A, member 1
B07	Hs.368078	NM_005880	DNAJA2	DnaJ (Hsp40) homolog, subfamily A, member 2
B08	Hs.459779	NM_005147	DNAJA3	DnaJ (Hsp40) homolog, subfamily A, member 3
B09	Hs.513053	NM_018602	DNAJA4	DnaJ (Hsp40) homolog, subfamily A, member 4
B10	Hs.515210	NM_006145	DNAJB1	DnaJ (Hsp40) homolog, subfamily B, member 1
B11	Hs.317192	NM_016306	DNAJB11	DnaJ (Hsp40) homolog, subfamily B, member 11
B12	Hs.696014	NM_017626	DNAJB12	DnaJ (Hsp40) homolog, subfamily B, member 12
C01	Hs.567888	NM_153614	DNAJB13	DnaJ (Hsp40) homolog, subfamily B, member 13
C02	Hs.577426	NM_001031723	DNAJB14	DnaJ (Hsp40) homolog, subfamily B, member 14
C03	Hs.77768	NM_006736	DNAJB2	DnaJ (Hsp40) homolog, subfamily B, member 2
C04	Hs.237506	NM_012266	DNAJB5	DnaJ (Hsp40) homolog, subfamily B, member 5
C05	Hs.490745	NM_005494	DNAJB6	DnaJ (Hsp40) homolog, subfamily B, member 6
C06	Hs.585042	NM_145174	DNAJB7	DnaJ (Hsp40) homolog, subfamily B, member 7
C07	Hs.518241	NM_153330	DNAJB8	DnaJ (Hsp40) homolog, subfamily B, member 8
C08	Hs.6790	NM_012328	DNAJB9	DnaJ (Hsp40) homolog, subfamily B, member 9
C09	Hs.499000	NM_022365	DNAJC1	DnaJ (Hsp40) homolog, subfamily C, member 1
C10	Hs.516632	NM_018981	DNAJC10	DnaJ (Hsp40) homolog, subfamily C, member 10
C11	Hs.462640	NM_018198	DNAJC11	DnaJ (Hsp40) homolog, subfamily C, member 11
C12	Hs.260720	NM_201262	DNAJC12	DnaJ (Hsp40) homolog, subfamily C, member 12
D01	Hs.12707	NM_015268	DNAJC13	DnaJ (Hsp40) homolog, subfamily C, member 13
D02	Hs.709320	NM_032364	DNAJC14	DnaJ (Hsp40) homolog, subfamily C, member 14
D03	Hs.438830	NM_013238	DNAJC15	DnaJ (Hsp40) homolog, subfamily C, member 15
D04	Hs.655410	NM_015291	DNAJC16	DnaJ (Hsp40) homolog, subfamily C, member 16
D05	Hs.511069	NM_018163	DNAJC17	DnaJ (Hsp40) homolog, subfamily C, member 17
D06	Hs.483537	NM_152686	DNAJC18	DnaJ (Hsp40) homolog, subfamily C, member 18
D07	Hs.131887	NM_194283	DNAJC21	DnaJ (Hsp40) homolog, subfamily C, member 21
D08	Hs.59214	NM_006260	DNAJC3	DnaJ (Hsp40) homolog, subfamily C, member 3
D09	Hs.172847	NM_005528	DNAJC4	DnaJ (Hsp40) homolog, subfamily C, member 4

Position	UniGene	GenBank	Symbol	Description
D10	Hs.164419	NM_025219	DNAJC5	DnaJ (Hsp40) homolog, subfamily C, member 5
D11	Hs.491885	NM_033105	DNAJC5B	DnaJ (Hsp40) homolog, subfamily C, member 5 beta
D12	Hs.116303	NM_173650	DNAJC5G	DnaJ (Hsp40) homolog, subfamily C, member 5 gamma
E01	Hs.647643	NM_014787	DNAJC6	DnaJ (Hsp40) homolog, subfamily C, member 6
E02	Hs.500156	NM_003315	DNAJC7	DnaJ (Hsp40) homolog, subfamily C, member 7
E03	Hs.433540	NM_014280	DNAJC8	DnaJ (Hsp40) homolog, subfamily C, member 8
E04	Hs.654694	NM_015190	DNAJC9	DnaJ (Hsp40) homolog, subfamily C, member 9
E05	Hs.530227	NM_005526	HSF1	Heat shock transcription factor 1
E06	Hs.158195	NM_004506	HSF2	Heat shock transcription factor 2
E07	Hs.512156	NM_001538	HSF4	Heat shock transcription factor 4
E08	Hs.525600	NM_001017963	HSP90AA1	Heat shock protein 90kDa alpha (cytosolic), class A member 1
E09	Hs.509736	NM_007355	HSP90AB1	Heat shock protein 90kDa alpha (cytosolic), class B member 1
E10	Hs.192374	NM_003299	HSP90B1	Heat shock protein 90kDa beta (Grp94), member 1
E11	Hs.534169	NM_016299	HSPA14	Heat shock 70kDa protein 14
E12	Hs.728810	NM_005345	HSPA1A	Heat shock 70kDa protein 1A
F01	Hs.274402	NM_005346	HSPA1B	Heat shock 70kDa protein 1B
F02	Hs.690634	NM_005527	HSPA1L	Heat shock 70kDa protein 1-like
F03	Hs.728938	NM_021979	HSPA2	Heat shock 70kDa protein 2
F04	Hs.90093	NM_002154	HSPA4	Heat shock 70kDa protein 4
F05	Hs.135554	NM_014278	HSPA4L	Heat shock 70kDa protein 4-like
F06	Hs.716396	NM_005347	HSPA5	Heat shock 70kDa protein 5 (glucose-regulated protein, 78kDa)
F07	Hs.654614	NM_002155	HSPA6	Heat shock 70kDa protein 6 (HSP70B')
F08	Hs.702021	NM_006597	HSPA8	Heat shock 70kDa protein 8
F09	Hs.184233	NM_004134	HSPA9	Heat shock 70kDa protein 9 (mortalin)
F10	Hs.520973	NM_001540	HSPB1	Heat shock 27kDa protein 1
F11	Hs.709660	NM_001541	HSPB2	Heat shock 27kDa protein 2
F12	Hs.41707	NM_006308	HSPB3	Heat shock 27kDa protein 3
G01	Hs.534538	NM_144617	HSPB6	Heat shock protein, alpha-crystallin-related, B6
G02	Hs.502612	NM_014424	HSPB7	Heat shock 27kDa protein family, member 7 (cardiovascular)
G03	Hs.400095	NM_014365	HSPB8	Heat shock 22kDa protein 8
G04	Hs.595053	NM_002156	HSPD1	Heat shock 60kDa protein 1 (chaperonin)
G05	Hs.1197	NM_002157	HSPE1	Heat shock 10kDa protein 1 (chaperonin 10)
G06	Hs.36927	NM_006644	HSPH1	Heat shock 105kDa/110kDa protein 1
G07	Hs.483564	NM_002622	PFDN1	Prefoldin subunit 1
G08	Hs.492516	NM_012394	PFDN2	Prefoldin subunit 2
G09	Hs.596449	NM_001235	SERPINH1	Serpin peptidase inhibitor, clade H (heat shock protein 47), member 1, (collagen binding protein 1)
G10	Hs.483521	NM_022464	SIL1	SIL1 homolog, endoplasmic reticulum chaperone (S. cerevisiae)
G11	Hs.363137	NM_030752	TCP1	T-complex 1
G12	Hs.534312	NM_000113	TOR1A	Torsin family 1, member A (torsin A)
H01	Hs.520640	NM_001101	ACTB	Actin, beta
H02	Hs.534255	NM_004048	B2M	Beta-2-microglobulin
H03	Hs.592355	NM_002046	GAPDH	Glyceraldehyde-3-phosphate dehydrogenase
H04	Hs.412707	NM_000194	HPRT1	Hypoxanthine phosphoribosyltransferase 1
H05	Hs.546285	NM_001002	RPLP0	Ribosomal protein, large, P0
H06	N/A	SA_00105	HGDC	Human Genomic DNA Contamination
H07	N/A	SA_00104	RTC	Reverse Transcription Control
H08	N/A	SA_00104	RTC	Reverse Transcription Control
H09	N/A	SA_00104	RTC	Reverse Transcription Control
H10	N/A	SA_00103	PPC	Positive PCR Control
H11	N/A	SA_00103	PPC	Positive PCR Control
H12	N/A	SA_00103	PPC	Positive PCR Control

Table S2: Correlation coefficients (r) and p-values for the co-expression analysis of HSPA6 and HSPH1 in SH-SY5Y cells and differentiated SH-SY5Y cells. The analysis was performed using the protein expression fold change data.

**SH-SY5Y**						
**HSPA6**	**r**	**p**		**HSPH1**	**r**	**p-value**
HSPA1A	0.95	0.012		HSPA6	0.95	0.01
HSPA1B	0.98	0.0016		HSPA1B	1.00	0.0001
HSPA4L	0.95	0.044		HSPA1A	0.98	0.004
HSPA1L	0.95	0.018		HSPA4L	0.98	0.003
HSPH1	0.95	0.01		HSPA1L	0.98	0.002
DNAJB1	0.92	0.025		DNAJB1	0.93	0.021
BAG3	0.86	0.064				
**SH-SY5Y(D)**						
**HSPA6**	**r**	**p**		**HSPH1**	**r**	**p-value**
HSPA1A	0.84	0.07		HSPA6	0.87	0.057
HSPA1B	0.95	0.012		HSPA1B	0.96	0.008
HSPA4L	0.89	0.044		HSPA1A	0.98	0.004
HSPA1L	0.91	0.034		HSPA4L	0.91	0.035
HSPH1	0.87	0.057		HSPA1L	0.97	0.007
DNAJB1	0.95	0.013		DNAJB1	0.97	0.006
BAG3	0.97	0.006				
