# Supplementary figures and images for: Profiling the Hsp70 Chaperone Network in Heat-Induced Proteotoxic Stress Models of Human Neurons
Source: Biology (Basel). 2023 Mar 9;12(3):416. doi: 10.3390/biology12030416 (PMC10045125; doi:10.3390/biology12030416)

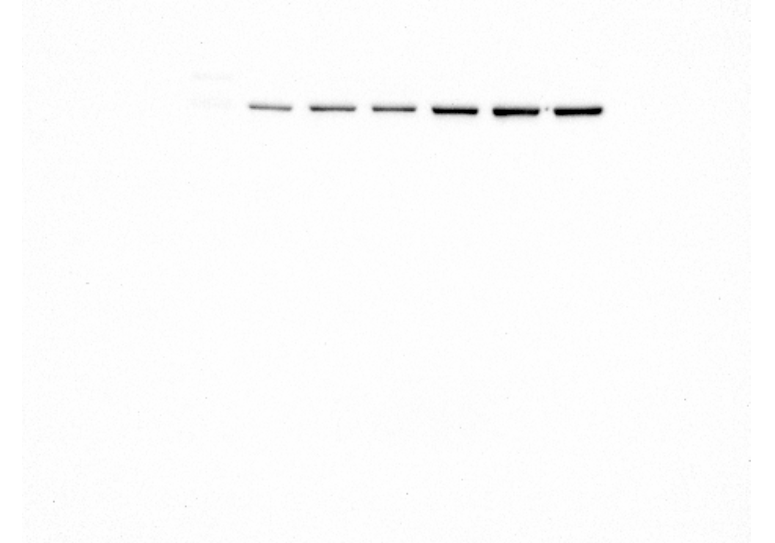

Supplement: Supplementary file 1 [file biology-12-00416-s001.zip › biology-2248156-supplementary-final/File S1-WB IMAGES-For Submission/1. MAIN MANUSCRIPT IMAGES/Daoy-EX/1.D-EX-HSPA1A.tif]

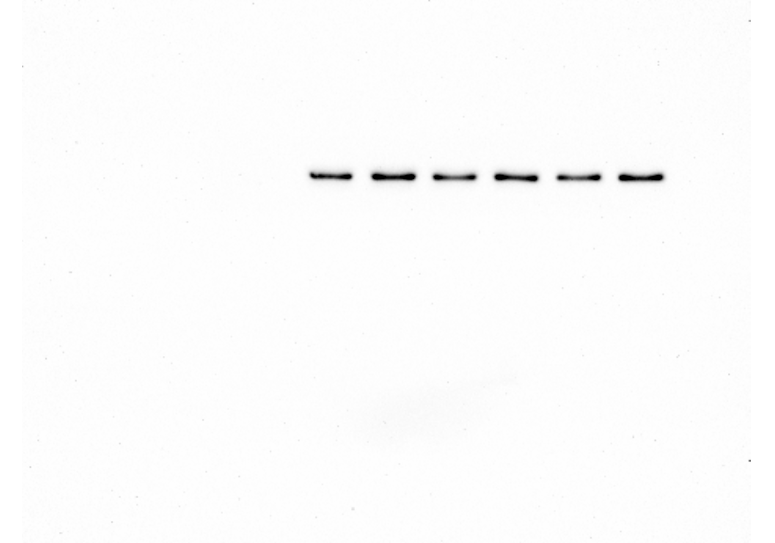

Supplement: Supplementary file 1 [file biology-12-00416-s001.zip › biology-2248156-supplementary-final/File S1-WB IMAGES-For Submission/1. MAIN MANUSCRIPT IMAGES/Daoy-EX/1C.D-EX-HSPA1A-G.tif]

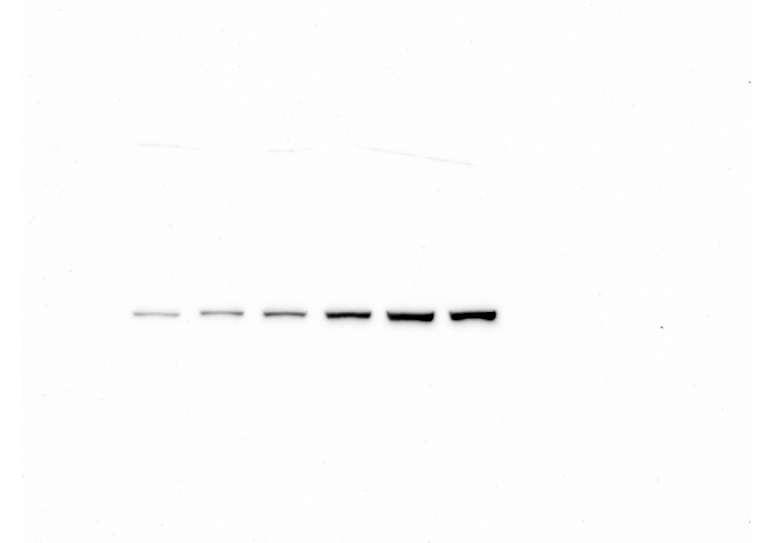

Supplement: Supplementary file 1 [file biology-12-00416-s001.zip › biology-2248156-supplementary-final/File S1-WB IMAGES-For Submission/1. MAIN MANUSCRIPT IMAGES/Daoy-EX/2.D-EX-HSPA1B.tif]

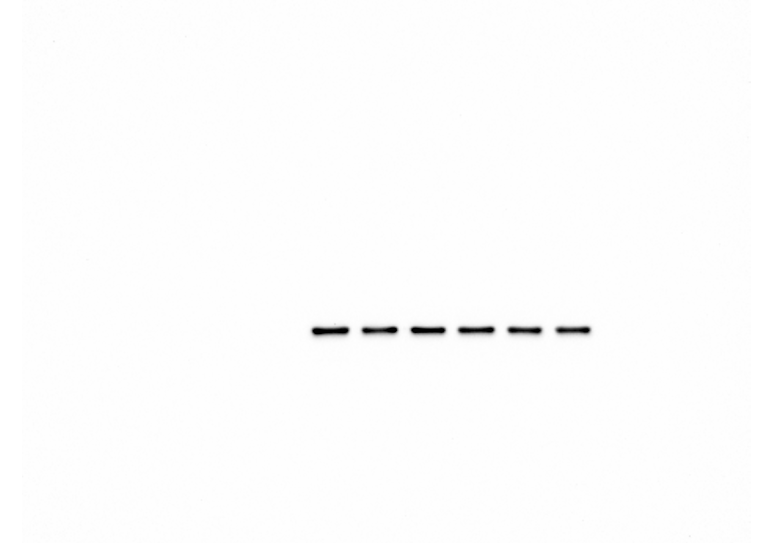

Supplement: Supplementary file 1 [file biology-12-00416-s001.zip › biology-2248156-supplementary-final/File S1-WB IMAGES-For Submission/1. MAIN MANUSCRIPT IMAGES/Daoy-EX/2C.D-EX-HSPA1B-G.tif]

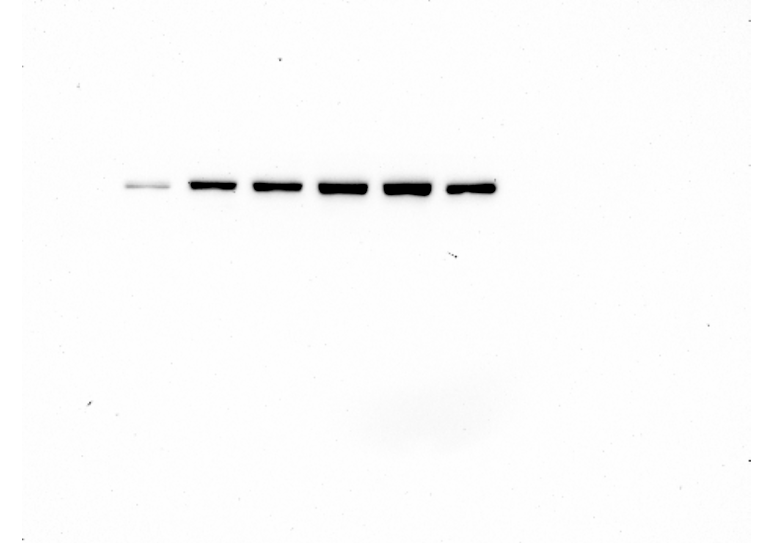

Supplement: Supplementary file 1 [file biology-12-00416-s001.zip › biology-2248156-supplementary-final/File S1-WB IMAGES-For Submission/1. MAIN MANUSCRIPT IMAGES/Daoy-EX/3.D-EX-HSPA6.tif]

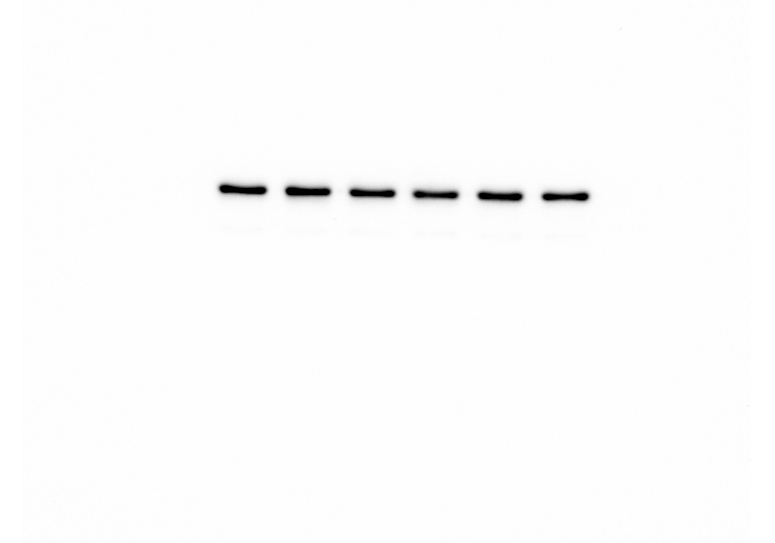

Supplement: Supplementary file 1 [file biology-12-00416-s001.zip › biology-2248156-supplementary-final/File S1-WB IMAGES-For Submission/1. MAIN MANUSCRIPT IMAGES/Daoy-EX/3C.D-EX-HSPA6-G.tif]

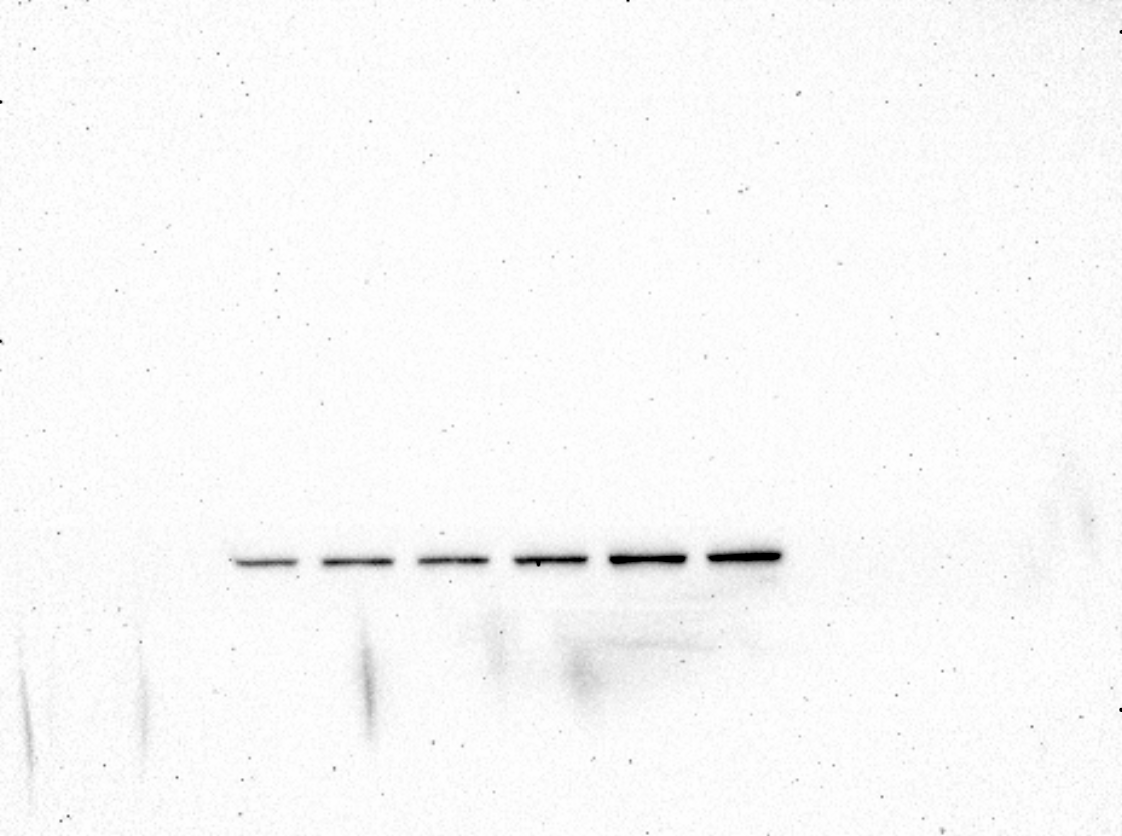

Supplement: Supplementary file 1 [file biology-12-00416-s001.zip › biology-2248156-supplementary-final/File S1-WB IMAGES-For Submission/1. MAIN MANUSCRIPT IMAGES/Daoy-EX/4.D-EX-HSPA4L.tif]

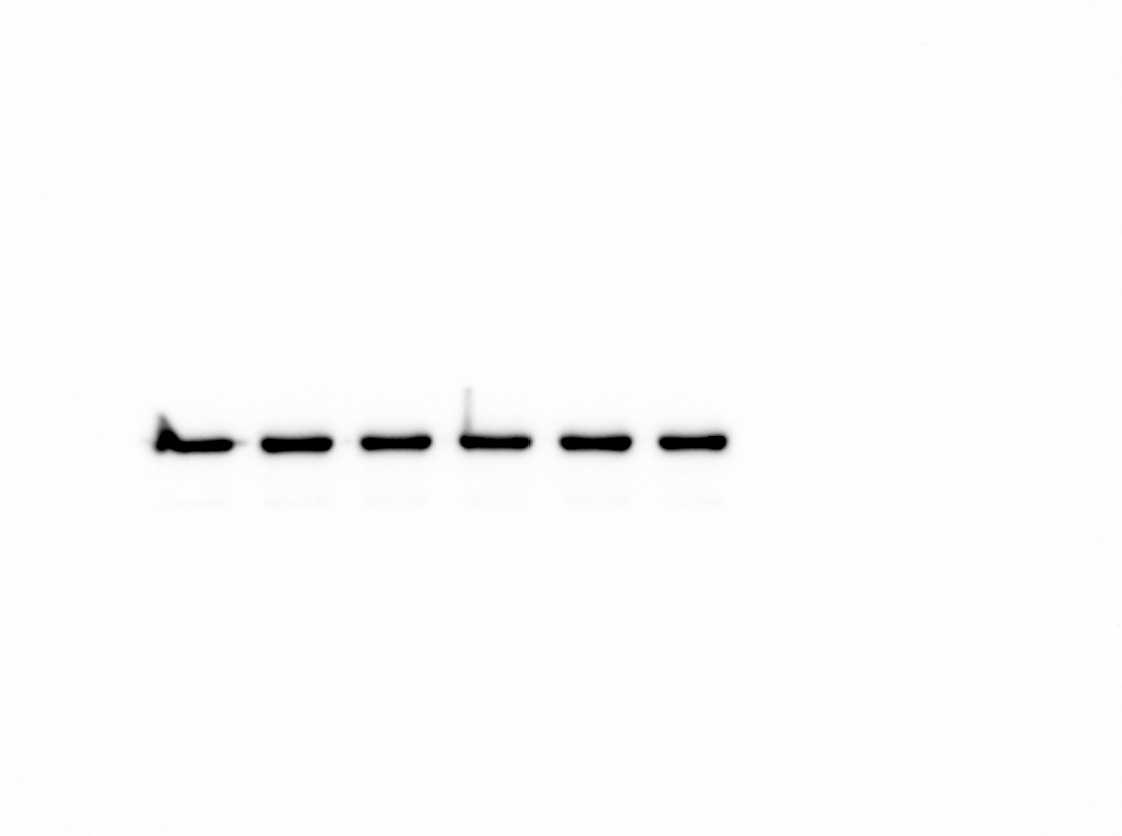

Supplement: Supplementary file 1 [file biology-12-00416-s001.zip › biology-2248156-supplementary-final/File S1-WB IMAGES-For Submission/1. MAIN MANUSCRIPT IMAGES/Daoy-EX/4C.D-EX-HSPA4L-G.tif]

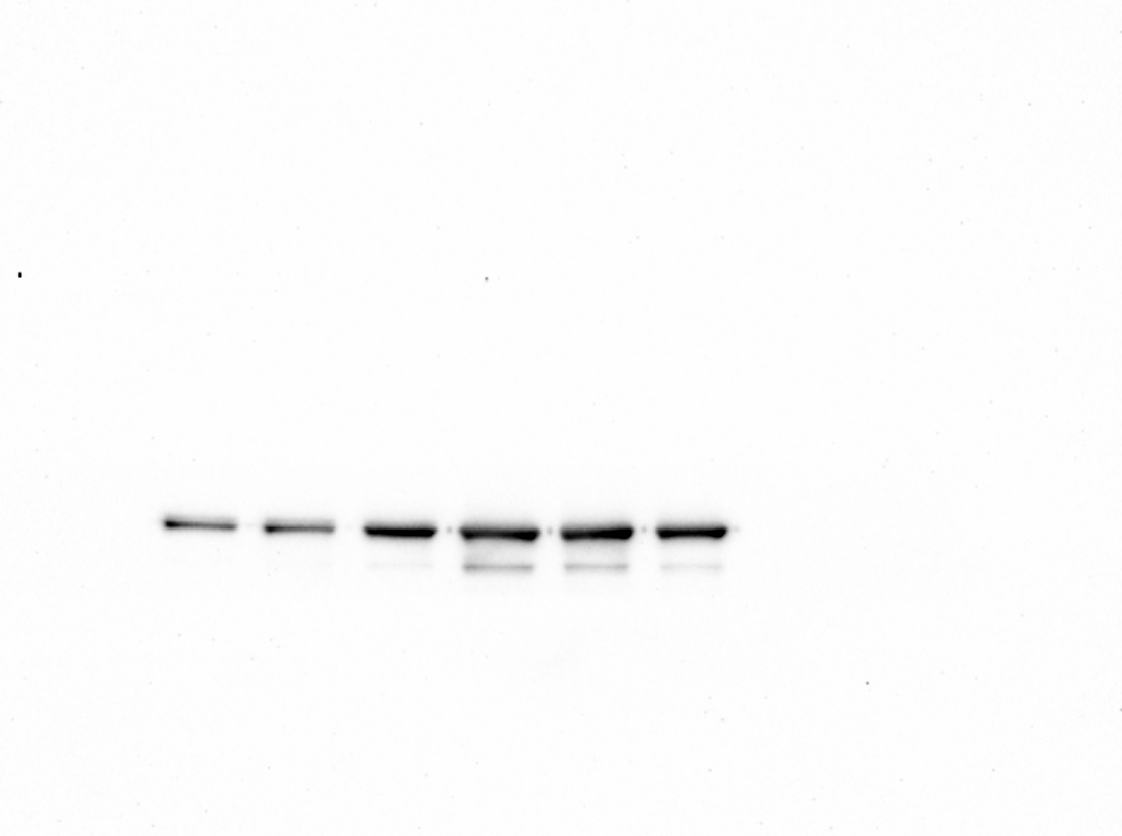

Supplement: Supplementary file 1 [file biology-12-00416-s001.zip › biology-2248156-supplementary-final/File S1-WB IMAGES-For Submission/1. MAIN MANUSCRIPT IMAGES/Daoy-MI/1.D-MI-HSPA1A.tif]

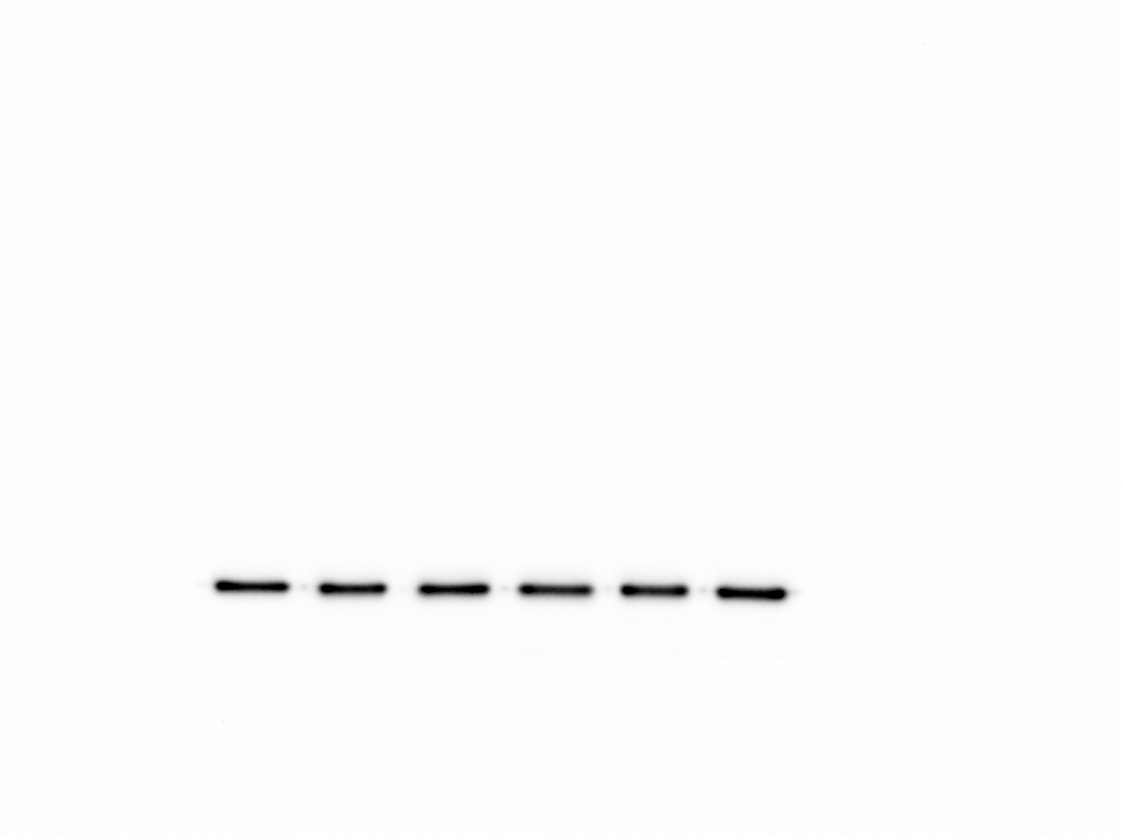

Supplement: Supplementary file 1 [file biology-12-00416-s001.zip › biology-2248156-supplementary-final/File S1-WB IMAGES-For Submission/1. MAIN MANUSCRIPT IMAGES/Daoy-MI/1C.D-MI-HSPA1A-G.tif]

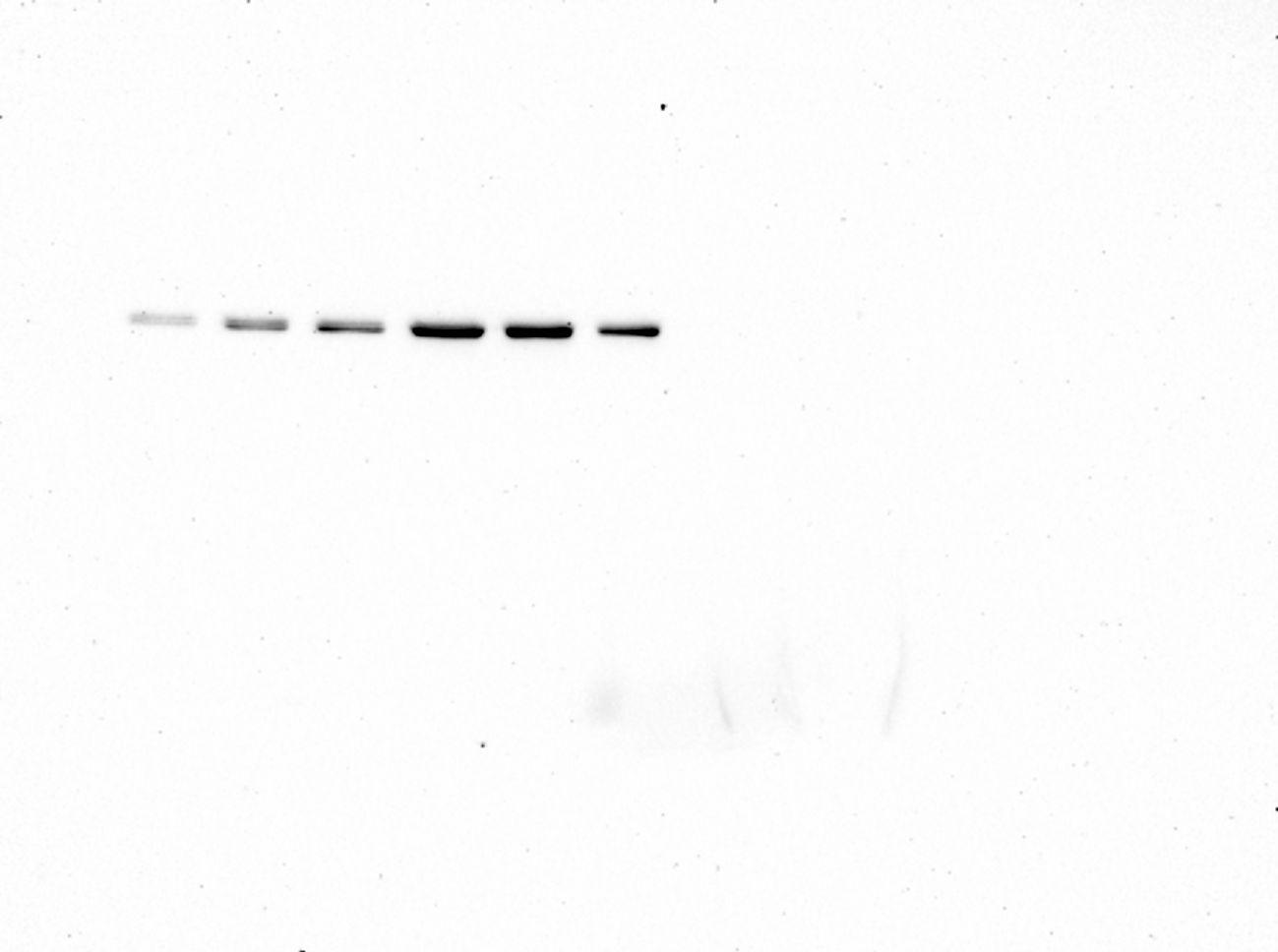

Supplement: Supplementary file 1 [file biology-12-00416-s001.zip › biology-2248156-supplementary-final/File S1-WB IMAGES-For Submission/1. MAIN MANUSCRIPT IMAGES/Daoy-MI/2.D-MI-HSPA1B.tif]

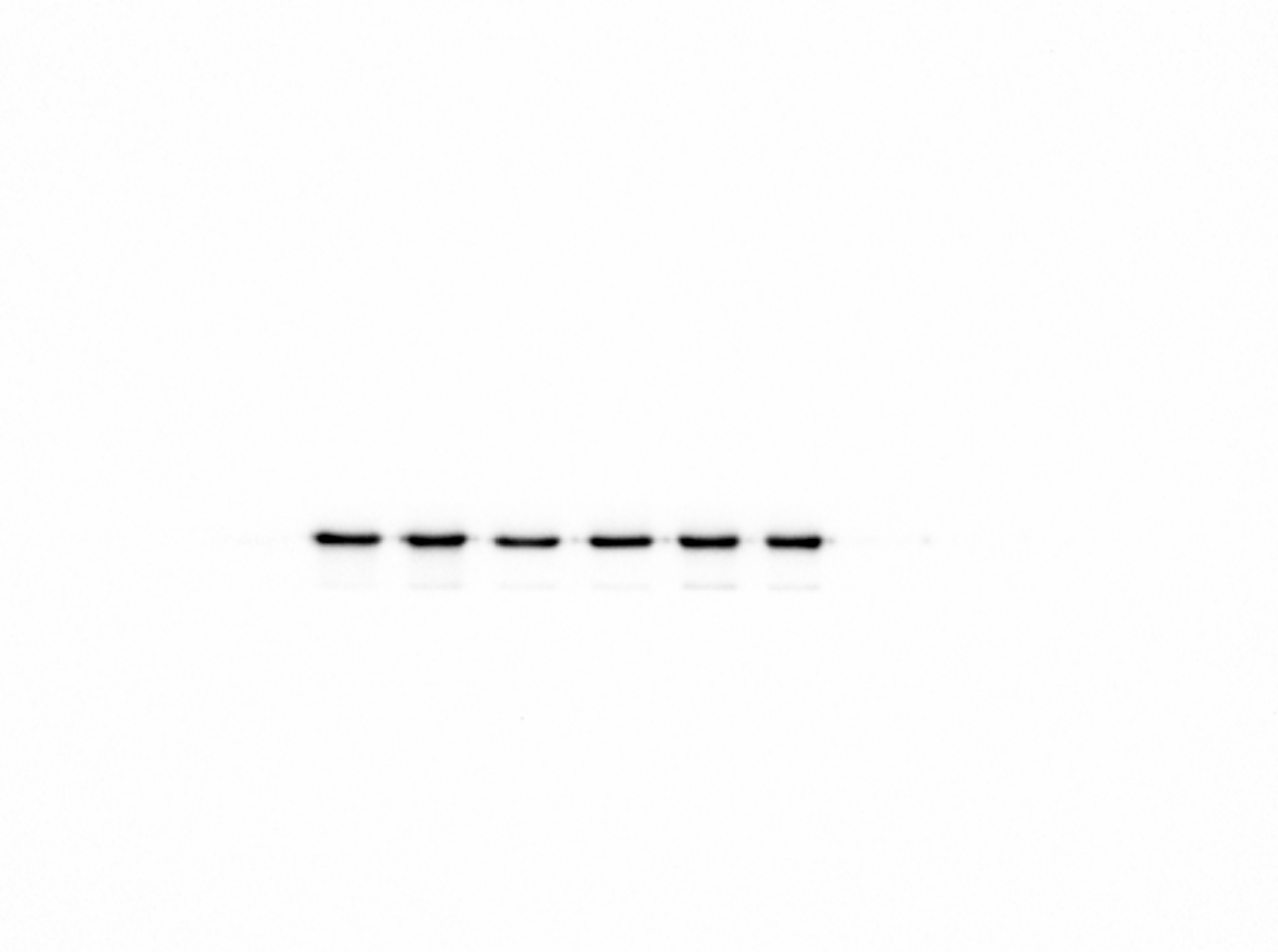

Supplement: Supplementary file 1 [file biology-12-00416-s001.zip › biology-2248156-supplementary-final/File S1-WB IMAGES-For Submission/1. MAIN MANUSCRIPT IMAGES/Daoy-MI/2C.D-MI-HSPA1B-G.tif]

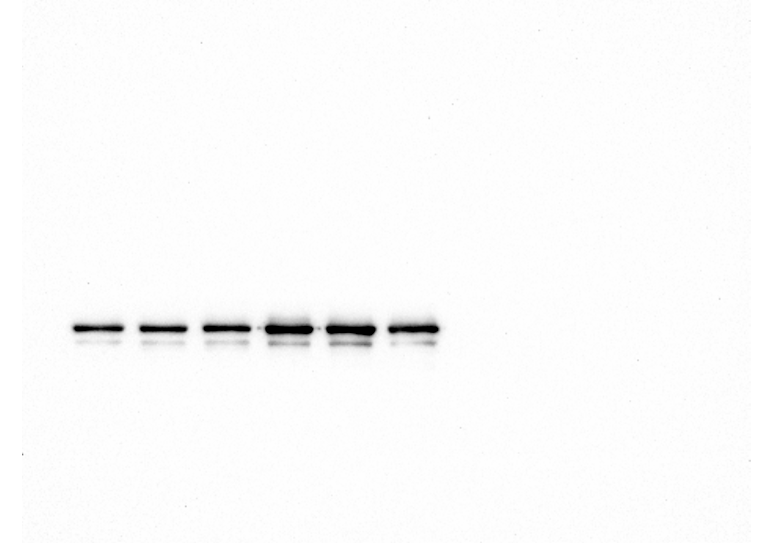

Supplement: Supplementary file 1 [file biology-12-00416-s001.zip › biology-2248156-supplementary-final/File S1-WB IMAGES-For Submission/1. MAIN MANUSCRIPT IMAGES/SH(D)-EX/1.SH(D)-EX-HSPA1A.tif]

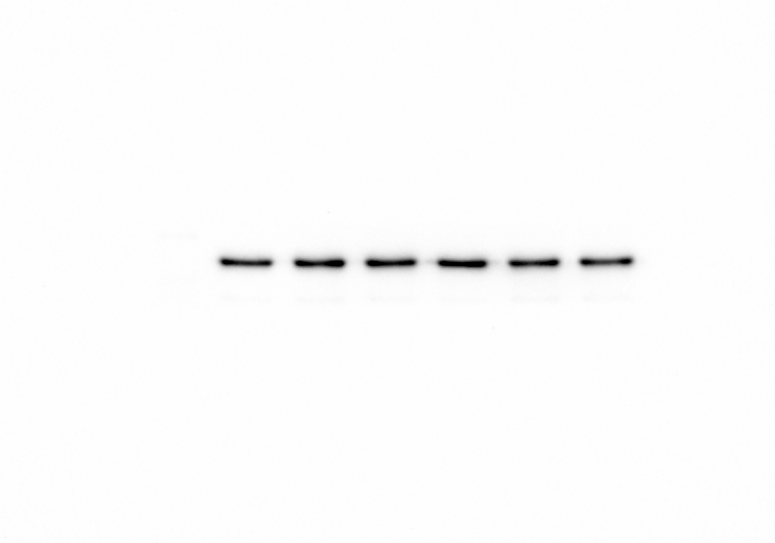

Supplement: Supplementary file 1 [file biology-12-00416-s001.zip › biology-2248156-supplementary-final/File S1-WB IMAGES-For Submission/1. MAIN MANUSCRIPT IMAGES/SH(D)-EX/1C.SH(D)-EX-HSPA1A-G.tif]

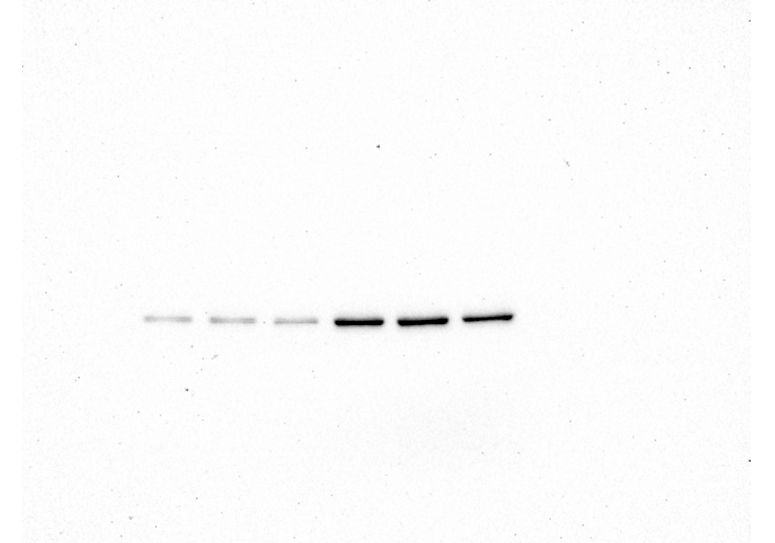

Supplement: Supplementary file 1 [file biology-12-00416-s001.zip › biology-2248156-supplementary-final/File S1-WB IMAGES-For Submission/1. MAIN MANUSCRIPT IMAGES/SH(D)-EX/2.SH(D)-EX-HSPA1B.tif]

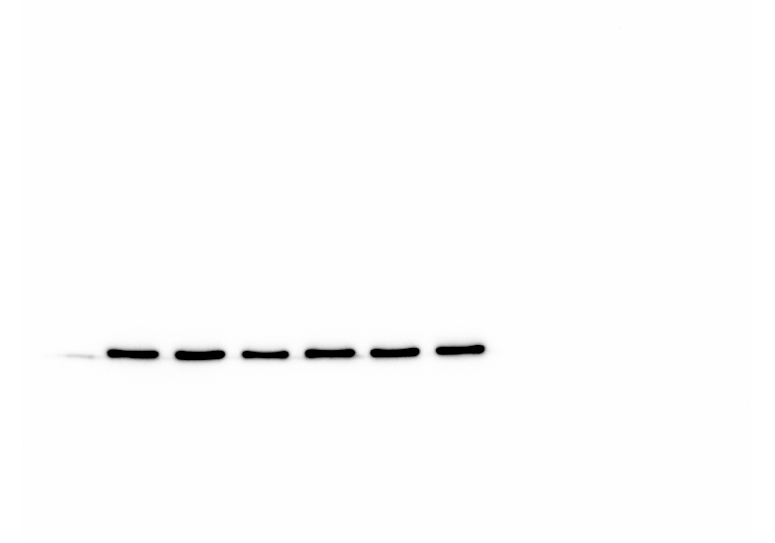

Supplement: Supplementary file 1 [file biology-12-00416-s001.zip › biology-2248156-supplementary-final/File S1-WB IMAGES-For Submission/1. MAIN MANUSCRIPT IMAGES/SH(D)-EX/2C.SH(D)-EX-HSPA1B-G.tif]

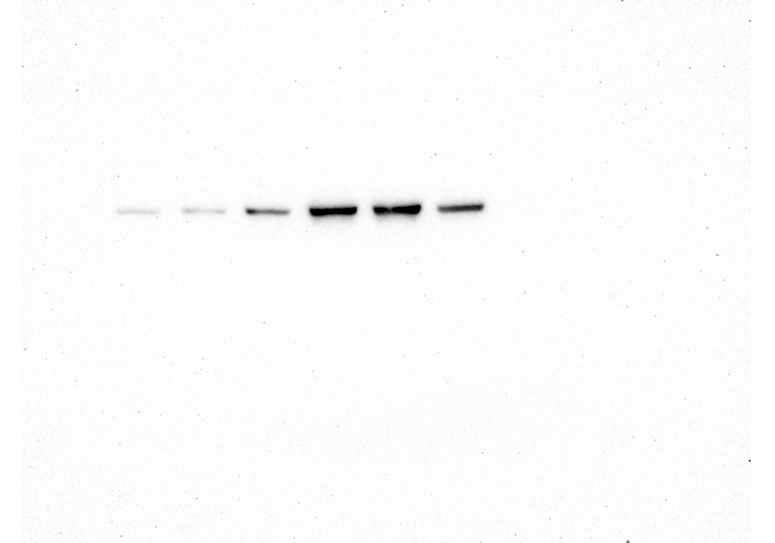

Supplement: Supplementary file 1 [file biology-12-00416-s001.zip › biology-2248156-supplementary-final/File S1-WB IMAGES-For Submission/1. MAIN MANUSCRIPT IMAGES/SH(D)-EX/3.SH(D)-EX-HSPA6.tif]

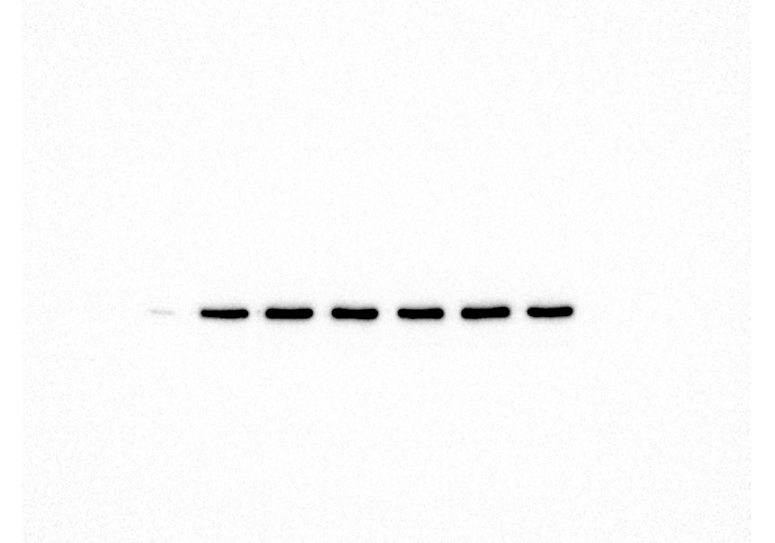

Supplement: Supplementary file 1 [file biology-12-00416-s001.zip › biology-2248156-supplementary-final/File S1-WB IMAGES-For Submission/1. MAIN MANUSCRIPT IMAGES/SH(D)-EX/3C.SH(D)-EX-HSPA6-G.tif]

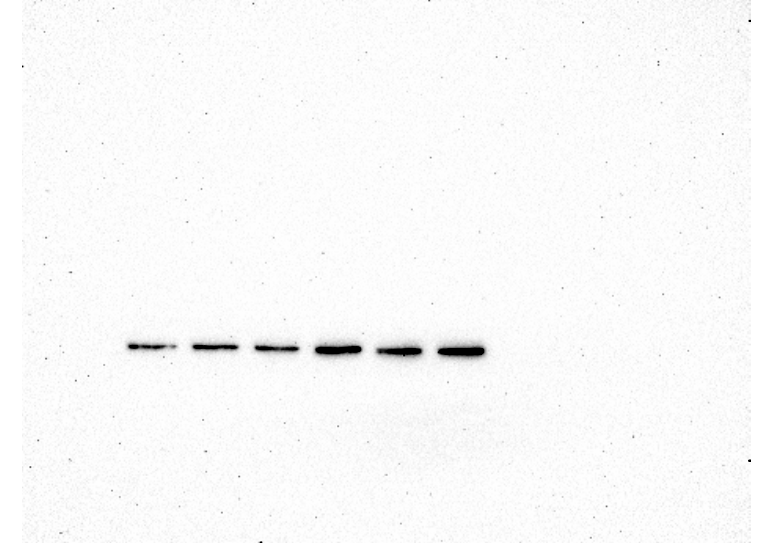

Supplement: Supplementary file 1 [file biology-12-00416-s001.zip › biology-2248156-supplementary-final/File S1-WB IMAGES-For Submission/1. MAIN MANUSCRIPT IMAGES/SH(D)-EX/4.SH(D)-EX-HSPA1L.tif]

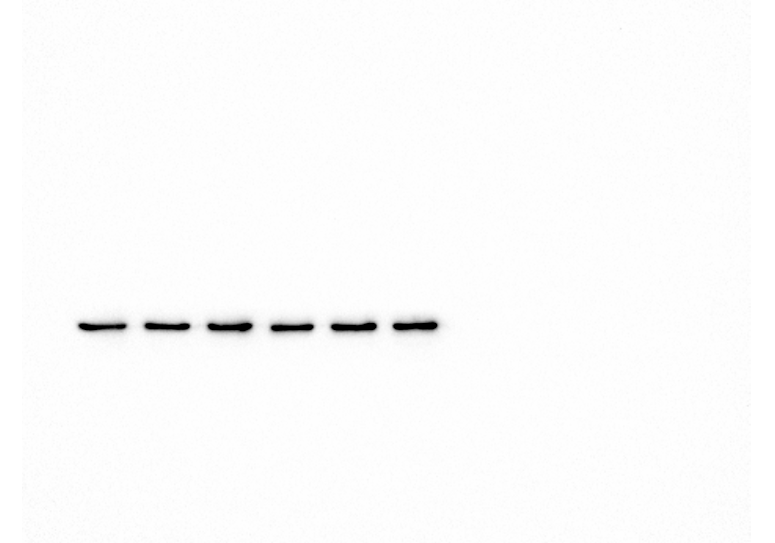

Supplement: Supplementary file 1 [file biology-12-00416-s001.zip › biology-2248156-supplementary-final/File S1-WB IMAGES-For Submission/1. MAIN MANUSCRIPT IMAGES/SH(D)-EX/4C.SH(D)-EX-HSPH1-G.tif]

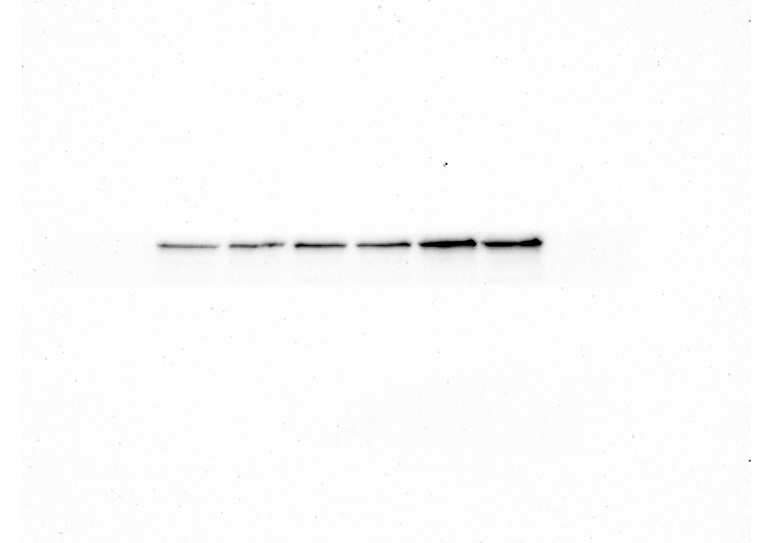

Supplement: Supplementary file 1 [file biology-12-00416-s001.zip › biology-2248156-supplementary-final/File S1-WB IMAGES-For Submission/1. MAIN MANUSCRIPT IMAGES/SH(D)-EX/5.SH(D)-EX-HSPA4L.tif]

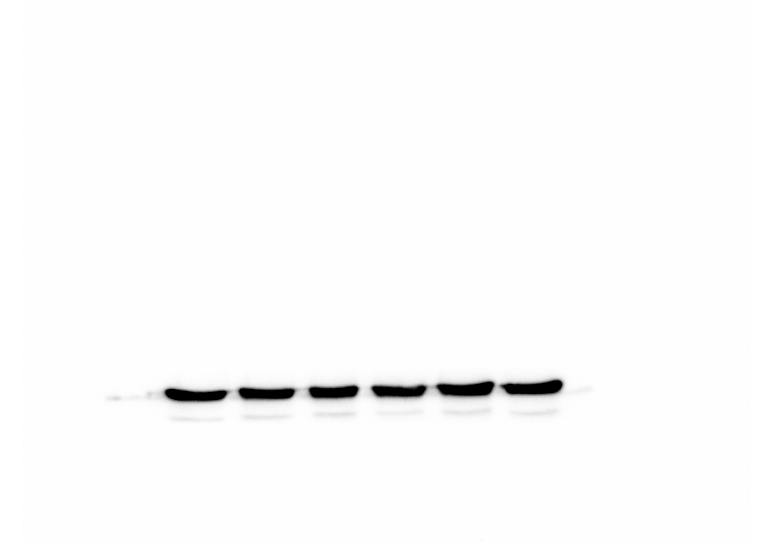

Supplement: Supplementary file 1 [file biology-12-00416-s001.zip › biology-2248156-supplementary-final/File S1-WB IMAGES-For Submission/1. MAIN MANUSCRIPT IMAGES/SH(D)-EX/5C.SH(D)-EX-HSPA4L-G.tif]

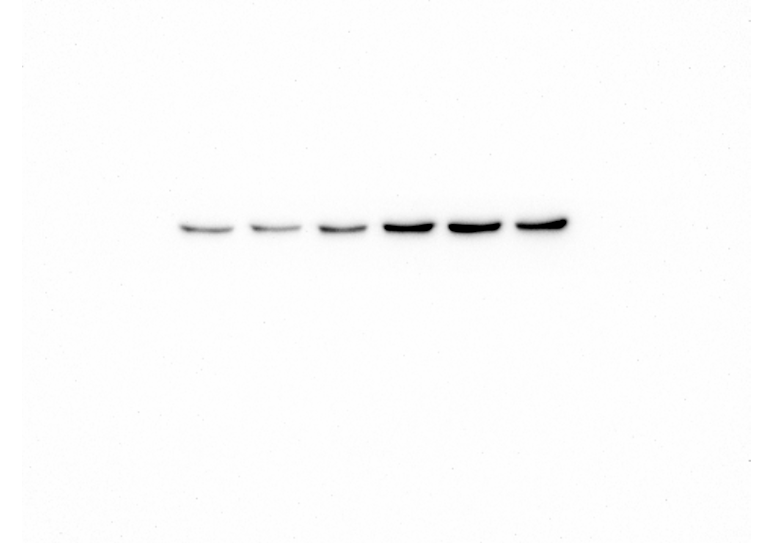

Supplement: Supplementary file 1 [file biology-12-00416-s001.zip › biology-2248156-supplementary-final/File S1-WB IMAGES-For Submission/1. MAIN MANUSCRIPT IMAGES/SH(D)-EX/6.SH(D)-EX-DNAJB1.tif]

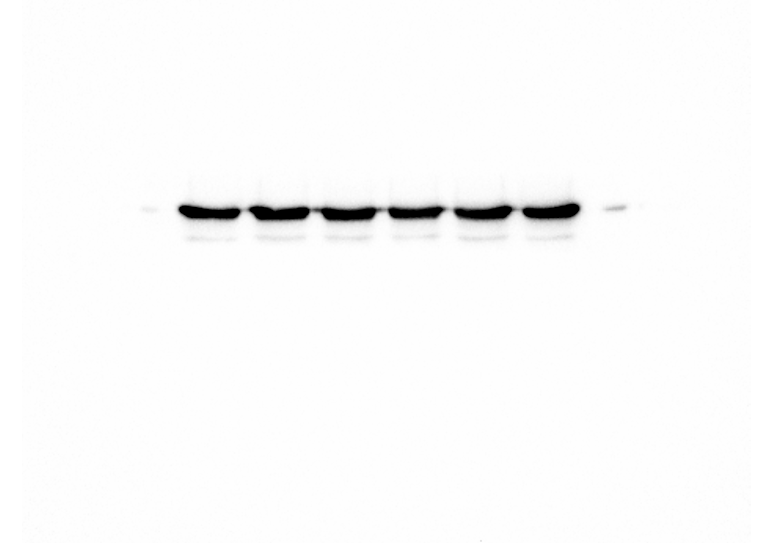

Supplement: Supplementary file 1 [file biology-12-00416-s001.zip › biology-2248156-supplementary-final/File S1-WB IMAGES-For Submission/1. MAIN MANUSCRIPT IMAGES/SH(D)-EX/6C.SH(D)-EX-DNAJB1-G.tif]

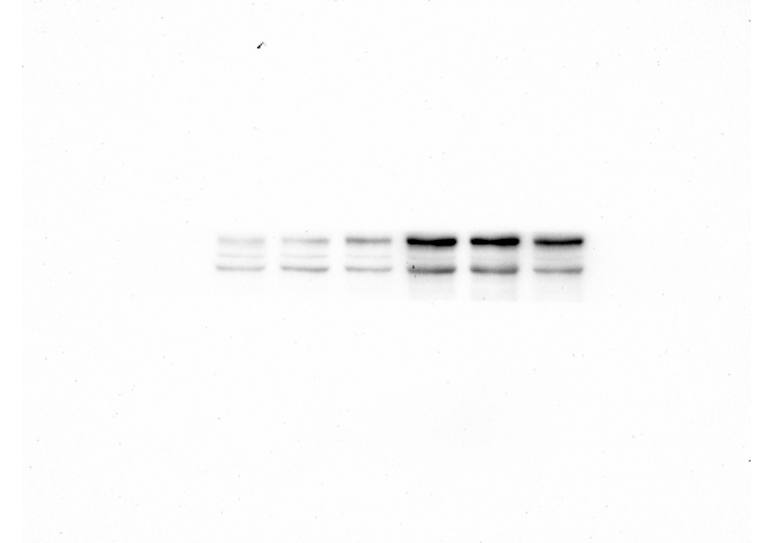

Supplement: Supplementary file 1 [file biology-12-00416-s001.zip › biology-2248156-supplementary-final/File S1-WB IMAGES-For Submission/1. MAIN MANUSCRIPT IMAGES/SH(D)-EX/7.SH(D)-EX-BAG3.tif]

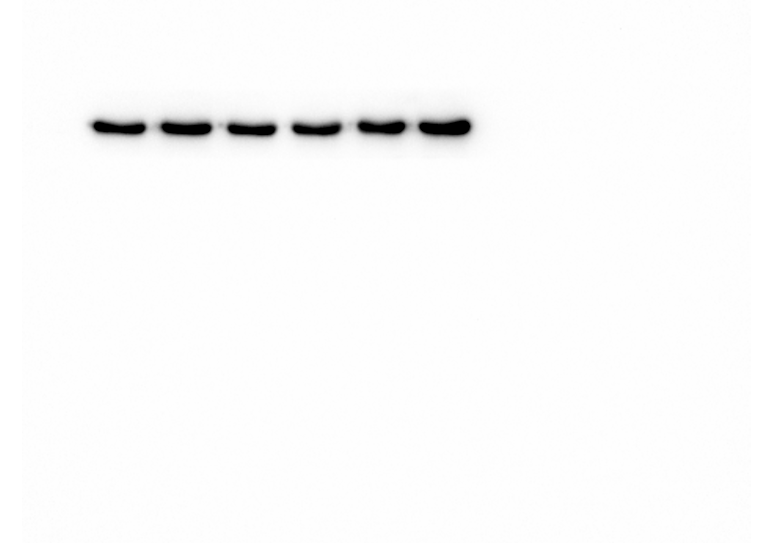

Supplement: Supplementary file 1 [file biology-12-00416-s001.zip › biology-2248156-supplementary-final/File S1-WB IMAGES-For Submission/1. MAIN MANUSCRIPT IMAGES/SH(D)-EX/7C.SH(D)-EX-BAG3-G.tif]

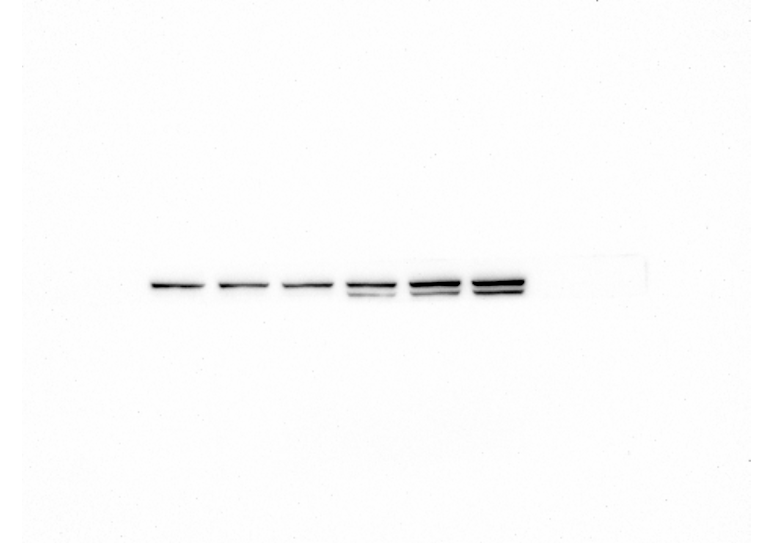

Supplement: Supplementary file 1 [file biology-12-00416-s001.zip › biology-2248156-supplementary-final/File S1-WB IMAGES-For Submission/1. MAIN MANUSCRIPT IMAGES/SH(D)-EX/8.SH(D)-EX-HSPH1.tif]

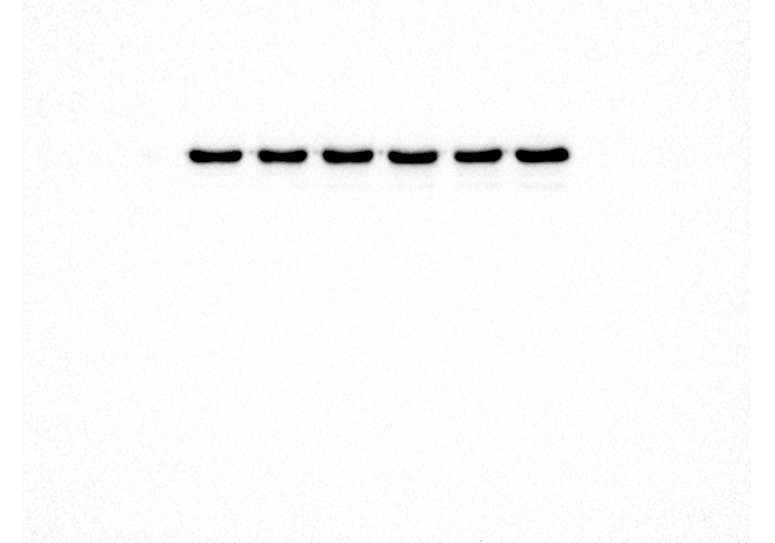

Supplement: Supplementary file 1 [file biology-12-00416-s001.zip › biology-2248156-supplementary-final/File S1-WB IMAGES-For Submission/1. MAIN MANUSCRIPT IMAGES/SH(D)-EX/8C.SH(D)-EX-HSPH1-G.tif]

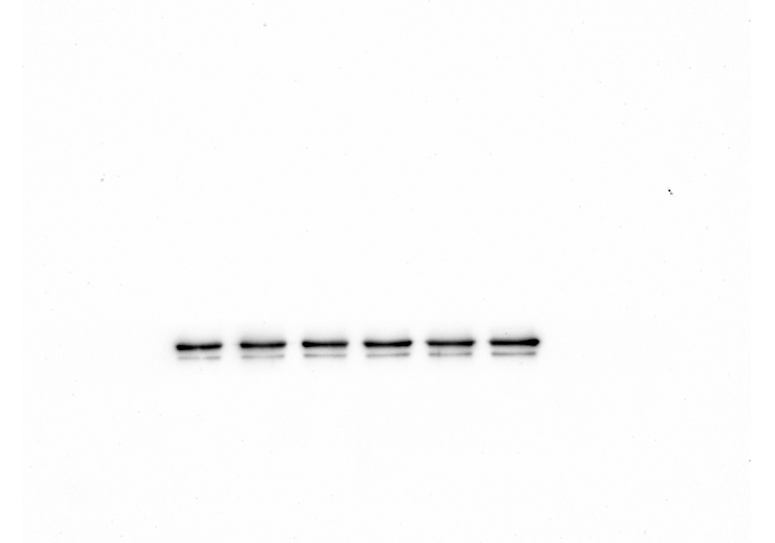

Supplement: Supplementary file 1 [file biology-12-00416-s001.zip › biology-2248156-supplementary-final/File S1-WB IMAGES-For Submission/1. MAIN MANUSCRIPT IMAGES/SH(D)-MI/1.SH(D)-MI-HSPA1A.tif]

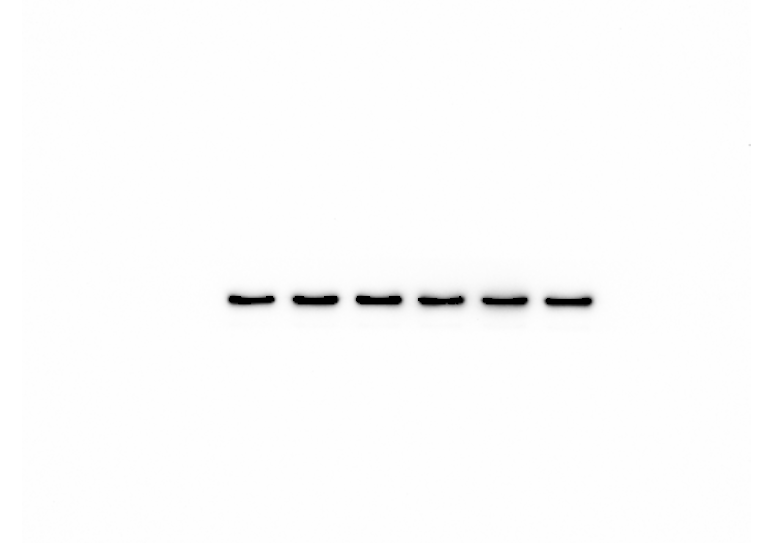

Supplement: Supplementary file 1 [file biology-12-00416-s001.zip › biology-2248156-supplementary-final/File S1-WB IMAGES-For Submission/1. MAIN MANUSCRIPT IMAGES/SH(D)-MI/1C.SH(D)-MI-HSPA1A-G.tif]

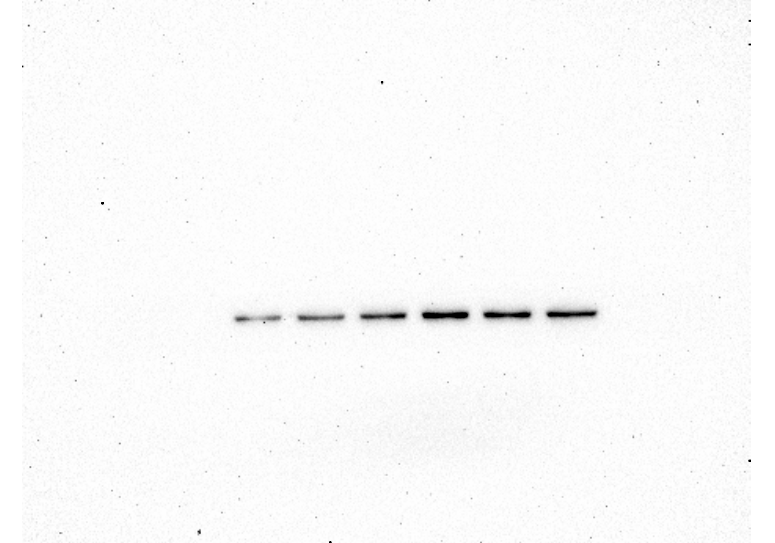

Supplement: Supplementary file 1 [file biology-12-00416-s001.zip › biology-2248156-supplementary-final/File S1-WB IMAGES-For Submission/1. MAIN MANUSCRIPT IMAGES/SH(D)-MI/2.SH(D)-MI-HSPA1B.tif]

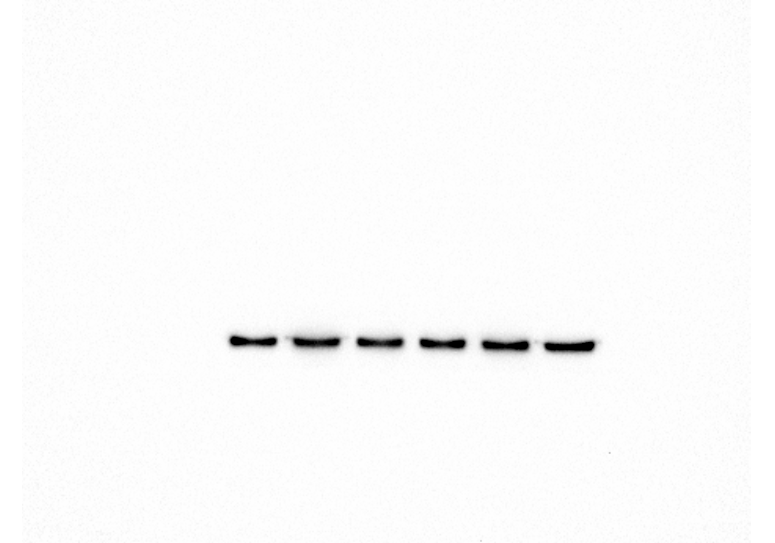

Supplement: Supplementary file 1 [file biology-12-00416-s001.zip › biology-2248156-supplementary-final/File S1-WB IMAGES-For Submission/1. MAIN MANUSCRIPT IMAGES/SH(D)-MI/2C.SH(D)-MI-HSPA1B-G.tif]

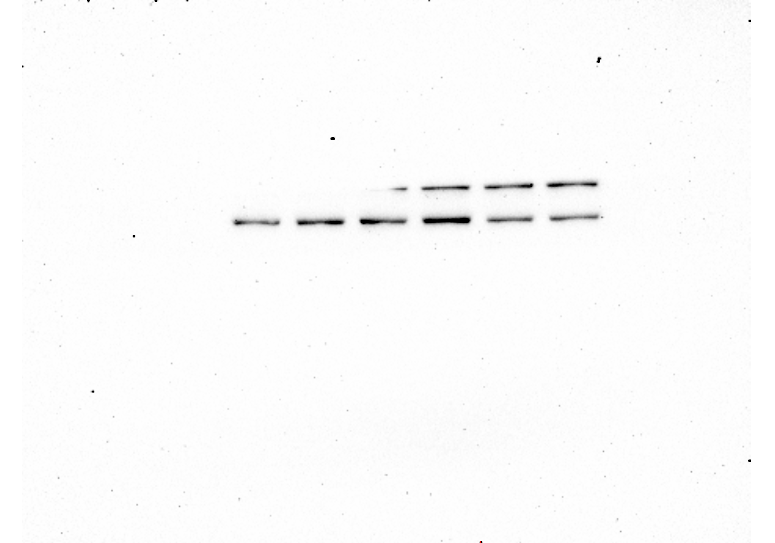

Supplement: Supplementary file 1 [file biology-12-00416-s001.zip › biology-2248156-supplementary-final/File S1-WB IMAGES-For Submission/1. MAIN MANUSCRIPT IMAGES/SH(D)-MI/3.SH(D)-MI-HSPA6.tif]

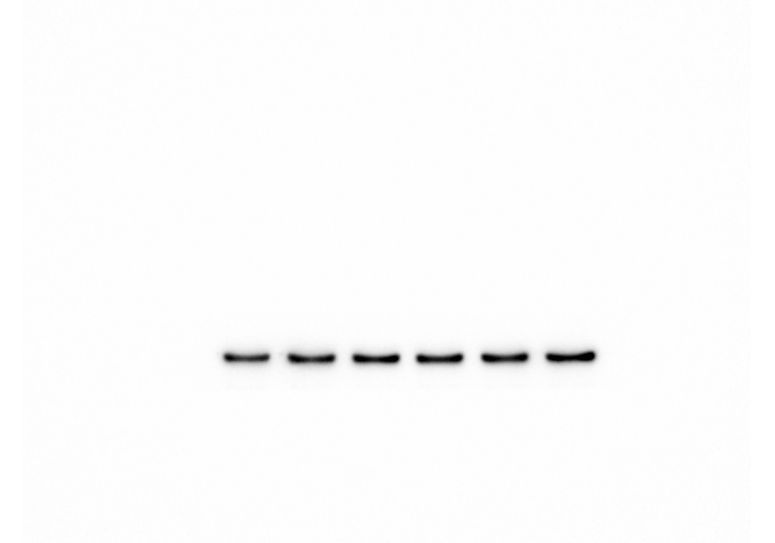

Supplement: Supplementary file 1 [file biology-12-00416-s001.zip › biology-2248156-supplementary-final/File S1-WB IMAGES-For Submission/1. MAIN MANUSCRIPT IMAGES/SH(D)-MI/3C.SH(D)-MI-HSPA6-G.tif]

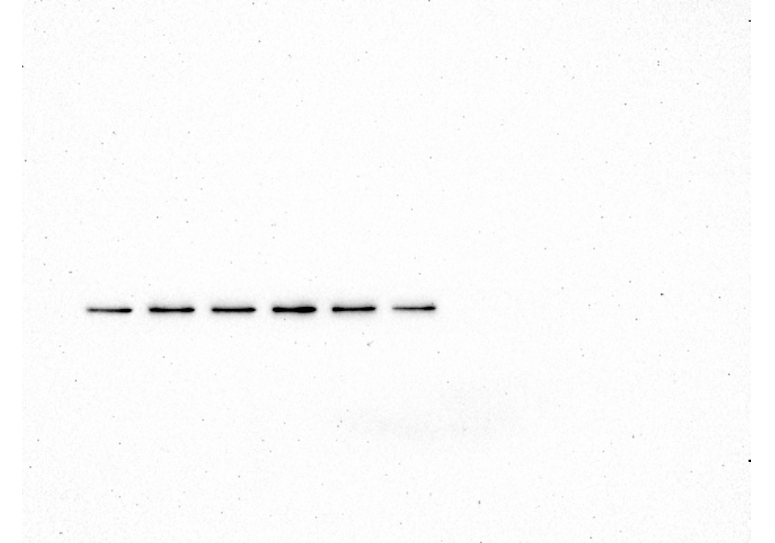

Supplement: Supplementary file 1 [file biology-12-00416-s001.zip › biology-2248156-supplementary-final/File S1-WB IMAGES-For Submission/1. MAIN MANUSCRIPT IMAGES/SH(D)-MI/4.SH(D)-MI-HSPA1L.tif]

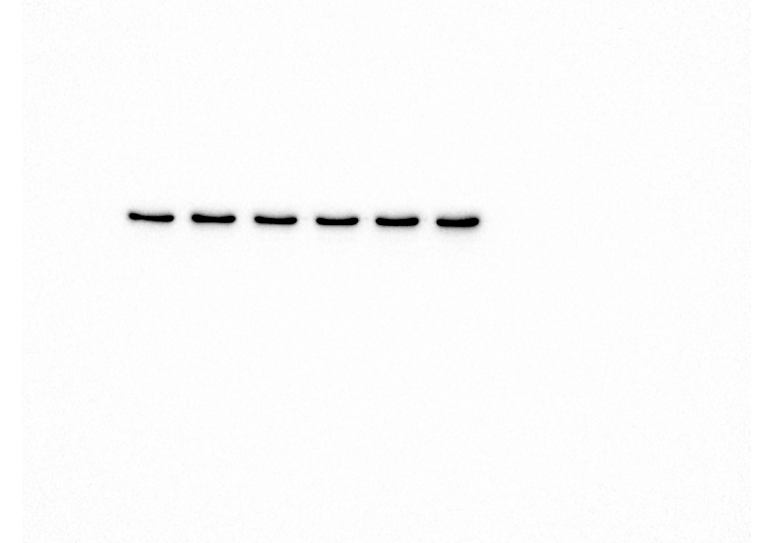

Supplement: Supplementary file 1 [file biology-12-00416-s001.zip › biology-2248156-supplementary-final/File S1-WB IMAGES-For Submission/1. MAIN MANUSCRIPT IMAGES/SH(D)-MI/4C.SH(D)-MI-HSPA1L-G.tif]

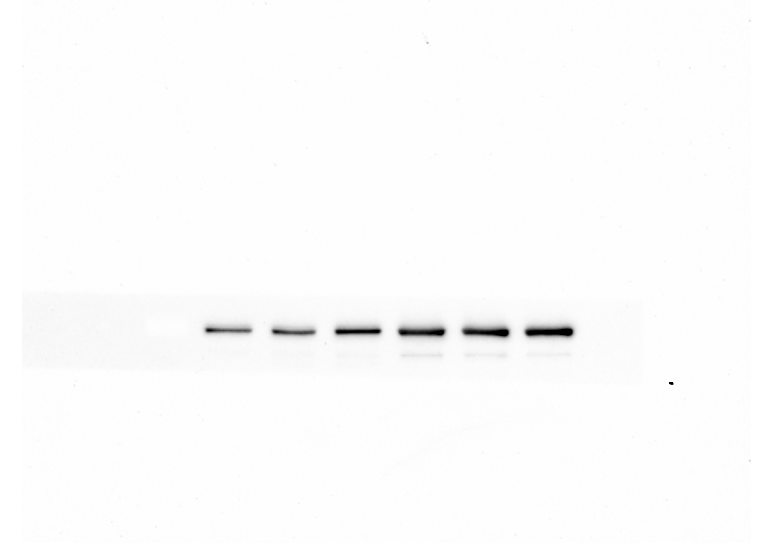

Supplement: Supplementary file 1 [file biology-12-00416-s001.zip › biology-2248156-supplementary-final/File S1-WB IMAGES-For Submission/1. MAIN MANUSCRIPT IMAGES/SH-EX/1.SH-EX-HSPA1A.tif]

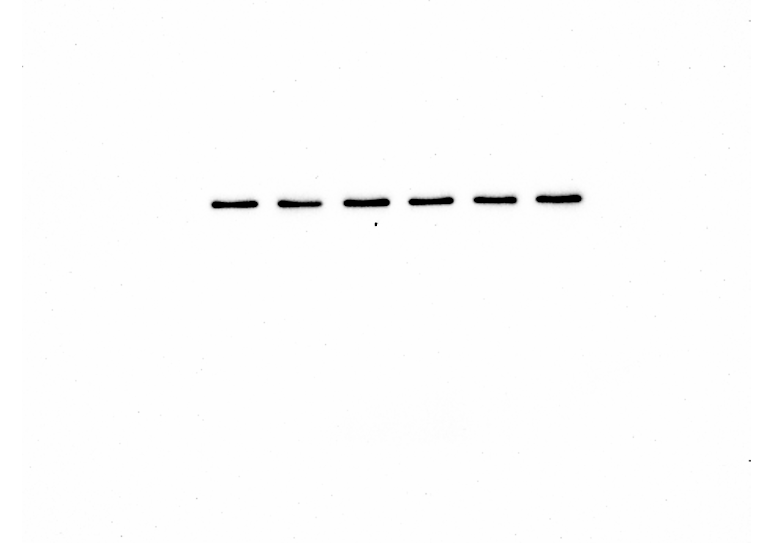

Supplement: Supplementary file 1 [file biology-12-00416-s001.zip › biology-2248156-supplementary-final/File S1-WB IMAGES-For Submission/1. MAIN MANUSCRIPT IMAGES/SH-EX/1C.SH-EX-HSPA1A-G.tif]

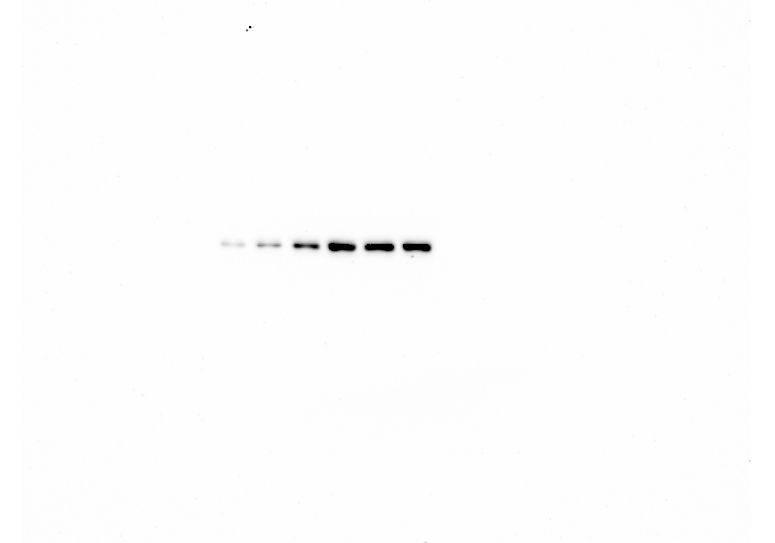

Supplement: Supplementary file 1 [file biology-12-00416-s001.zip › biology-2248156-supplementary-final/File S1-WB IMAGES-For Submission/1. MAIN MANUSCRIPT IMAGES/SH-EX/2.SH-EX-HSPA1B.tif]

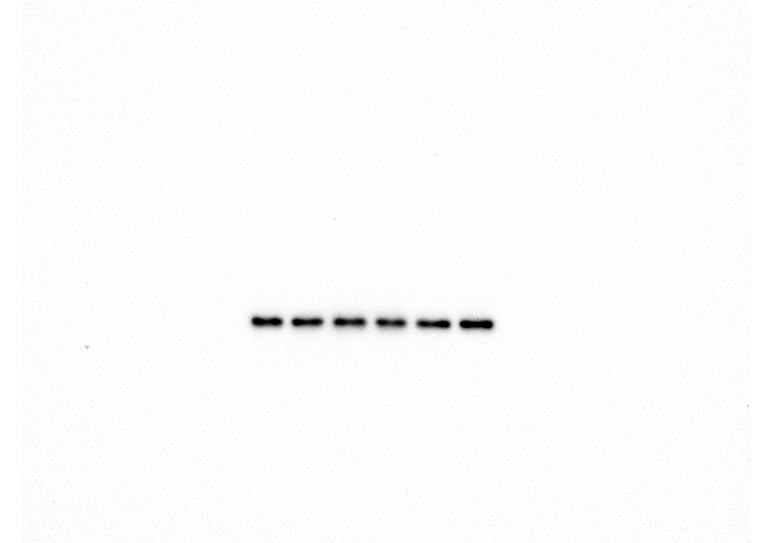

Supplement: Supplementary file 1 [file biology-12-00416-s001.zip › biology-2248156-supplementary-final/File S1-WB IMAGES-For Submission/1. MAIN MANUSCRIPT IMAGES/SH-EX/2C.SH-EX-HSPA1B-G.tif]

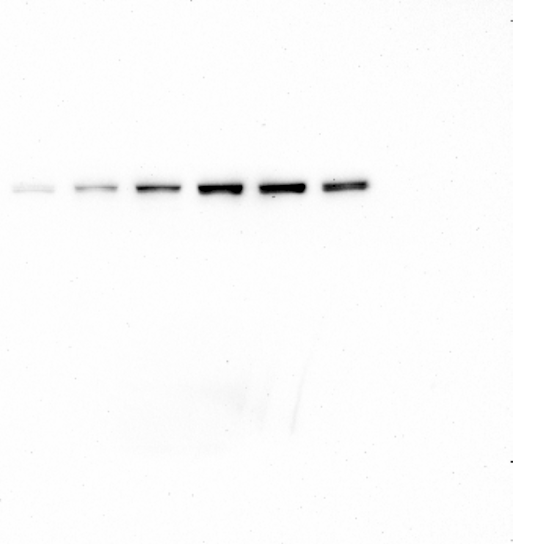

Supplement: Supplementary file 1 [file biology-12-00416-s001.zip › biology-2248156-supplementary-final/File S1-WB IMAGES-For Submission/1. MAIN MANUSCRIPT IMAGES/SH-EX/3.SH-EX-HSPA6.tif]

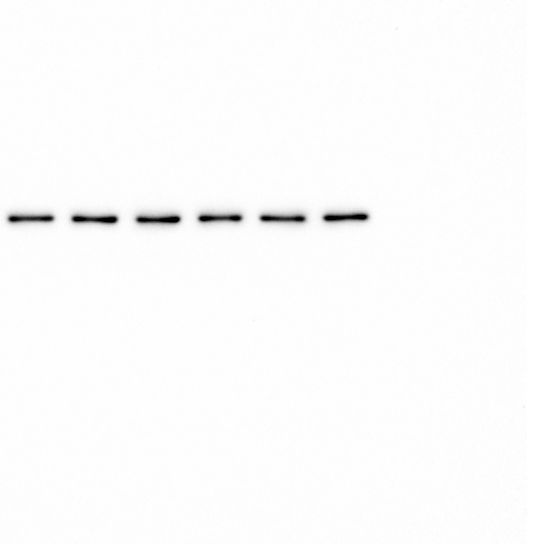

Supplement: Supplementary file 1 [file biology-12-00416-s001.zip › biology-2248156-supplementary-final/File S1-WB IMAGES-For Submission/1. MAIN MANUSCRIPT IMAGES/SH-EX/3C.SH-EX-HSPA6-G.tif]

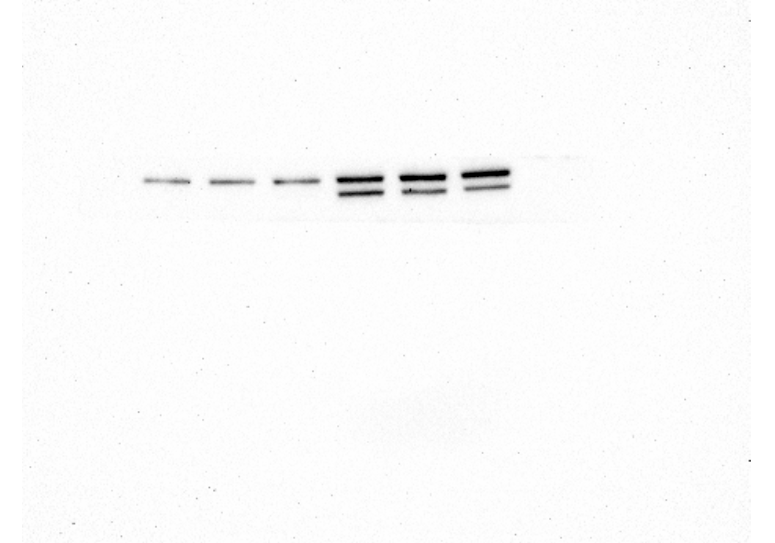

Supplement: Supplementary file 1 [file biology-12-00416-s001.zip › biology-2248156-supplementary-final/File S1-WB IMAGES-For Submission/1. MAIN MANUSCRIPT IMAGES/SH-EX/4.SH-EX-HSPH1.tif]

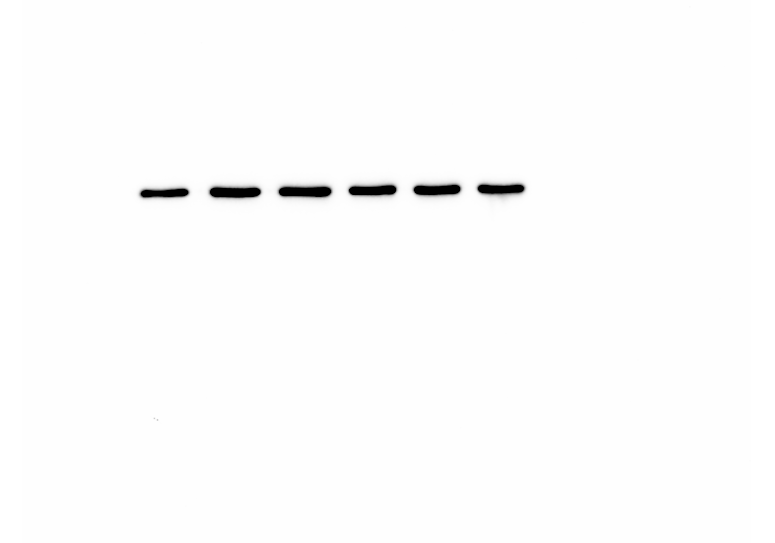

Supplement: Supplementary file 1 [file biology-12-00416-s001.zip › biology-2248156-supplementary-final/File S1-WB IMAGES-For Submission/1. MAIN MANUSCRIPT IMAGES/SH-EX/4C.SH-EX-HSPH1-G.tif]

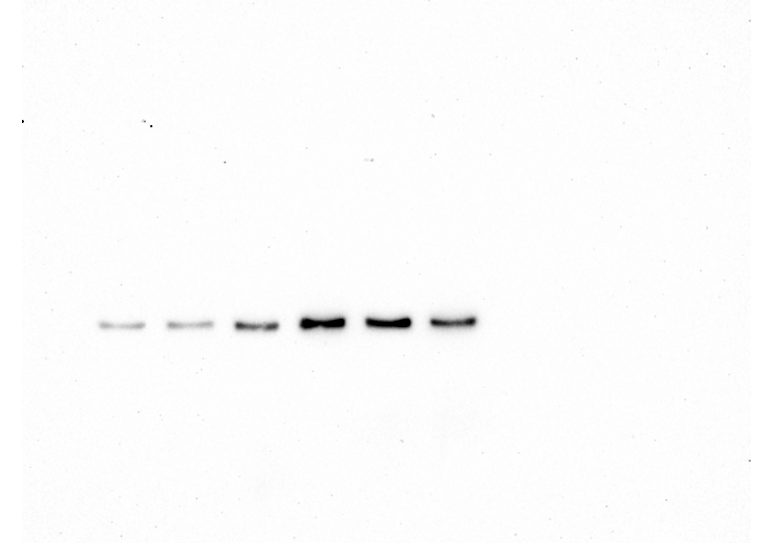

Supplement: Supplementary file 1 [file biology-12-00416-s001.zip › biology-2248156-supplementary-final/File S1-WB IMAGES-For Submission/1. MAIN MANUSCRIPT IMAGES/SH-EX/5.SH-EX-DNAJB1.tif]

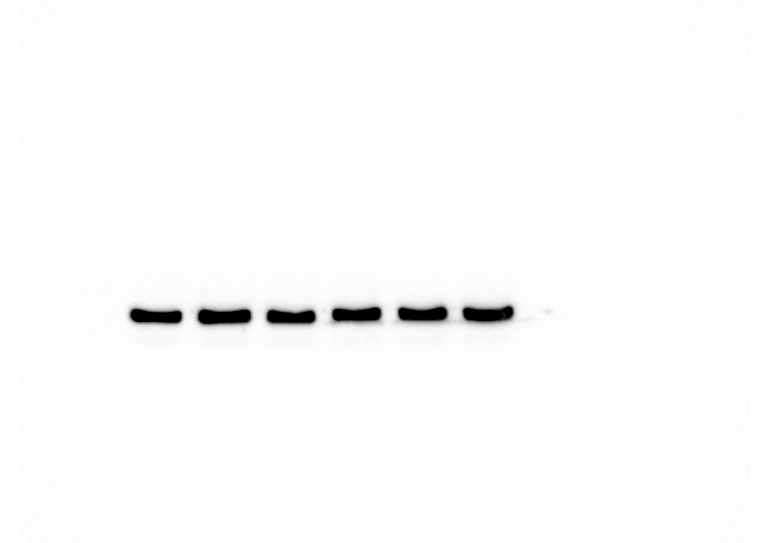

Supplement: Supplementary file 1 [file biology-12-00416-s001.zip › biology-2248156-supplementary-final/File S1-WB IMAGES-For Submission/1. MAIN MANUSCRIPT IMAGES/SH-EX/5C.SH-EX-DNAJB1-G.tif]

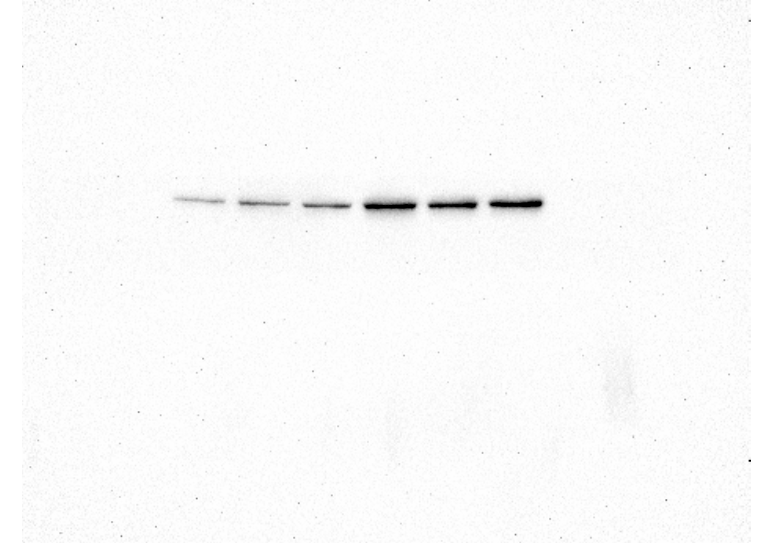

Supplement: Supplementary file 1 [file biology-12-00416-s001.zip › biology-2248156-supplementary-final/File S1-WB IMAGES-For Submission/1. MAIN MANUSCRIPT IMAGES/SH-EX/6.SH-EX-HSPA4L.tif]

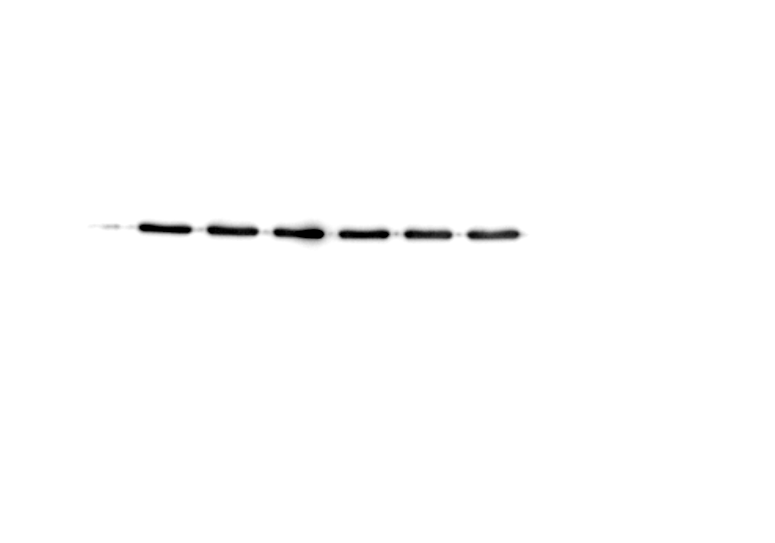

Supplement: Supplementary file 1 [file biology-12-00416-s001.zip › biology-2248156-supplementary-final/File S1-WB IMAGES-For Submission/1. MAIN MANUSCRIPT IMAGES/SH-EX/6C.SH-EX-HSPA4L-G.tif]

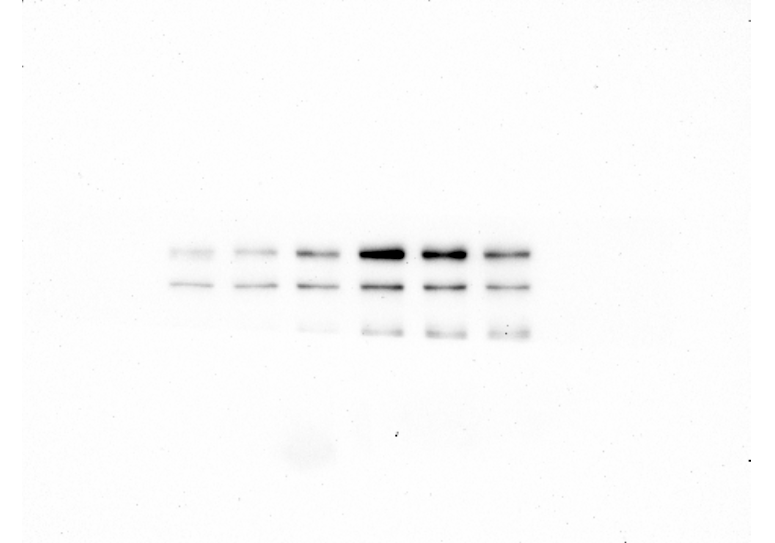

Supplement: Supplementary file 1 [file biology-12-00416-s001.zip › biology-2248156-supplementary-final/File S1-WB IMAGES-For Submission/1. MAIN MANUSCRIPT IMAGES/SH-EX/7.SH-EX-BAG3.tif]

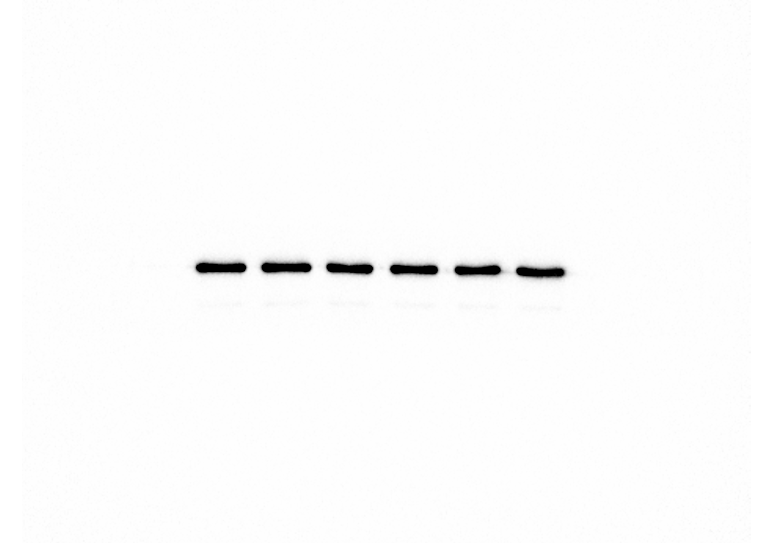

Supplement: Supplementary file 1 [file biology-12-00416-s001.zip › biology-2248156-supplementary-final/File S1-WB IMAGES-For Submission/1. MAIN MANUSCRIPT IMAGES/SH-EX/7C.SH-EX-BAG3-G.tif]

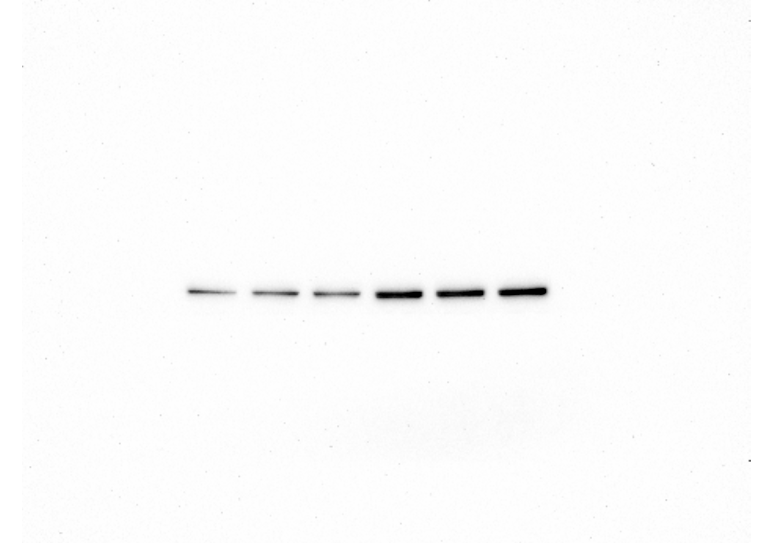

Supplement: Supplementary file 1 [file biology-12-00416-s001.zip › biology-2248156-supplementary-final/File S1-WB IMAGES-For Submission/1. MAIN MANUSCRIPT IMAGES/SH-EX/8.SH-EX-HSPA1L.tif]

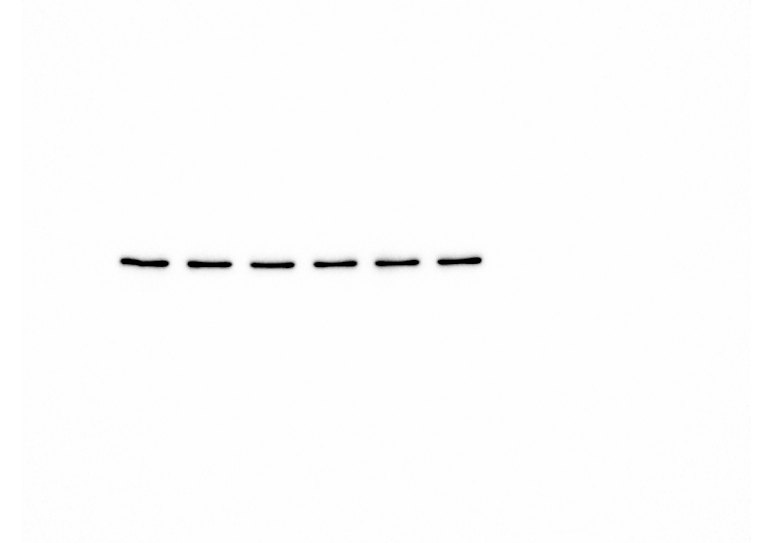

Supplement: Supplementary file 1 [file biology-12-00416-s001.zip › biology-2248156-supplementary-final/File S1-WB IMAGES-For Submission/1. MAIN MANUSCRIPT IMAGES/SH-EX/8C.SH-EX-HSPA1L-G.tif]

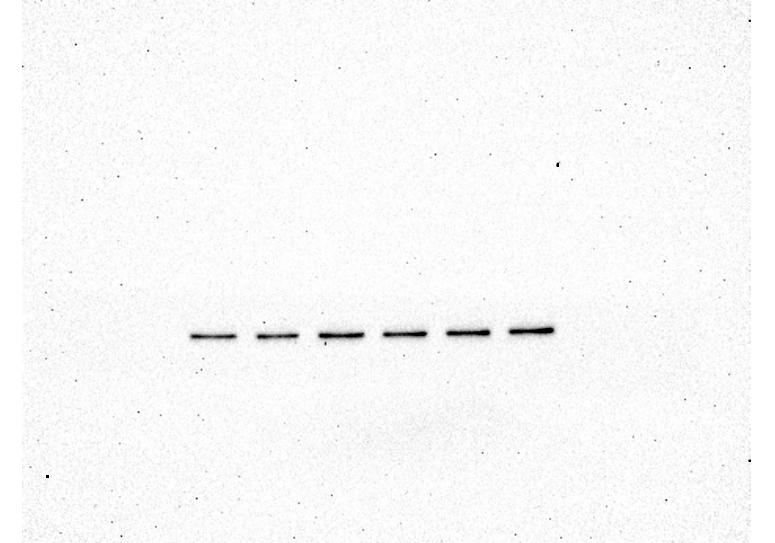

Supplement: Supplementary file 1 [file biology-12-00416-s001.zip › biology-2248156-supplementary-final/File S1-WB IMAGES-For Submission/1. MAIN MANUSCRIPT IMAGES/SH-MI/1.SH-MI-HSPA1A.tif]

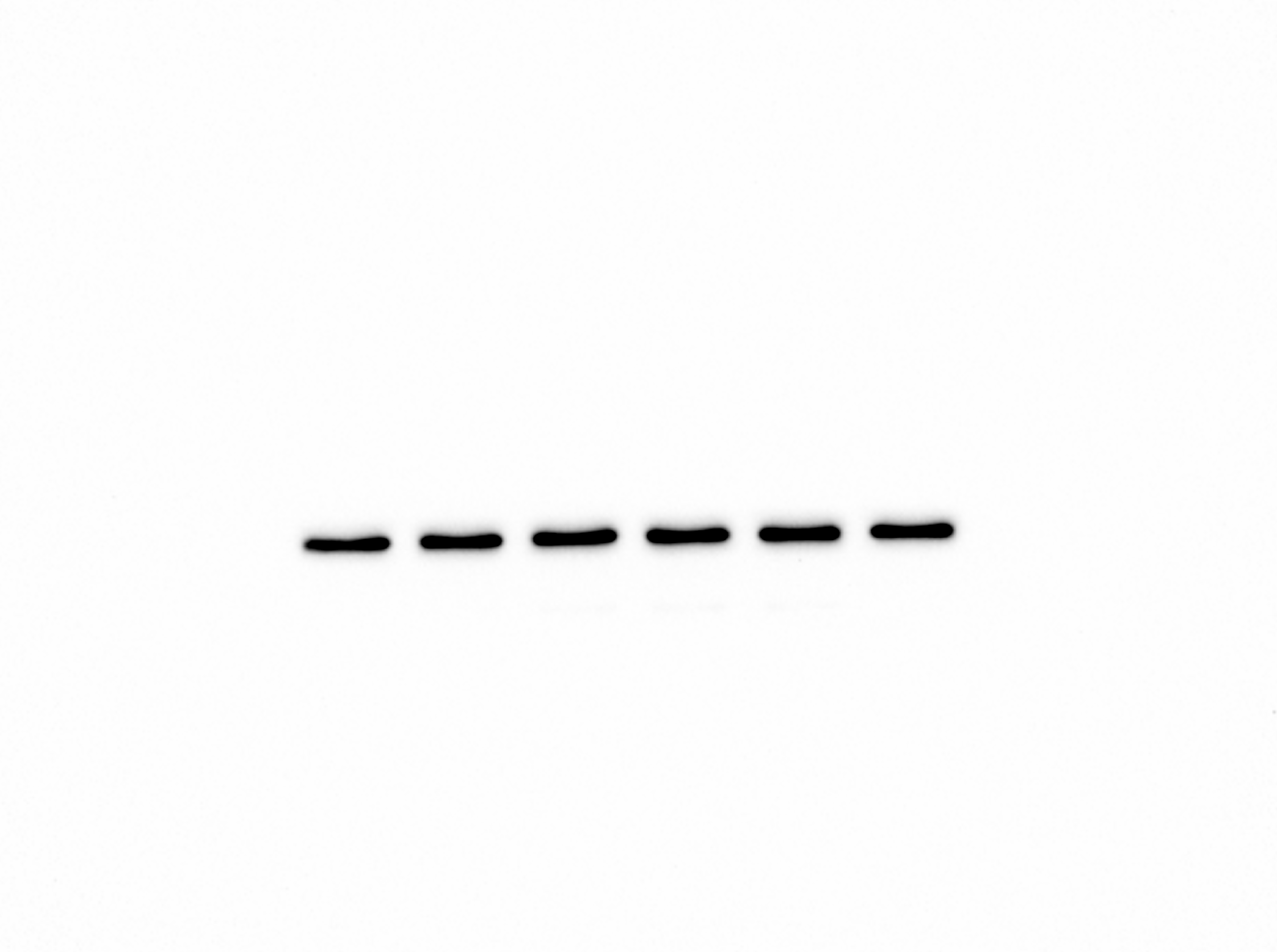

Supplement: Supplementary file 1 [file biology-12-00416-s001.zip › biology-2248156-supplementary-final/File S1-WB IMAGES-For Submission/1. MAIN MANUSCRIPT IMAGES/SH-MI/1C.SH-MI-HSPA1A-G.tif]

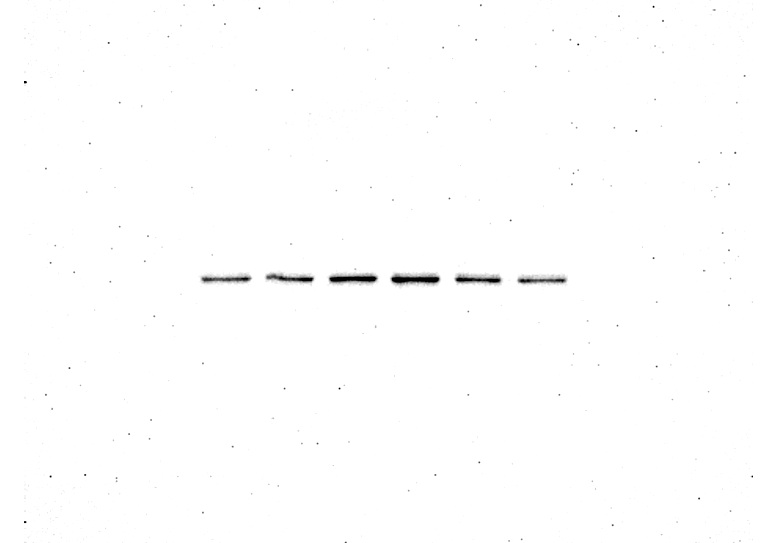

Supplement: Supplementary file 1 [file biology-12-00416-s001.zip › biology-2248156-supplementary-final/File S1-WB IMAGES-For Submission/1. MAIN MANUSCRIPT IMAGES/SH-MI/2.SH-MI-HSPA1B.tif]

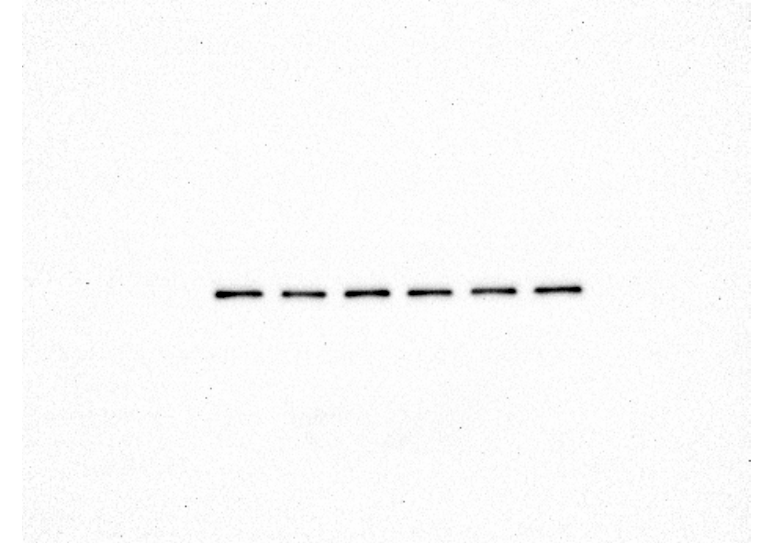

Supplement: Supplementary file 1 [file biology-12-00416-s001.zip › biology-2248156-supplementary-final/File S1-WB IMAGES-For Submission/1. MAIN MANUSCRIPT IMAGES/SH-MI/2C.SH-MI-HSPA1B-G.tif]

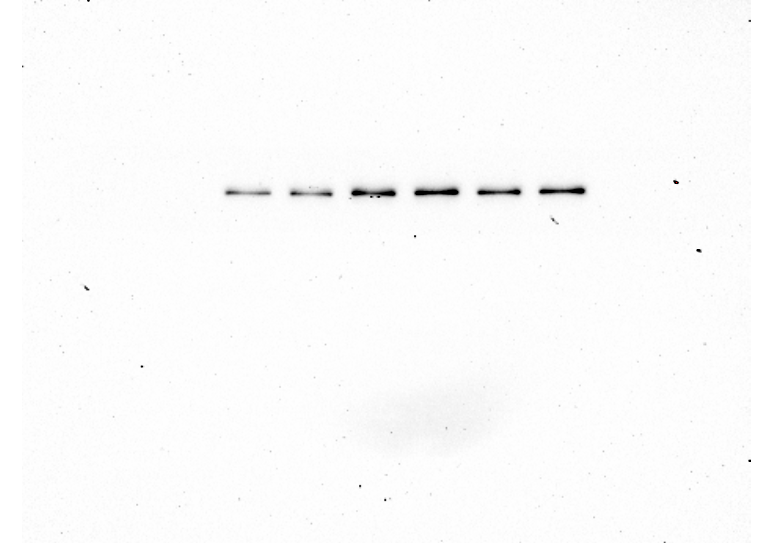

Supplement: Supplementary file 1 [file biology-12-00416-s001.zip › biology-2248156-supplementary-final/File S1-WB IMAGES-For Submission/1. MAIN MANUSCRIPT IMAGES/SH-MI/3.SH-MI-HSPA6.tif]

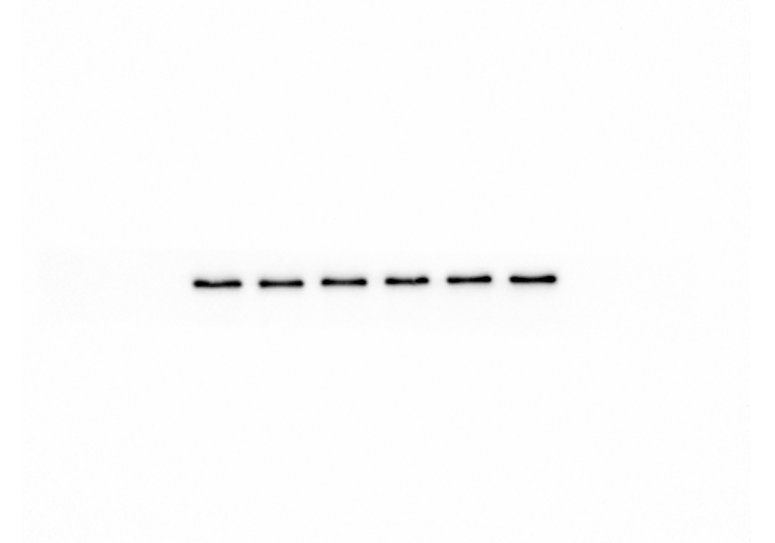

Supplement: Supplementary file 1 [file biology-12-00416-s001.zip › biology-2248156-supplementary-final/File S1-WB IMAGES-For Submission/1. MAIN MANUSCRIPT IMAGES/SH-MI/3C.SH-MI-HSPA6-G.tif]

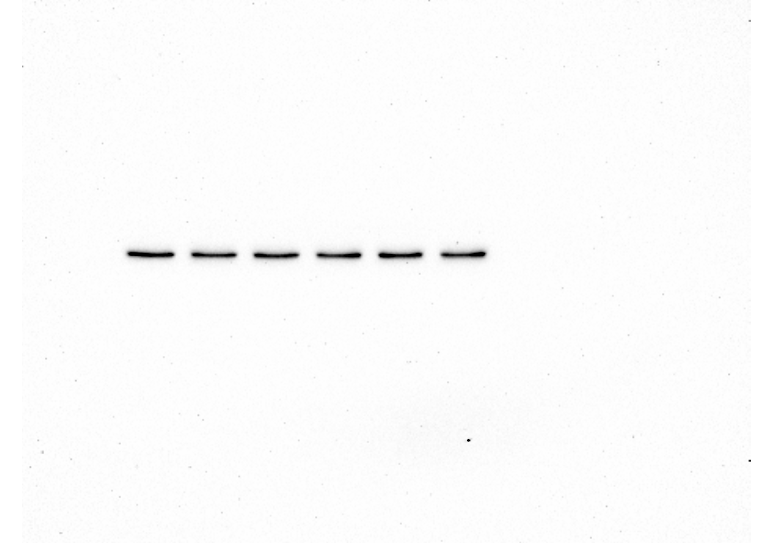

Supplement: Supplementary file 1 [file biology-12-00416-s001.zip › biology-2248156-supplementary-final/File S1-WB IMAGES-For Submission/1. MAIN MANUSCRIPT IMAGES/SH-MI/4.SH-MI-HSPA1L.tif]

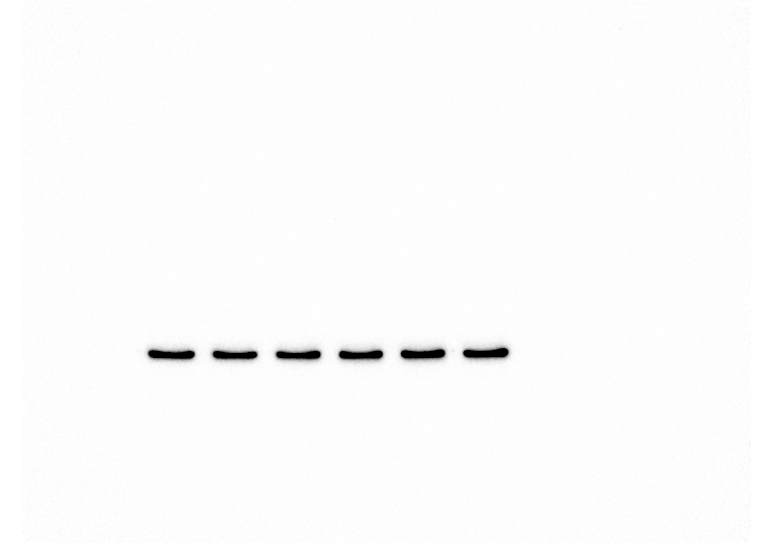

Supplement: Supplementary file 1 [file biology-12-00416-s001.zip › biology-2248156-supplementary-final/File S1-WB IMAGES-For Submission/1. MAIN MANUSCRIPT IMAGES/SH-MI/4C.SH-MI-HSPA1L-G.tif]

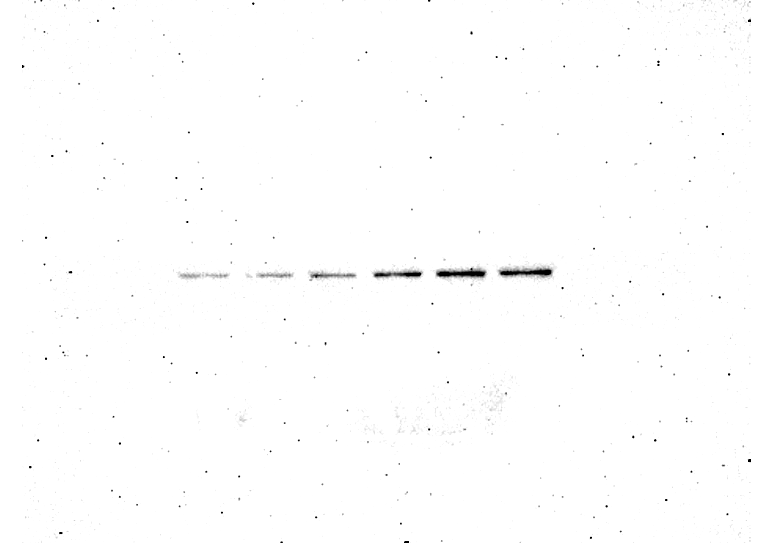

Supplement: Supplementary file 1 [file biology-12-00416-s001.zip › biology-2248156-supplementary-final/File S1-WB IMAGES-For Submission/1. MAIN MANUSCRIPT IMAGES/SH-MI/5.SH-MI-HSPA4L.tif]

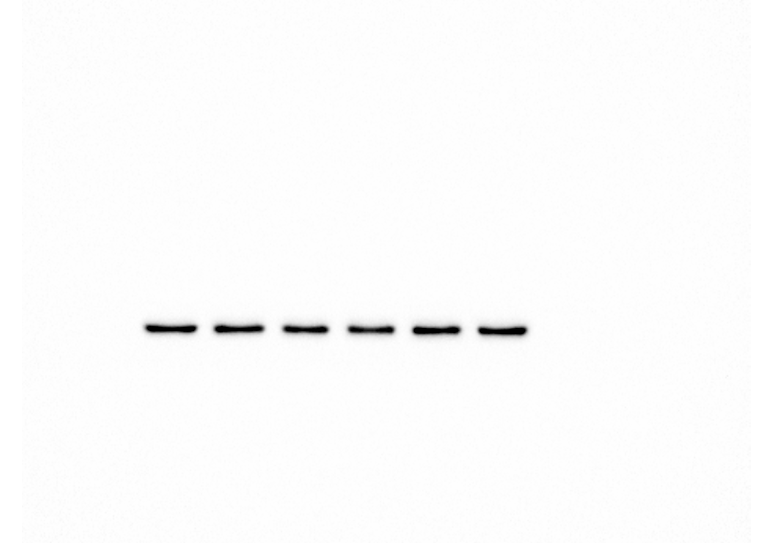

Supplement: Supplementary file 1 [file biology-12-00416-s001.zip › biology-2248156-supplementary-final/File S1-WB IMAGES-For Submission/1. MAIN MANUSCRIPT IMAGES/SH-MI/5C.SH-MI-HSPA4L-G.tif]

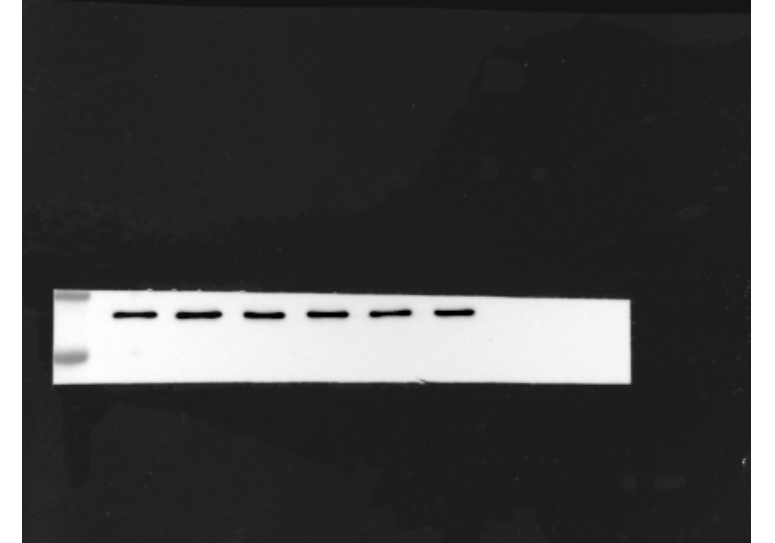

Supplement: Supplementary file 1 [file biology-12-00416-s001.zip › biology-2248156-supplementary-final/File S1-WB IMAGES-For Submission/2. MW Confirmation/1.HSPA6/HSPA6-C-With Ladder.tif]

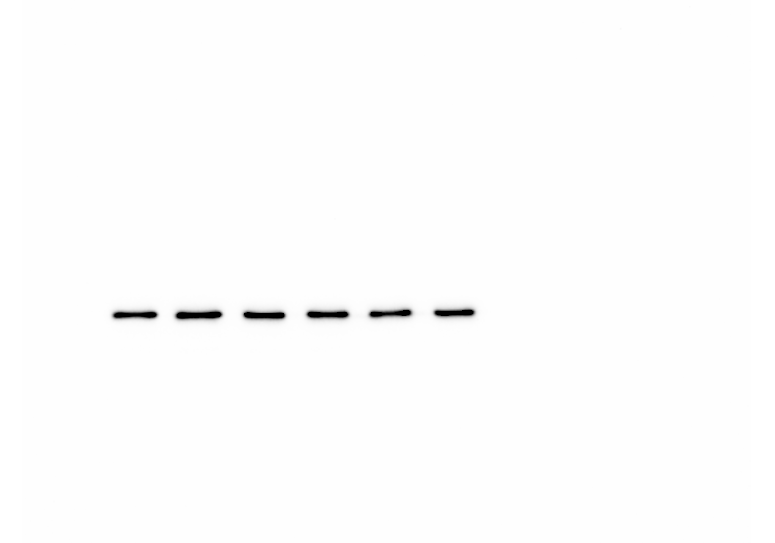

Supplement: Supplementary file 1 [file biology-12-00416-s001.zip › biology-2248156-supplementary-final/File S1-WB IMAGES-For Submission/2. MW Confirmation/1.HSPA6/HSPA6-C-Without Ladder.tif]

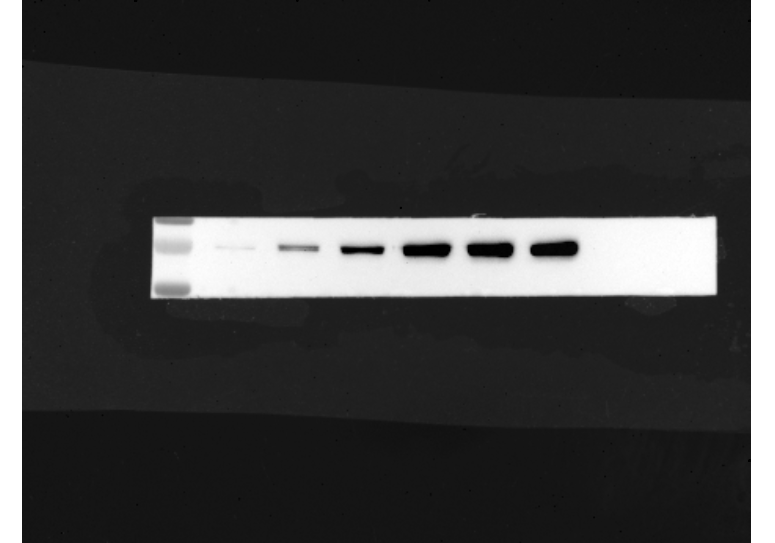

Supplement: Supplementary file 1 [file biology-12-00416-s001.zip › biology-2248156-supplementary-final/File S1-WB IMAGES-For Submission/2. MW Confirmation/1.HSPA6/HSPA6-With Ladder.tif]

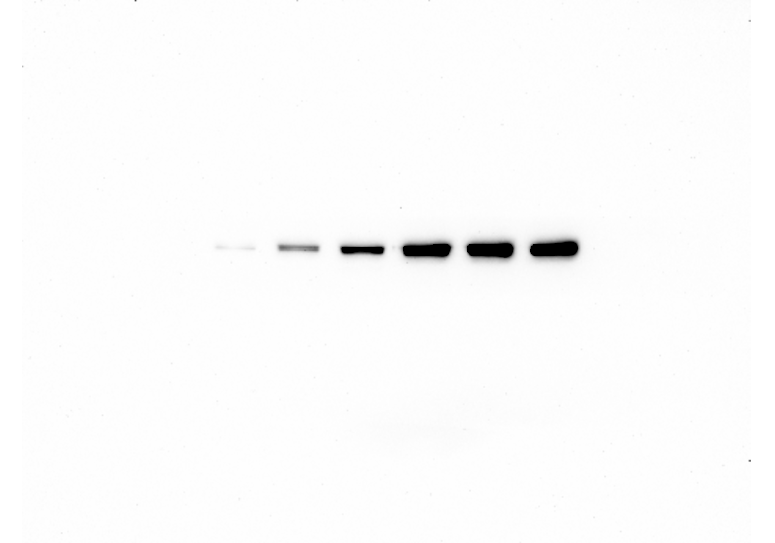

Supplement: Supplementary file 1 [file biology-12-00416-s001.zip › biology-2248156-supplementary-final/File S1-WB IMAGES-For Submission/2. MW Confirmation/1.HSPA6/HSPA6-Without Ladder.tif]

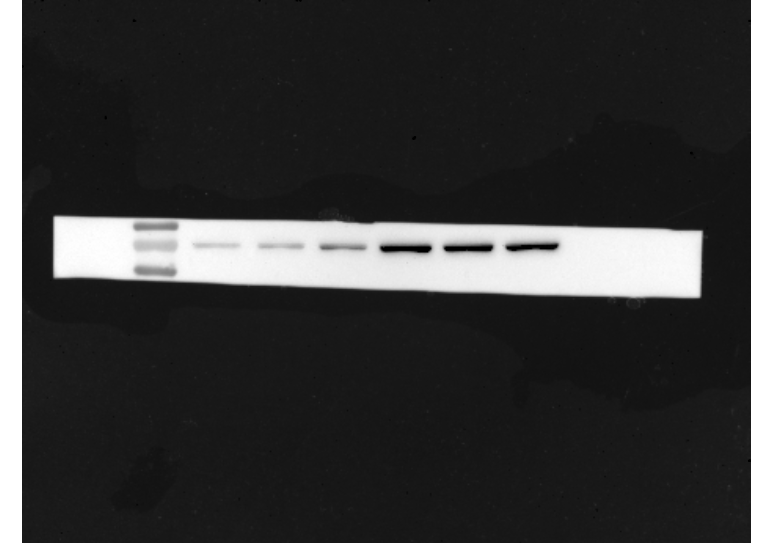

Supplement: Supplementary file 1 [file biology-12-00416-s001.zip › biology-2248156-supplementary-final/File S1-WB IMAGES-For Submission/2. MW Confirmation/2.HSPA1B/HSPA1B With Ladder.tif]

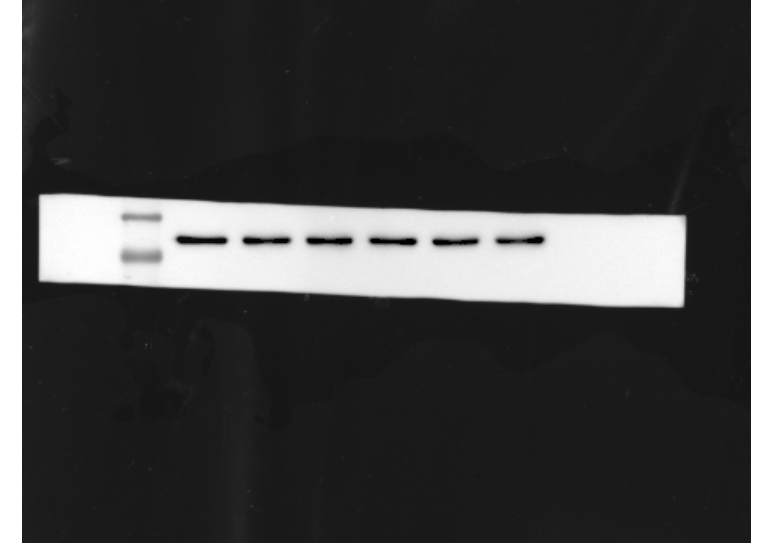

Supplement: Supplementary file 1 [file biology-12-00416-s001.zip › biology-2248156-supplementary-final/File S1-WB IMAGES-For Submission/2. MW Confirmation/2.HSPA1B/HSPA1B-C-With Ladder.tif]

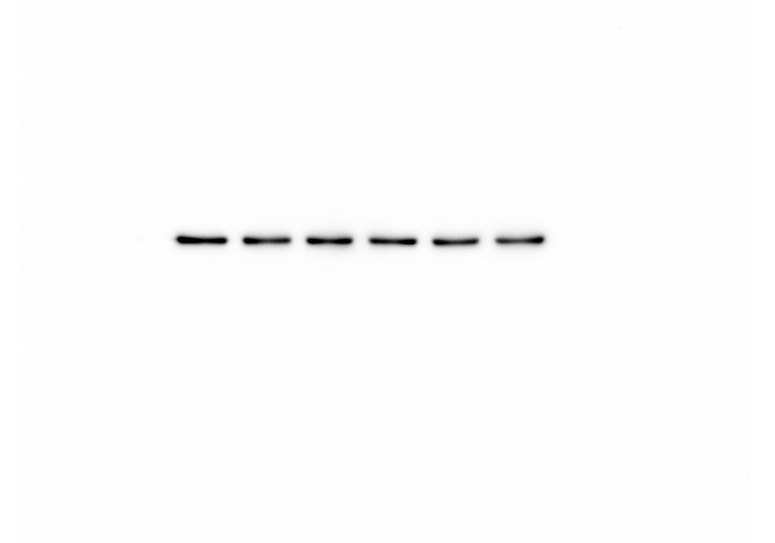

Supplement: Supplementary file 1 [file biology-12-00416-s001.zip › biology-2248156-supplementary-final/File S1-WB IMAGES-For Submission/2. MW Confirmation/2.HSPA1B/HSPA1B-C-Without Ladder.tif]

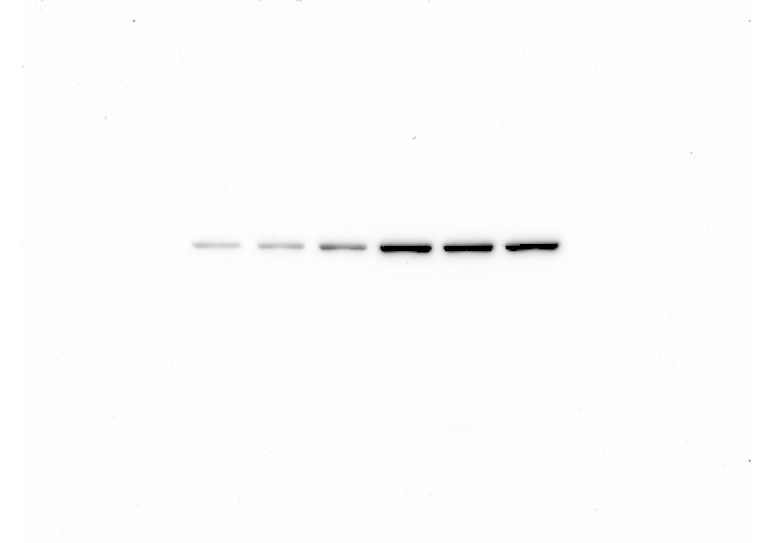

Supplement: Supplementary file 1 [file biology-12-00416-s001.zip › biology-2248156-supplementary-final/File S1-WB IMAGES-For Submission/2. MW Confirmation/2.HSPA1B/HSPA1B-Without Ladder.tif]

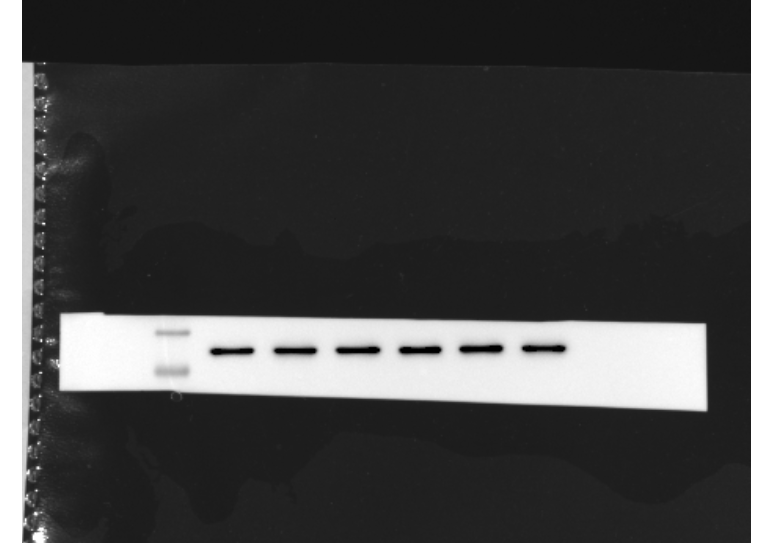

Supplement: Supplementary file 1 [file biology-12-00416-s001.zip › biology-2248156-supplementary-final/File S1-WB IMAGES-For Submission/2. MW Confirmation/3.HSPA1A/HSPA1A-C-With Ladder.tif]

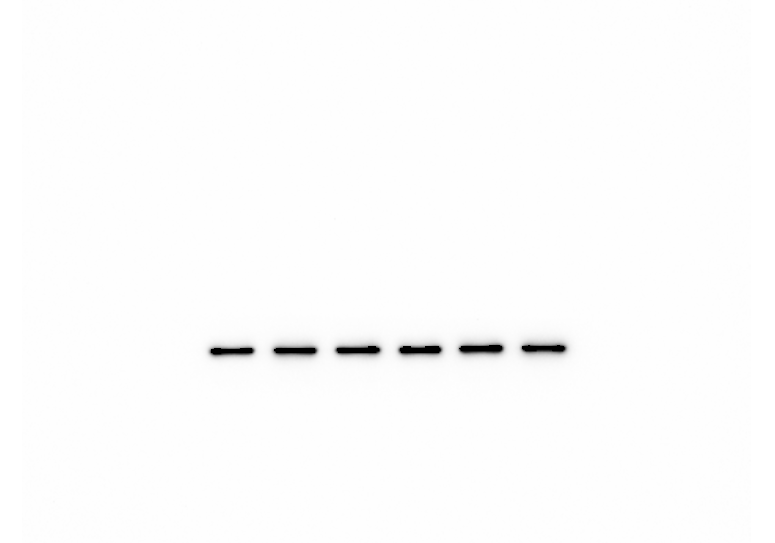

Supplement: Supplementary file 1 [file biology-12-00416-s001.zip › biology-2248156-supplementary-final/File S1-WB IMAGES-For Submission/2. MW Confirmation/3.HSPA1A/HSPA1A-C-Without Ladder.tif]

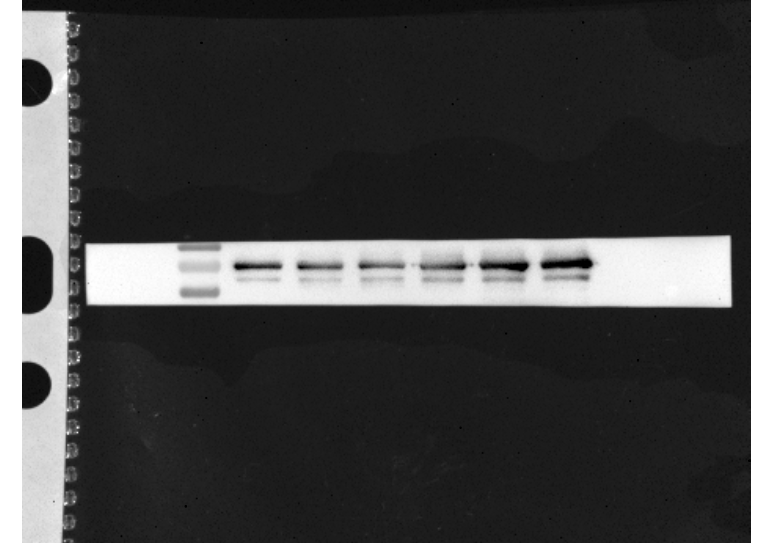

Supplement: Supplementary file 1 [file biology-12-00416-s001.zip › biology-2248156-supplementary-final/File S1-WB IMAGES-For Submission/2. MW Confirmation/3.HSPA1A/HSPA1A-With Ladder.tif]

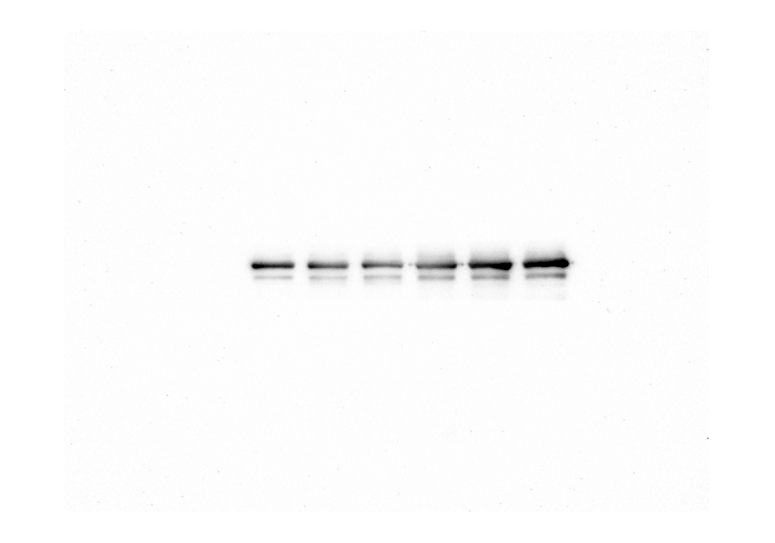

Supplement: Supplementary file 1 [file biology-12-00416-s001.zip › biology-2248156-supplementary-final/File S1-WB IMAGES-For Submission/2. MW Confirmation/3.HSPA1A/HSPA1A-Without Ladder.tif]

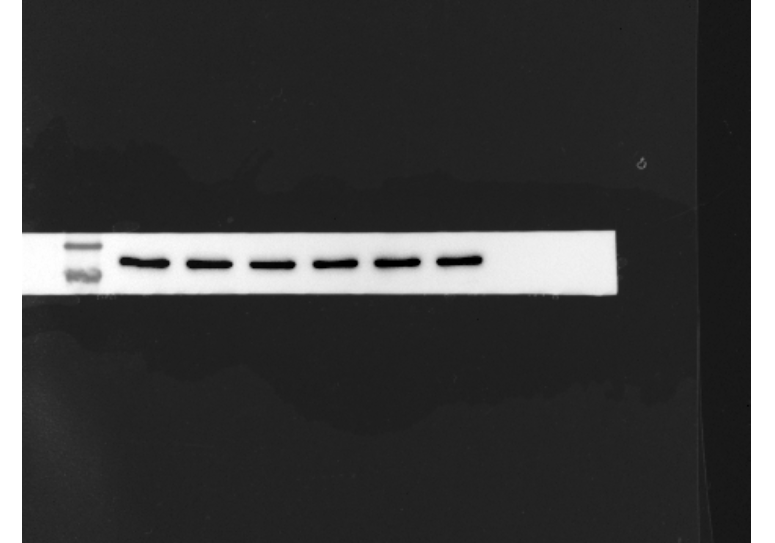

Supplement: Supplementary file 1 [file biology-12-00416-s001.zip › biology-2248156-supplementary-final/File S1-WB IMAGES-For Submission/2. MW Confirmation/4.HSPA1L/HSPA1L-C-With Ladder.tif]

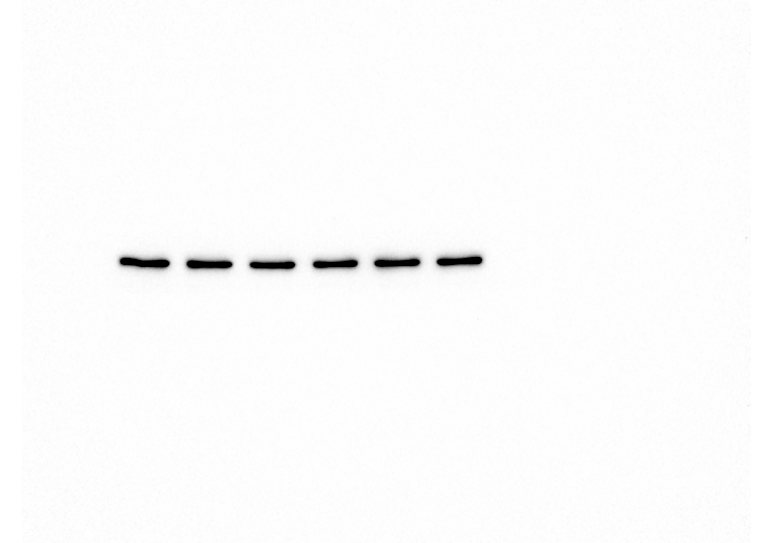

Supplement: Supplementary file 1 [file biology-12-00416-s001.zip › biology-2248156-supplementary-final/File S1-WB IMAGES-For Submission/2. MW Confirmation/4.HSPA1L/HSPA1L-C-Without Ladder.tif]

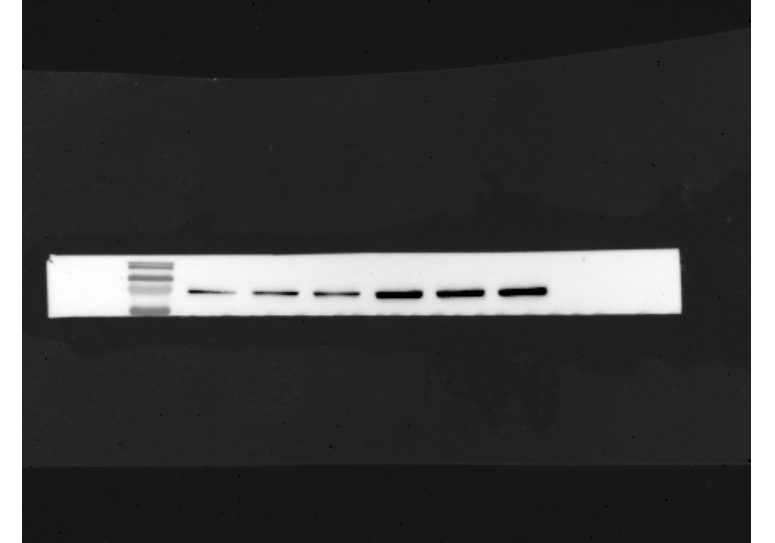

Supplement: Supplementary file 1 [file biology-12-00416-s001.zip › biology-2248156-supplementary-final/File S1-WB IMAGES-For Submission/2. MW Confirmation/4.HSPA1L/HSPA1L-With Ladder.tif]

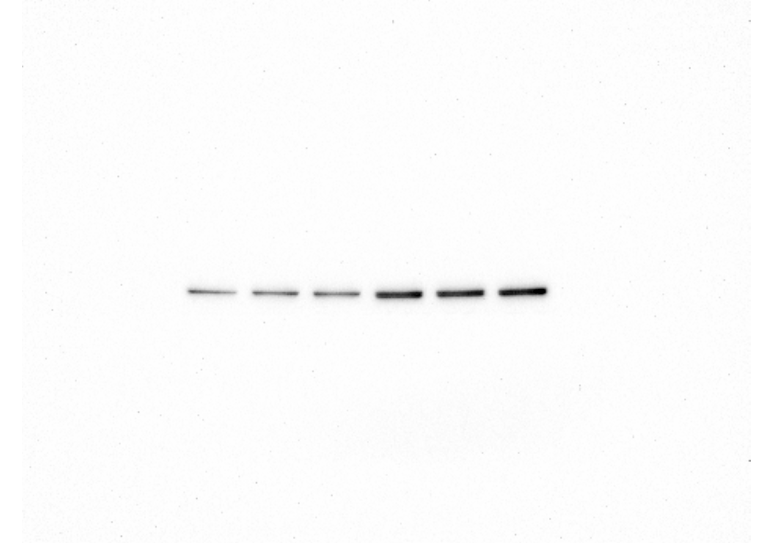

Supplement: Supplementary file 1 [file biology-12-00416-s001.zip › biology-2248156-supplementary-final/File S1-WB IMAGES-For Submission/2. MW Confirmation/4.HSPA1L/HSPA1L-Without Ladder.tif]

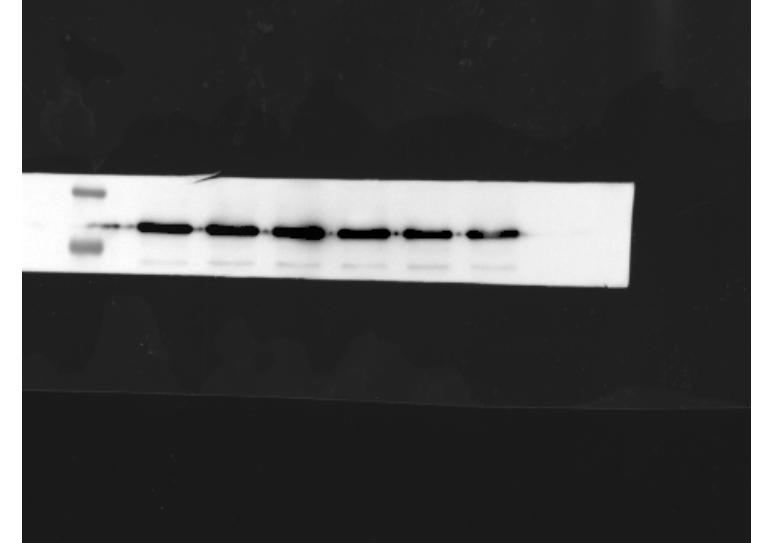

Supplement: Supplementary file 1 [file biology-12-00416-s001.zip › biology-2248156-supplementary-final/File S1-WB IMAGES-For Submission/2. MW Confirmation/5.HSPA4L/10%/HSPA4L-C-With Ladder.tif]

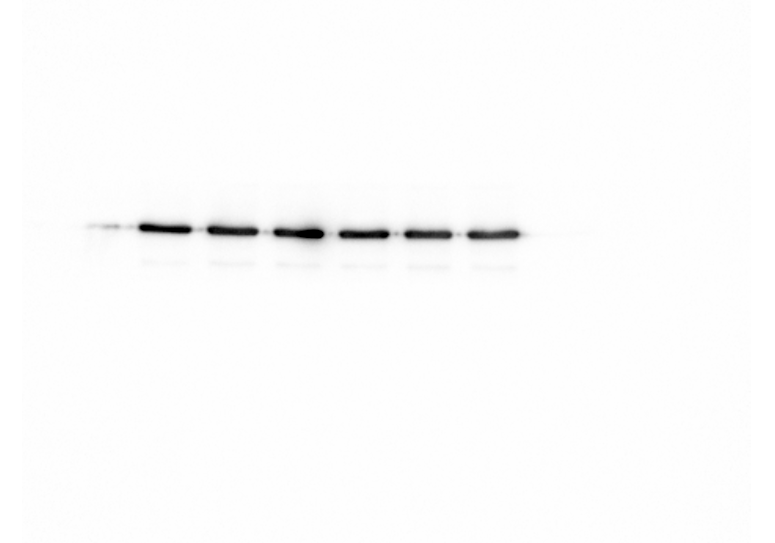

Supplement: Supplementary file 1 [file biology-12-00416-s001.zip › biology-2248156-supplementary-final/File S1-WB IMAGES-For Submission/2. MW Confirmation/5.HSPA4L/10%/HSPA4L-C-Without Ladder.tif]

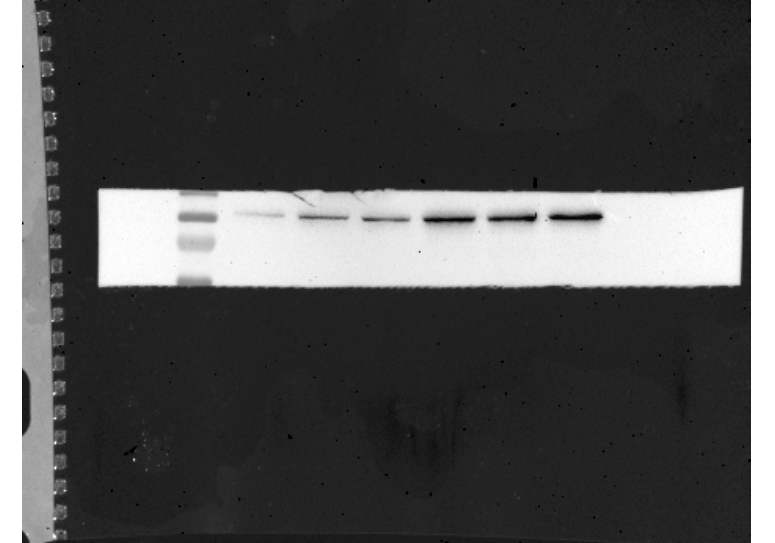

Supplement: Supplementary file 1 [file biology-12-00416-s001.zip › biology-2248156-supplementary-final/File S1-WB IMAGES-For Submission/2. MW Confirmation/5.HSPA4L/10%/HSPA4L-With Ladder.tif]

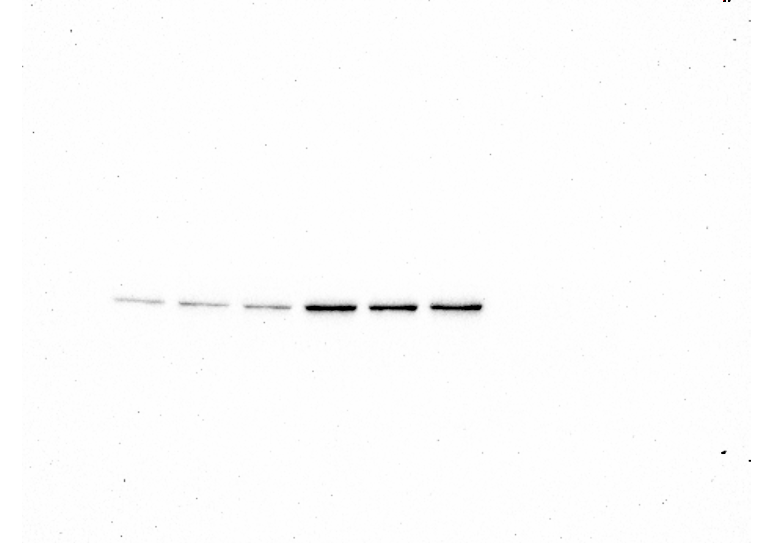

Supplement: Supplementary file 1 [file biology-12-00416-s001.zip › biology-2248156-supplementary-final/File S1-WB IMAGES-For Submission/2. MW Confirmation/5.HSPA4L/10%/HSPA4L-Without Ladder.tif]

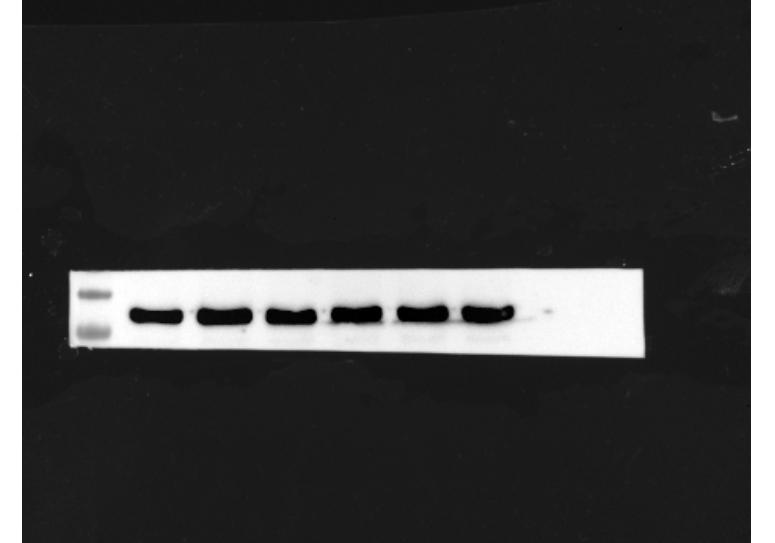

Supplement: Supplementary file 1 [file biology-12-00416-s001.zip › biology-2248156-supplementary-final/File S1-WB IMAGES-For Submission/2. MW Confirmation/5.HSPA4L/12%/HSPA4L-C-With Ladder.tif]

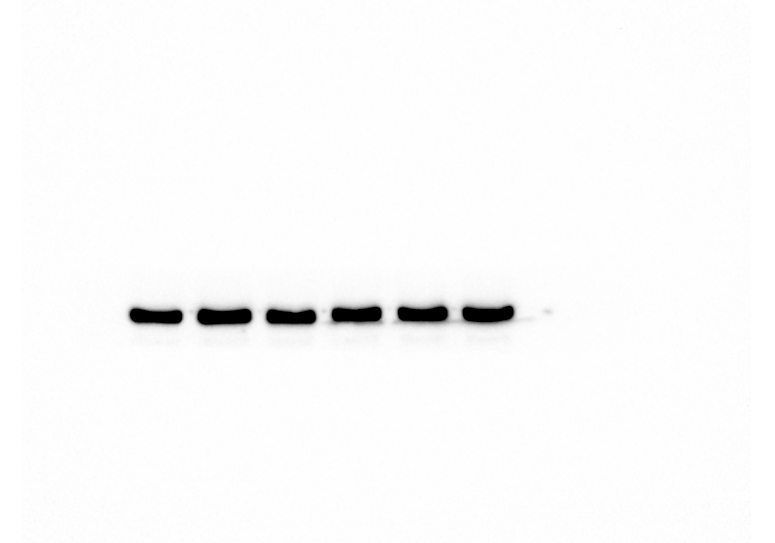

Supplement: Supplementary file 1 [file biology-12-00416-s001.zip › biology-2248156-supplementary-final/File S1-WB IMAGES-For Submission/2. MW Confirmation/5.HSPA4L/12%/HSPA4L-C-Without Ladder.tif]

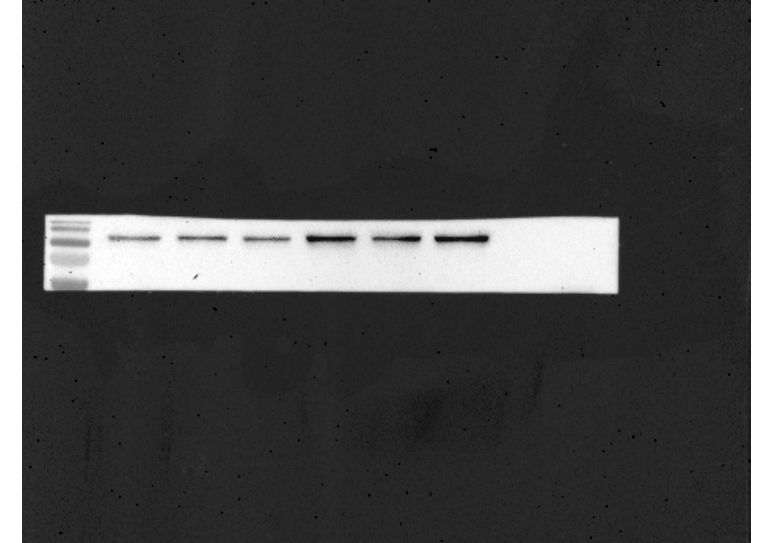

Supplement: Supplementary file 1 [file biology-12-00416-s001.zip › biology-2248156-supplementary-final/File S1-WB IMAGES-For Submission/2. MW Confirmation/5.HSPA4L/12%/HSPA4L-With Ladder.tif]

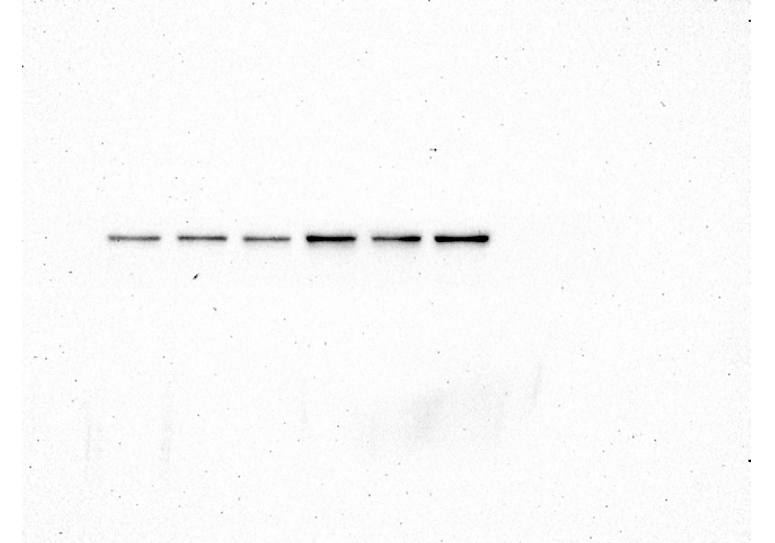

Supplement: Supplementary file 1 [file biology-12-00416-s001.zip › biology-2248156-supplementary-final/File S1-WB IMAGES-For Submission/2. MW Confirmation/5.HSPA4L/12%/HSPA4L-Without Ladder.tif]

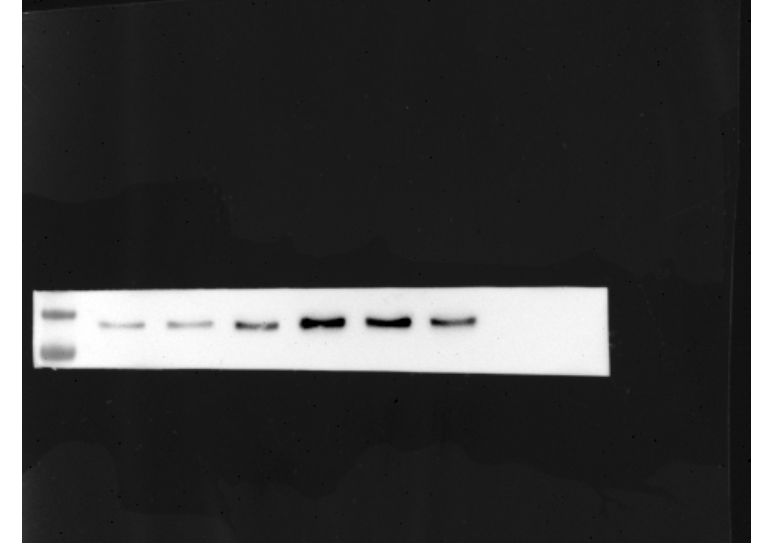

Supplement: Supplementary file 1 [file biology-12-00416-s001.zip › biology-2248156-supplementary-final/File S1-WB IMAGES-For Submission/2. MW Confirmation/6.DNAJB1/DNAJB1-With Ladder.tif]

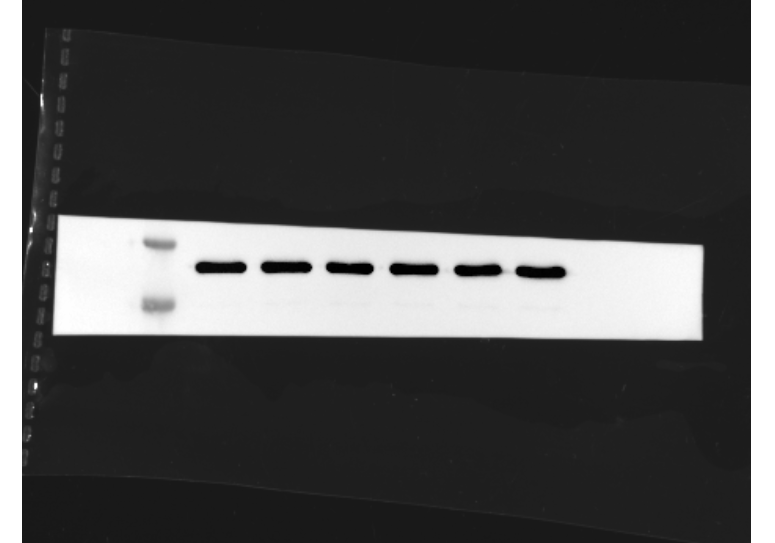

Supplement: Supplementary file 1 [file biology-12-00416-s001.zip › biology-2248156-supplementary-final/File S1-WB IMAGES-For Submission/2. MW Confirmation/7. BAG3/BAG3-C-With Ladder.tif]

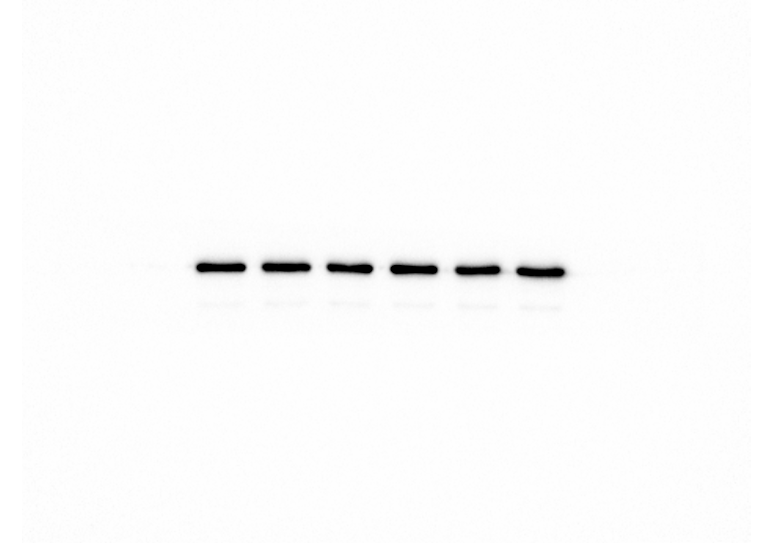

Supplement: Supplementary file 1 [file biology-12-00416-s001.zip › biology-2248156-supplementary-final/File S1-WB IMAGES-For Submission/2. MW Confirmation/7. BAG3/BAG3-C-Without Ladder.tif]

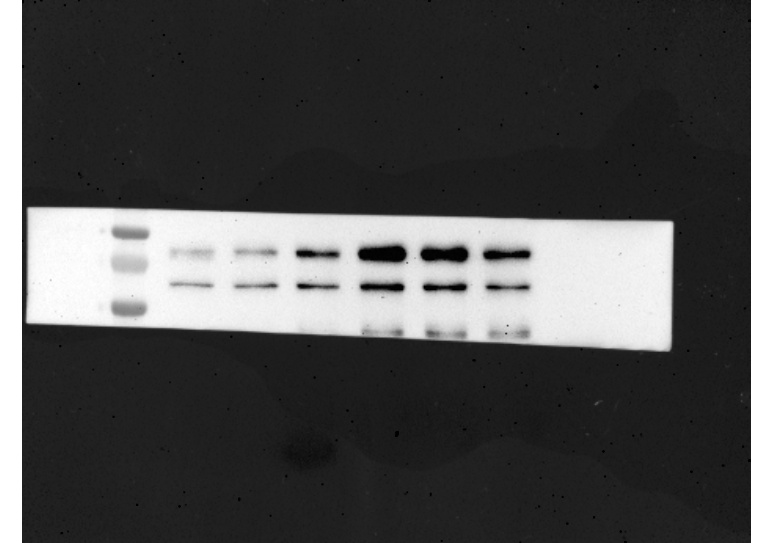

Supplement: Supplementary file 1 [file biology-12-00416-s001.zip › biology-2248156-supplementary-final/File S1-WB IMAGES-For Submission/2. MW Confirmation/7. BAG3/BAG3-With Ladder.tif]

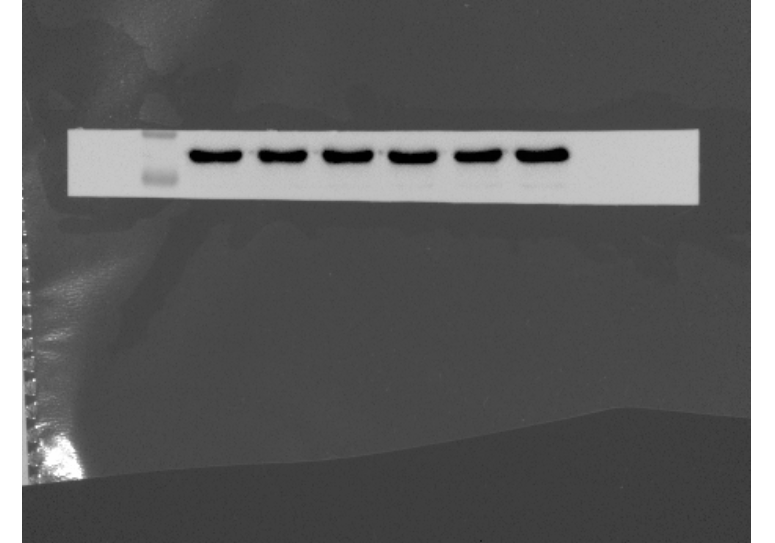

Supplement: Supplementary file 1 [file biology-12-00416-s001.zip › biology-2248156-supplementary-final/File S1-WB IMAGES-For Submission/2. MW Confirmation/8.HSPH1/HSPH1-C-With Ladder.tif]

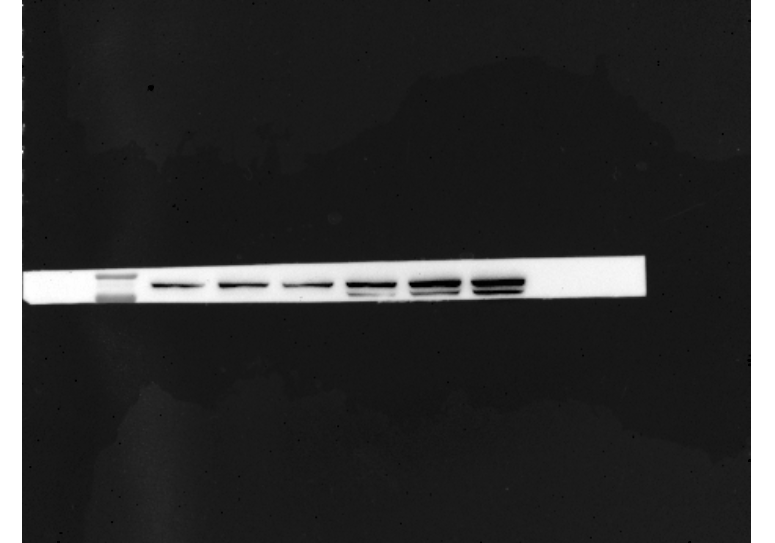

Supplement: Supplementary file 1 [file biology-12-00416-s001.zip › biology-2248156-supplementary-final/File S1-WB IMAGES-For Submission/2. MW Confirmation/8.HSPH1/HSPH1-With Ladder.tif]

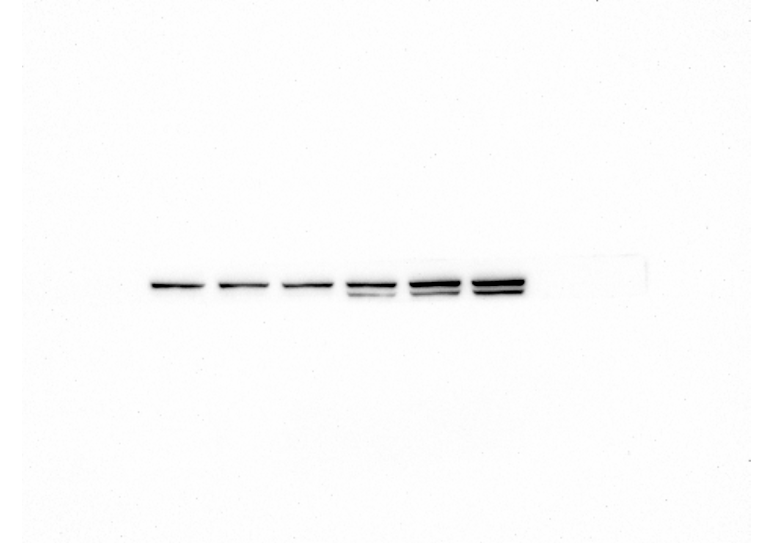

Supplement: Supplementary file 1 [file biology-12-00416-s001.zip › biology-2248156-supplementary-final/File S1-WB IMAGES-For Submission/2. MW Confirmation/8.HSPH1/HSPH1-Without Ladder.tif]

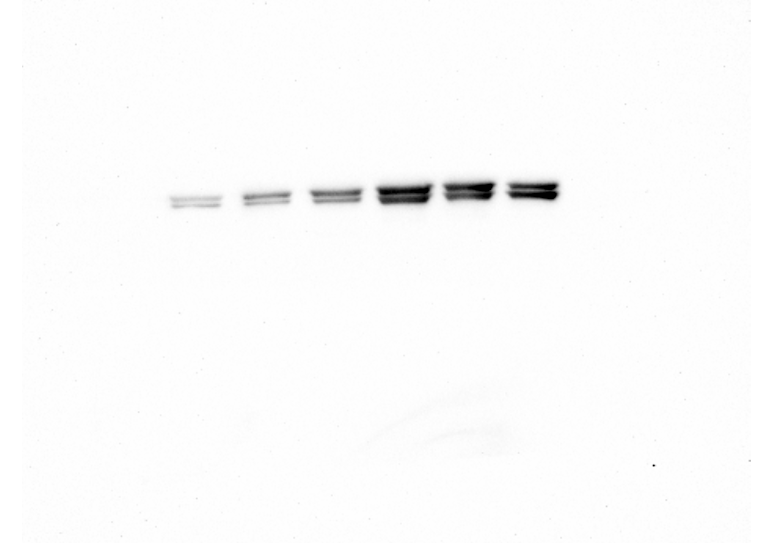

Supplement: Supplementary file 1 [file biology-12-00416-s001.zip › biology-2248156-supplementary-final/File S1-WB IMAGES-For Submission/3.Antibody Crossreactivity/1.HSPA6.tif]

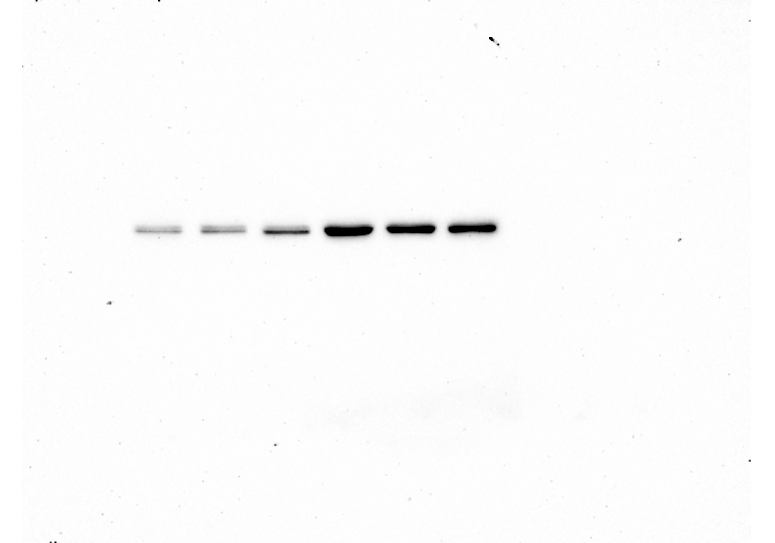

Supplement: Supplementary file 1 [file biology-12-00416-s001.zip › biology-2248156-supplementary-final/File S1-WB IMAGES-For Submission/3.Antibody Crossreactivity/2.HSPA1B.tif]

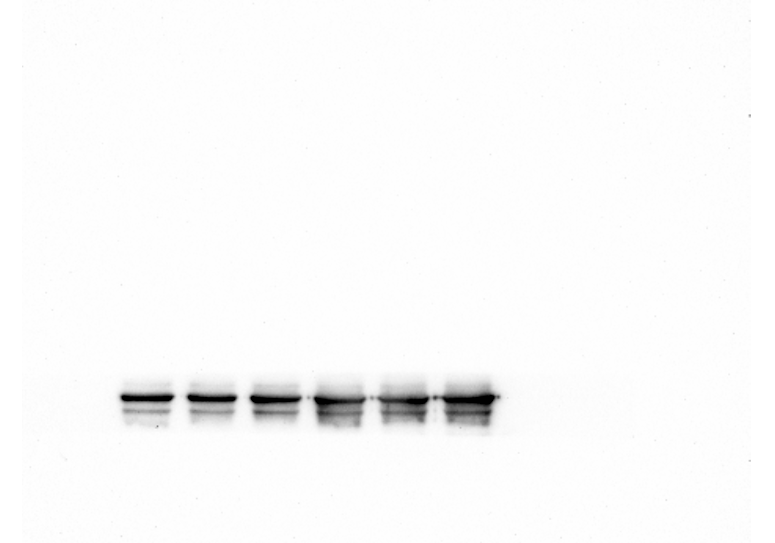

Supplement: Supplementary file 1 [file biology-12-00416-s001.zip › biology-2248156-supplementary-final/File S1-WB IMAGES-For Submission/3.Antibody Crossreactivity/3.HSPA1A.tif]
